# Discovery of novel drug-like antitubercular hits targeting the MEP pathway enzyme DXPS by strategic application of ligand-based virtual screening†

**DOI:** 10.1039/d2sc02371g

**Published:** 2022-08-08

**Authors:** Di Zhu, Sandra Johannsen, Tiziana Masini, Céline Simonin, Jörg Haupenthal, Boris Illarionov, Anastasia Andreas, Mahendra Awale, Robin M. Gierse, Tridia van der Laan, Ramon van der Vlag, Rita Nasti, Mael Poizat, Eric Buhler, Norbert Reiling, Rolf Müller, Markus Fischer, Jean-Louis Reymond, Anna K. H. Hirsch

**Affiliations:** Helmholtz Institute for Pharmaceutical Research Saarland (HIPS) – Helmholtz Centre for Infection Research (HZI), Campus Building E8.1 66123 Saarbrücken Germany anna.hirsch@helmholtz-hips.de; Department of Pharmacy, Saarland University, Campus Building E8.1 66123 Saarbrücken Germany; Stratingh Institute for Chemistry, University of Groningen Nijenborgh 7 9747 AG Groningen The Netherlands; Department of Chemistry, Biochemistry and Pharmaceutical Sciences, University of Bern Freiestrasse 3 3012 Bern Switzerland jean-louis.reymond@unibe.ch; Hamburg School of Food Science, Institute of Food Chemistry Grindelallee 117 20146 Hamburg Germany; Department of Mycobacteria, National Institute of Public Health and the Environment (RIVM), Diagnostics and Laboratory Surveillance (IDS) Infectious Diseases Research Antonie van Leeuwenhoeklaan 9 3721 MA Bilthoven The Netherlands; Symeres Kadijk 3 9747 AT Groningen The Netherlands; Laboratoire Matière et Systèmes Complexes (MSC), UMR CNRS 7057, Université Paris Cité Bâtiment Condorcet 75205 Paris Cedex 13 France; RG Microbial Interface Biology, Research Center Borstel, Leibniz Lung Center Borstel Germany; German Center for Infection Research (DZIF), Partner Site Hamburg-Lübeck-Borstel-Riems Borstel Germany; Helmholtz International Lab for Anti-infectives Campus Building E8.1 66123 Saarbrücken Germany

## Abstract

In the present manuscript, we describe how we successfully used ligand-based virtual screening (LBVS) to identify two small-molecule, drug-like hit classes with excellent ADMET profiles against the difficult to address microbial enzyme 1-deoxy-d-xylulose-5-phosphate synthase (DXPS). In the fight against antimicrobial resistance (AMR), it has become increasingly important to address novel targets such as DXPS, the first enzyme of the 2-*C*-methyl-d-erythritol-4-phosphate (MEP) pathway, which affords the universal isoprenoid precursors. This pathway is absent in humans but essential for pathogens such as *Mycobacterium tuberculosis*, making it a rich source of drug targets for the development of novel anti-infectives. Standard computer-aided drug-design tools, frequently applied in other areas of drug development, often fail for targets with large, hydrophilic binding sites such as DXPS. Therefore, we introduce the concept of pseudo-inhibitors, combining the benefits of pseudo-ligands (defining a pharmacophore) and pseudo-receptors (defining anchor points in the binding site), for providing the basis to perform a LBVS against *M. tuberculosis* DXPS. Starting from a diverse set of reference ligands showing weak inhibition of the orthologue from *Deinococcus radiodurans* DXPS, we identified three structurally unrelated classes with promising *in vitro* (against *M. tuberculosis* DXPS) and whole-cell activity including extensively drug-resistant strains of *M. tuberculosis*. The hits were validated to be specific inhibitors of DXPS and to have a unique mechanism of inhibition. Furthermore, two of the hits have a balanced profile in terms of metabolic and plasma stability and display a low frequency of resistance development, making them ideal starting points for hit-to-lead optimization of antibiotics with an unprecedented mode of action.

## Abstract 2

We identified two drug-like antitubercular hits with submicromolar inhibition constants against the target 1-deoxy-d-xylulose-5-phosphate synthase (DXPS) with a new mode of action and promising activity against drug-resistant tuberculosis.

## Graphical abstract

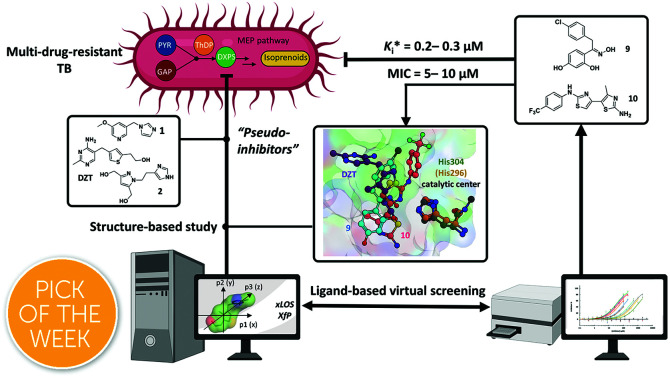

## Introduction

The discovery of novel antibiotics with unprecedented mode of action is increasingly important due to the fast development of antimicrobial resistance (AMR), but it has been notoriously difficult to expand the pool of targets and active compounds in the past decades.^1^ Conventional methods such as screening of natural-product libraries and their optimization have resulted in most of the antibiotics on the market today, but to keep up with AMR, new techniques need to be employed.^2,3^ Standard tools for oral drug discovery against eukaryotic targets, like computer-aided drug design (CADD) and screening of combinatorial libraries, are mostly ineffective for the development of anti-infective drugs.^4,5^ Compound libraries often follow Lipinski's “rule-of-5” (Ro5) but since antibiotic targets tend to have large, hydrophilic binding sites, these libraries rarely result in new hits.^6,7^ Although great efforts have been made to expand the limits of the Ro5, a lot still needs to be done to improve and adjust known tools to facilitate fast and effective discovery and development of drug-like antibiotics.^8–12^

**Fig. 1:**
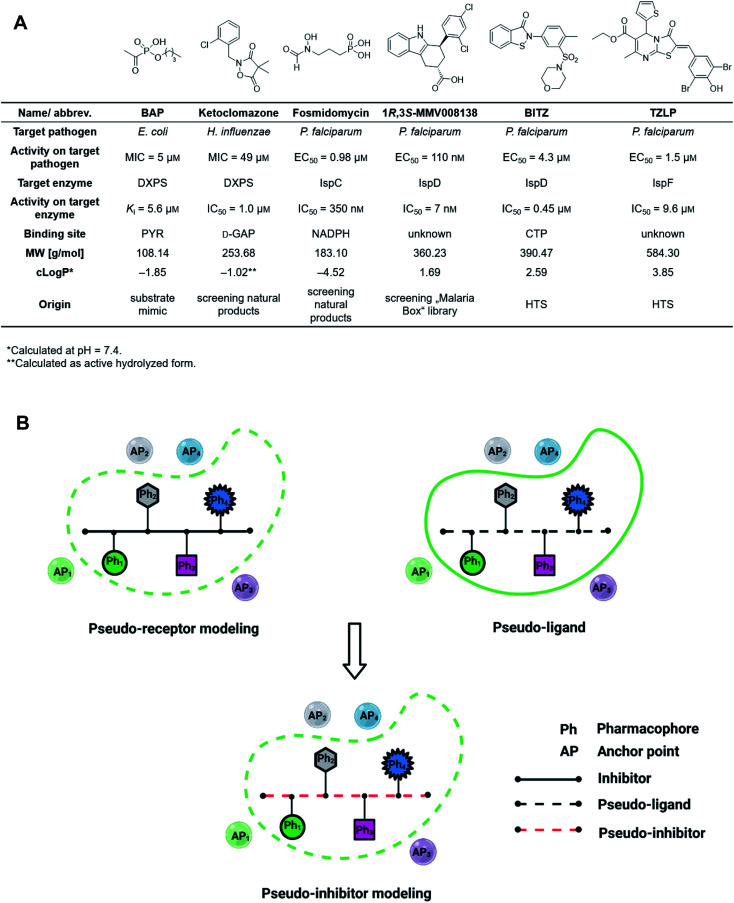
(A) Representative inhibitors of the 2-*C*-methyl-d-erythritol-4-phosphate pathway showing promising on-target and cell-based activity against certain relevant pathogens. MIC = minimum inhibitory concentration, EC_50_ = half maximal effective concentration, NADPH = nicotinamide adenine dinucleotide phosphate, CTP = cytidine triphosphate, HTS = high-throughput screening. (B) The concept of pseudo-inhibitors, *i.e.*, the combination of pseudo-receptor modeling and pseudo-ligands. Created with https://BioRender.com.

The 2-*C*-methyl-d-erythritol-4-phosphate (MEP) pathway is essential for the biosynthesis of the universal isoprenoid precursors isopentenyl diphosphate (IDP) and dimethylallyl diphosphate (DMADP) in many important pathogens, including *Plasmodium falciparum*, *Haemophilus influenzae*, *Mycobacterium tuberculosis* and *Escherichia coli* but its absence in humans makes it a source of validated anti-infective targets.^13–16^ The pathway is composed of seven enzymes, with most of the substrates and cofactors being phosphorylated and involved in metal interactions. This makes all MEP enzymes challenging drug targets due to a lack of hydrophobic anchor points (APs) in these pockets.^17^ Hydrophobic inhibitors being in compliance with the Ro5 are less likely to gain sufficient binding affinity to outcompete the tight-binding, natural binders while retaining their drug-like properties. In consequence, it is not surprising that up to date, though there is an increasing number of (co)crystal structures of MEP pathway enzymes, only a few promising inhibitor scaffolds were found with *in vitro* and cell-based anti-infective activity against MEP pathway-utilizing pathogens (Fig. 1A).^14,16^ Butylacetylphosphonate (BAP), ketoclomazone and fosmidomycin are hydrophilic small molecules beyond the Ro5 that mimic the tight-binding nature of substrates or cofactors.^18–26^ 1*R*,3*S*-MMV008138, BITZ and TZLP are drug-like molecules based on their physicochemical properties.^15,27–29^ BITZ (possibly also TZLP) does not function through typical lock–key interaction, but covalently inhibits the target enzyme by reaction with a cysteine in the active site. Most of the inhibitors are in hit or early lead stage development except fosmidomycin, which is in phase II clinical trials to treat uncomplicated malaria.^30^ These outcomes are not meeting the high expectations that were raised upon the discovery of the MEP pathway in the 1990s. Here, antibiotic development appears to be trapped in a limited system, where the discovery of novel hits/leads depends too heavily on direct screening, while rational methods, such as computational approaches are underrepresented. Although the MEP pathway enzymes are considered encouraging targets to obtain novel anti-infectives to fight against drug-resistant tuberculosis, no hits/leads have been verified against *M. tuberculosis* yet.^16,31^ Therefore, our main goal is to develop novel antitubercular agents targeting the MEP pathway, *via* virtual screening (VS) guided by rational drug-design principles and demonstrate that it is possible to use these methods successfully in antibiotic research.^32^

The first enzyme in the MEP pathway is 1-deoxy-d-xylulose-5-phosphate synthase (DXPS), which catalyzes the thiamine diphosphate (ThDP)-dependent decarboxylation of pyruvate (PYR) and addition of d-glyceraldehyde 3-phosphate (d-GAP) to afford 1-deoxy-d-xylulose-5-phosphate (DXP).^33,34^ Unlike other ThDP-dependent enzymes, DXPS catalyzes a random sequential mechanism, requiring ternary complex formation *en route* to DXP. This mechanism has been investigated thoroughly for *Deinococcus radiodurans* (*Dr*)DXPS and *E. coli* (*Ec*)DXPS and inhibition of *M. tuberculosis* (*Mt*)DXPS by BAP, targeting the large active site of DXPS, suggests that *Mt*DXPS shares these mechanistic characteristics.^24,33,34^ The tight-binding nature of ThDP, the highly hydrophilic metal ion-containing binding sites, the complex mechanism and the uncertainty of substrate binding throughout the reaction mechanism are challenging features and reasons why the standard structure-based VS (SBVS) algorithms have been less reliable on DXPS.^35–38^

The prerequisite for a conventional ligand-based virtual screening (LBVS) campaign is to have at least one bioactive reference against the target, but since no known hydrophobic, small-molecule inhibitors against *Mt*DXPS were available to directly initialize the LBVS, we chose a few weak drug-like inhibitors against the structurally similar *Dr*DXPS instead.^37,39^

For the first time in literature, we took advantage of structure-based modeling and ligand-based alignment by combining the concepts of pseudo-receptor and pseudo-ligands, and defined the new term “pseudo-inhibitor” (Fig. 1B).^40–42^ A pseudo-receptor is built based on true inhibitors against the target enzyme, where the APs come from homology modeling or 3D-quantitative structure–activity relationships (QSARs) and the real receptor structure is not known. In contrast, pseudo-ligands are virtual inhibitors that have never been tested as inhibitors of a target, and are adducts of pharmacophores proposed based on the (co)crystal structure of the target enzyme. In both cases, knowledge gaps are overcome by computational analyses of the known entities, either active ligands or known crystal structures. For both pseudo-receptor- and pseudo-ligand-based methods, pharmacophore mapping and validation are necessary to assure the accuracy. Our proposed concept of pseudo-inhibitors is combining both methods. As a starting point, compounds that are not active on the target, but are true inhibitors of a structurally close homologue or orthologue of the target, are used to identify the key APs and pharmacophores. Then, if the APs are conserved between the homologue and the target of interest, we assume the pseudo-inhibitors bear the same interaction on both proteins and work as a guideline for LBVS to help us find true inhibitors against the target with the proposed interaction. We propose that this method is especially powerful, if the pseudo-inhibitors have a unique mode of inhibition (MOI), like targeting the catalytic or allosteric center that is conserved among homologues.

## Results and discussion

### Selection of pseudo-inhibitors

**Fig. 2:**
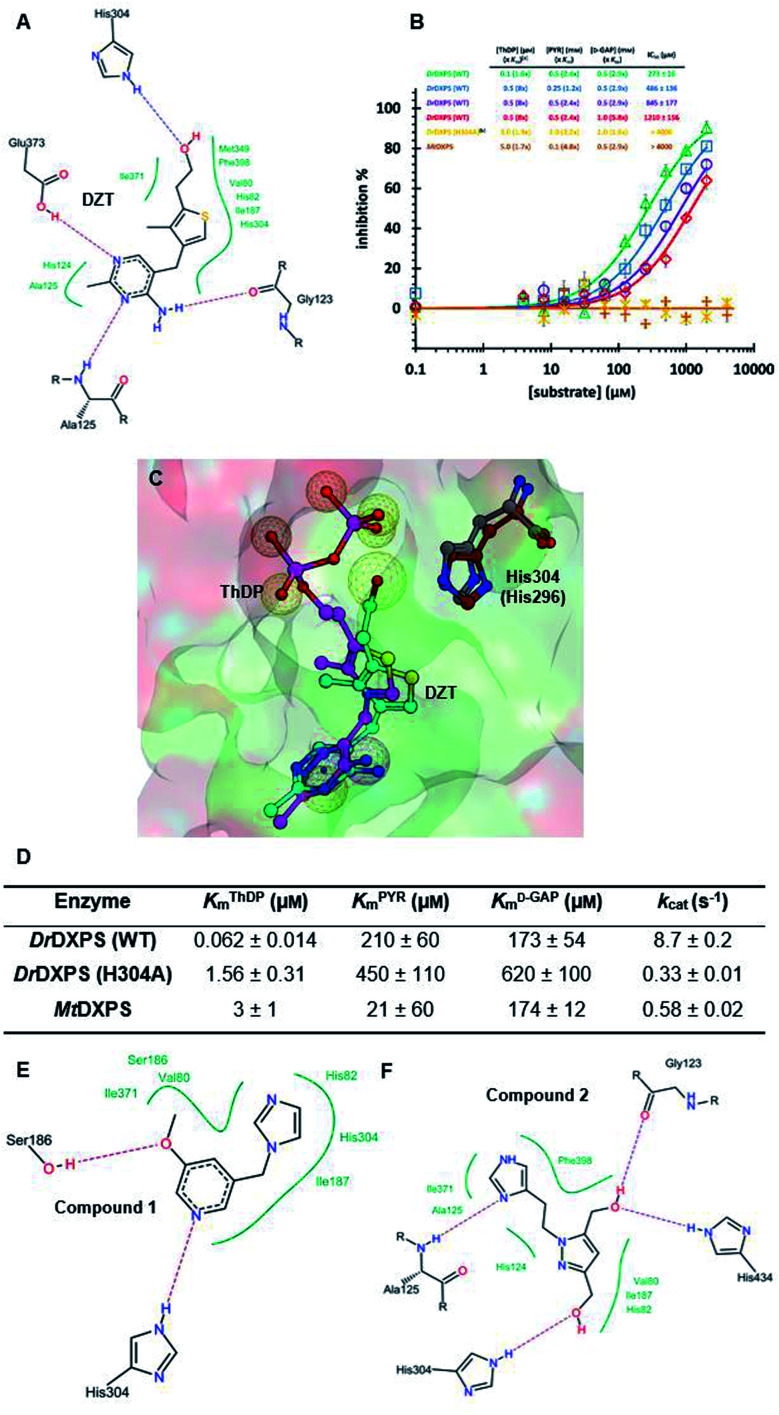
(A) Top-ranked docking pose of deazathiamine (DZT) with crystal structure of *Dr*DXPS (PDBID: 2O1X).^38^*ApoDr*DXPS was prepared by directly removing the cocrystalized ThDP, and docking was performed in presence of Mg^2+^ (same for the other docking operations in this paper). (B) Mode-of-inhibition study of DZT against *Dr*DXPS and dose–response curves of DZT against *Mt*DXPS and H304A mutant. [a] To make the comparison more convenient, we also present the value of [*S*]/*K*_m_, as (*x K*_m_). [b] The assay condition of *Dr*DXPS (H304A) are the lowest working concentrations of ThDP, PYR and d-GAP for the mutant, to maximize the chance of observing inhibition of the inhibitors. Color code: reference condition: purple; varying [ThDP]: green; varying [PYR]: blue; varying [d-GAP]: red. (C) Pharmacophore view of ThDP and DZT. Color code: C-skeleton: DZT: light blue; ThDP: magenta; His304 (*Dr*DXPS): gray; His296 (*Mt*DXPS): brown. Surface: hydrophobic site: green; hydrophilic site: red. (D) Kinetic characterization of *Dr*DXPS (WT), *Dr*DXPS (H304A) and *Mt*DXPS (see curves in Fig. S1†). (E) Top-ranked docking pose of compound 1 with *Dr*DXPS with His304 interaction. (F) Top-ranked docking pose of compound 2 with *Dr*DXPS with H304 interaction. All the docking studies in this paper were performed with the software LeadIT;^44^ Fig. 2C was generated with MOE;^45^ Fig. 2A, E and F were generated with Poseview.^46^

We defined three structurally diverse pseudo-inhibitors: deazathiamine (DZT) and fragments 1 and 2. DZT (Fig. 2A) was derived from ThDP by removing the ylide functionality and the diphosphate group. It is a weak inhibitor of *Dr*DXPS, but is not active against the target enzyme *Mt*DXPS.^37^ Despite the high structural similarity compared to ThDP, DZT shows a unique MOI on *Dr*DXPS by being competitive with ThDP, PYR and d-GAP (Fig. 2B). Docking studies suggest that while the aminopyrimidine and thiophene rings are likely to occupy the same pocket as ThDP (Fig. 2C), the hydroxyl group of DZT, whose linker is too short to reach the diphosphate anchors of ThDP, is free to move. In the top-ranked docking poses of DZT, the hydroxyl group forms a hydrogen bond with His304 (Fig. 2A). It has been reported that His304 is essential in substrate recognition, so we performed site-directed mutagenesis (SDM) to confirm the AP of DZT by mutation of His304 to alanine.^43^ The H304A mutant of *Dr*DXPS shows significant attenuation of Michaelis–Menten constants (*K*_m_) of ThDP, PYR and d-GAP, indicating that His304 plays a vital role in both cofactor binding and substrate recognition (Fig. 2D and S1†). When tested against the H304A mutant, DZT completely lost its activity (Fig. 2B), suggesting the interaction with H304 is necessary to correctly orient the aromatic pharmacophores into the hydrophobic pocket. Therefore, by directly targeting His304, DZT was validated as a promising prototype of a drug-like inhibitor against DXPS. Superimposing *Dr*DXPS (PDB code: 2O1X) with the validated homology model of *Mt*DXPS, reveals His296 as the analogous residue to His304 on *Dr*DXPS, supporting the structural basis and confirming the feasibility of the pseudo-inhibitor model (Fig. 2C).^36,38^ To enhance the structural diversity of our initial ligands for LBVS, we also included pseudo-inhibitors 1 and 2 as ligands, which are weak inhibitors against *Dr*DXPS developed by *de novo* fragment-based drug design (FBDD) and are predicted to bind to His304 in docking experiments (Fig. 2E and F).^37^

### LBVS

**Fig. 3:**
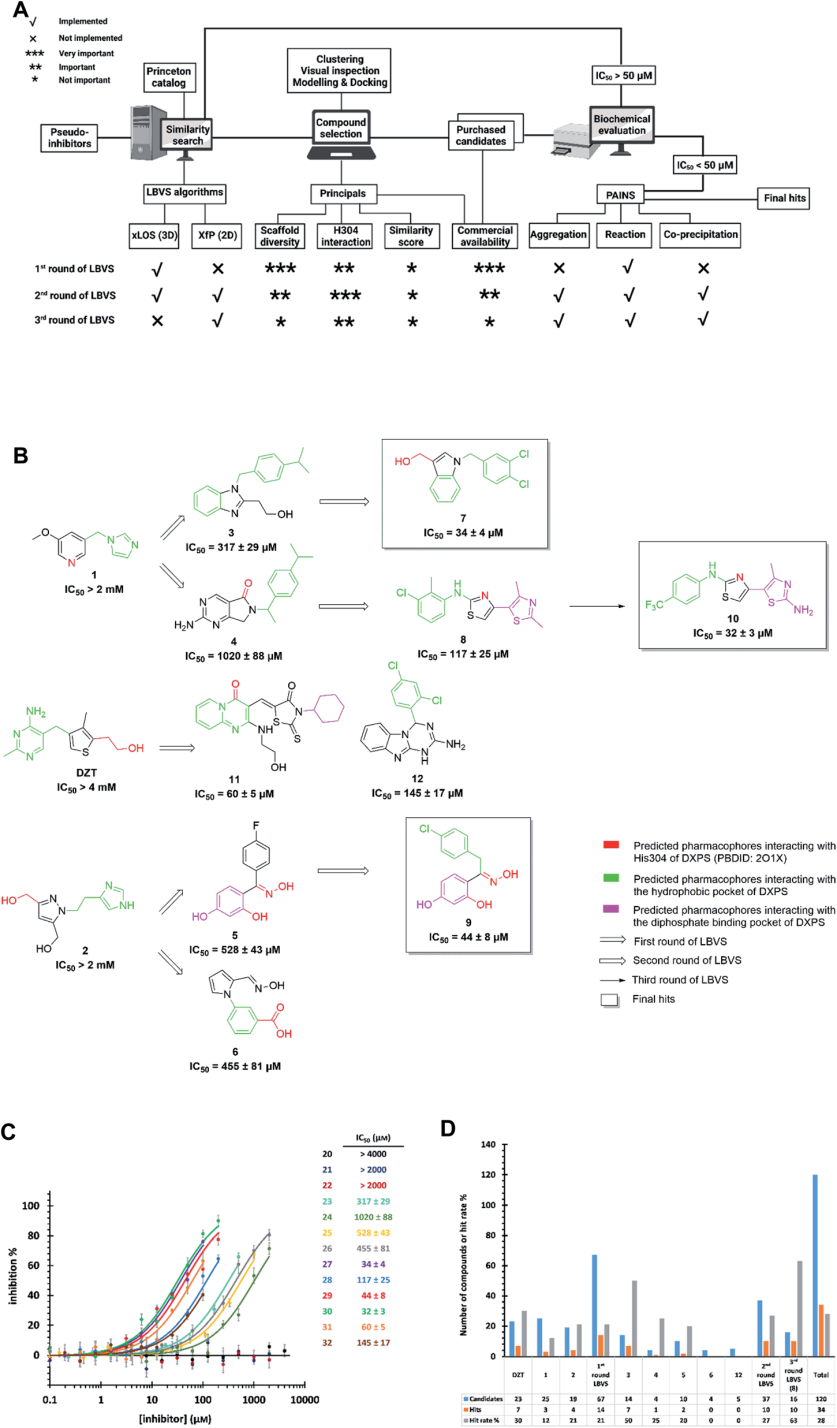
(A) Flow-chart of ligand-based virtual screening (LBVS) created with https://BioRender.com. (B) Schematic presentation of LBVS with most active hits of each round. (C) Dose–response curves of deazathiamine (DZT) and compounds 1–10 against *Mt*DXPS. Assay conditions: [ThDP] = 15 μM, [pyruvate] = 0.5 mM, [d-GAP] = 0.5 mM, [DXPS] = 1.25 μM. (D) Statistics of purchased candidates and hits.

Starting from the pseudo-inhibitors DZT, 1 and 2, we performed three rounds of LBVS against *Mt*DXPS (Fig. 3A and B). First, the 3D structures of the reference ligands were generated with CORINA.^47^ To focus on drug-like molecules and to save computer power, we restricted our search to ∼800 000 compounds from the Princeton catalogue. To calculate the structure similarity of the screening compounds, two LBVS algorithms were used. The atom-category extended Ligand Overlap Score (xLOS) is a 3D-shape and -pharmacophore matching algorithm, which is uniquely suited for scaffold hopping and has been successfully employed in hit discovery.^48–50^ xLOS was extensively used in the first round of LBVS, to search for inhibitors against *Mt*DXPS “from scratch”. As soon as the reference inhibitor showed a moderate potency against *Mt*DXPS in the second and third round of LBVS, we implemented a topological shape and pharmacophore fingerprint algorithm called extended atom pair FingerPrint (XfP).^47^ This method was developed to overcome the computational demand associated with precise 3D-shape screening of large databases and it was shown to correlate well with various representations of molecular shape extracted from 3D structures. Of note, the combination of xLOS and XfP in the second round of LBVS led to the highest hit rate.

Then, we carefully analyzed the virtual ligands on the basis of clustering, visual inspection, modeling and docking. We considered scaffold diversity as the most important factor during the first round of LBVS, as the pseudo-inhibitors are not active against *Mt*DXPS. To boost the chance of hitting the target, we wanted to cover as many scaffolds as possible (Table S1†). Later, when we performed the second and third round of LBVS from true inhibitors against *Mt*DXPS using the XfP algorithm, the resulting virtual ligands were less structurally diverse (Tables S2 and S3†). Although the His304 interaction was not mandatory in any of the LBVS rounds, all key candidates showed it in the top-ranked docking pose, indicating hits with such MOI were “enriched” *via* LBVS (Fig. 3B; see PoseView in Fig. S2†). Commercial availability was a crucial factor for the first round of LBVS to get rapid feedback without any synthetic work. In subsequent iterations of LBVS, commercial availability was no longer a limitation as we initiated the synthesis of derivatives of the hits.

After purchasing and chemical characterization, the candidates were submitted for biochemical evaluation using a coupled spectrophotometric enzyme activity assay in duplicate (Fig. 3C).^37^ When the IC_50_ values of the hits were greater than 50 μM, they would become the starting points for the next round of LBVS. For IC_50_ values smaller than 50 μM, they were first tested to exclude Pan-Assay Interference Compounds (PAINS).^51^

We purchased 67, 37 and 16 compounds for the first, second and third LBVS rounds, respectively, adding up to a total number of 120, with a hit rate of 28% well above the average hit rate of 13% for conventional VS campaigns (Fig. 3D).^52^ Even for the first round of LBVS, starting from pseudo-ligands, the hit rate was 21%, demonstrating the power of the xLOS algorithm in identifying novel hit scaffolds. In addition, most of the LBVS candidates were small, hydrophobic and drug-like molecules, with a mean value of 286 Da overall and 304 Da for hits (Fig. S3A†), and a cLogP of 2.78 overall and 3.23 for hits (Fig. S3B†), demonstrating the feasibility and efficiency of standard CADD tools in antibiotic drug discovery.

The best hits of each LBVS round are presented in Fig. 3B. The first round of LBVS led to moderate inhibitors of *Mt*DXPS (hits 3–6, 11 and 12). All six hits served as a reference for the second round of LBVS providing hits 7–9. As there was a rich source of derivatives of 8 available, we performed the third round of LBVS starting from 8 and achieved hit 10. We did not further screen based on 7 and 9 because they already have promising activity. Compounds 6 and 12 were discontinued because they could not be improved in the next round of screening and compound 11 is a reactive false positive as it also inhibits several other related or unrelated enzymes. Its inhibition could be significantly attenuated by increasing the dithiothreitol (DTT) concentration (Fig. S4A and S4B†).^53^ In summary, DZT failed to afford validated hits, indicating the importance of including diverse reference structures at the beginning. The most promising hits 7, 9 and 10, derived from compounds 1 and 2, were evaluated with dynamic light scattering (DLS), DTT dependency and in centrifugation experiments to exclude the possibilities of aggregation, reactive false positives and co-precipitation, respectively (Fig. S5A–C†).^53,54^ In summary, our iterative LBVS campaign afforded indole 7, oxime 9 and aminothiazole 10 as final hits, representing three structurally diverse, drug-like scaffolds, with promising *in vitro* activity against *Mt*DXPS.

### Characterization of compounds 7, 9 and 10 against *Mt*DXPS

**Fig. 4:**
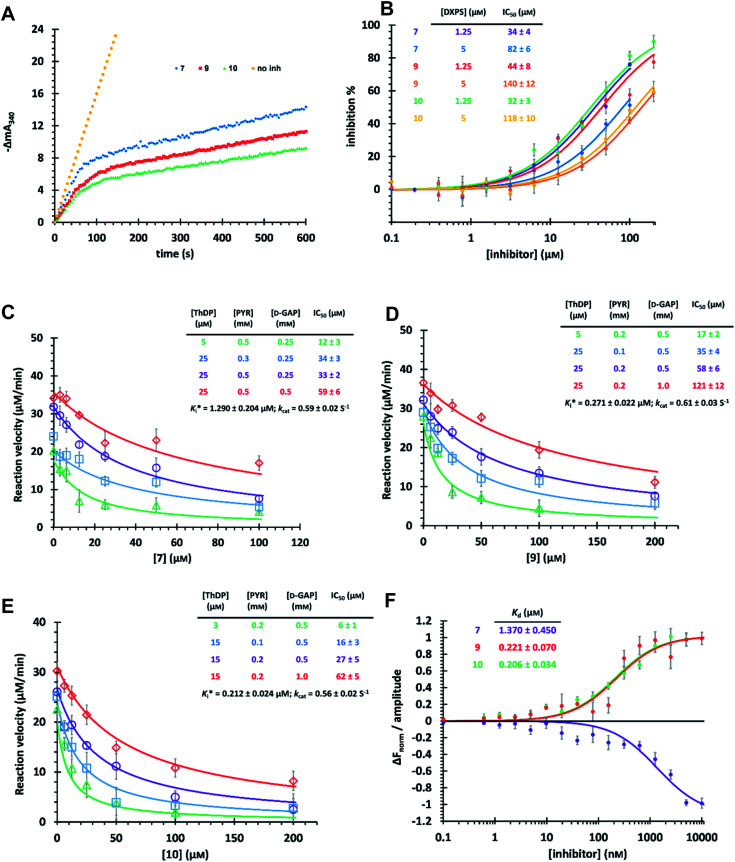
(A) Time-dependent progress curves of the final hits. Conditions can be found in ESI Methods 3.2.7.† (B) Dose–response curves of the final hits by varying *Mt*DXPS concentration. (C), (D) and (E) Mode-of-inhibition study and determination of *K*_i_* of compounds 7, 9 and 10, respectively. Color code: reference condition: purple; varying [ThDP]: green; varying [PYR]: blue; varying [d-GAP]: red. (B)–(E) The reaction velocity was measured after 200 s, when the slow-onset phase was considered complete. (F) Determination of *K*_D_ of the final hits with microscale thermophoresis (MST) (binding curves). Curve shape is dependent on molecular movement during measurement and has no influence on *K*_D_ determination.

The time-dependent progress curves indicate that the final hits 7, 9 and 10 inhibit *Mt*DXPS in a slow-binding manner, with an equilibration time of ∼100 s (Fig. 4A). Increasing DXPS concentration leads to a drop of inhibitory potency of 7, 9 and 10, which points to a tight-binding pattern (Fig. 4B). In addition, MOI studies show that compound 7 is competitive with ThDP and d-GAP, but non-competitive with PYR (Fig. 4C); while compounds 9 and 10 were competitive with ThDP and both substrates (Fig. 4D and E). As the conventional Cheng–Prusoff model is not suitable to characterize slow, tight-binding inhibitors, we implemented a tight-binding Morrison model based on the random sequential mechanism of DXPS.^55,56^ Slow, tight-binding inhibition is most commonly attributed to one of two binding models: (1) one-step binding: *E* + *I* = *E*·*I* or (2) two-step binding: *E* + *I* = *E*·*I* = E*·*I*. The hyperbolic plot of the apparent catalytic constant *k*_obs_ against the inhibitor concentrations suggests the two-step Morrison model is appropriate (Fig. S6†). By fitting the dose-dependent curves into the Morrison model with the software Dynafit as done in the past, we determined the Morrison constants (*K*_i_*) of 7, 9 and 10 to be 1.29 μM, 0.271 μM and 0.212 μM, respectively.^57–59^ Direct measurement of dissociation constants (*K*_D_) with microscale thermophoresis (MST) further validated the accuracy of the Morrison model, with *K*_D_ for 7, 9 and 10 to be 1.370 μM, 0.221 μM and 0.206 μM, respectively (Fig. 4F and S7†). Inspiringly, compounds 9 and 10 were the first drug-like inhibitors with sub-micromolar inhibition potency against any DXPS homologue. In contrast, other potent DXPS inhibitors are not cell-permeable and need a prodrug approach to enter cells.^60^ The final hits also revealed promising selectivity by being inactive against the ThDP-dependent mammalian pyruvate dehydrogenase (PDH) (Fig. S8†).

### Pharmacophore validation on *Dr*DXPS

As *Mt*DXPS was 15-fold less active (Fig. 2E) and more unstable than *Dr*DXPS, performing an SDM study directly on *Mt*DXPS was challenging. However, the success of pseudo-inhibitor-based LBVS and the weak activity against *Dr*DXPS suggest that *Dr*DXPS is a suitable model for *Mt*DXPS for the pharmacophore validation on *Dr*DXPS.

**Fig. 5:**
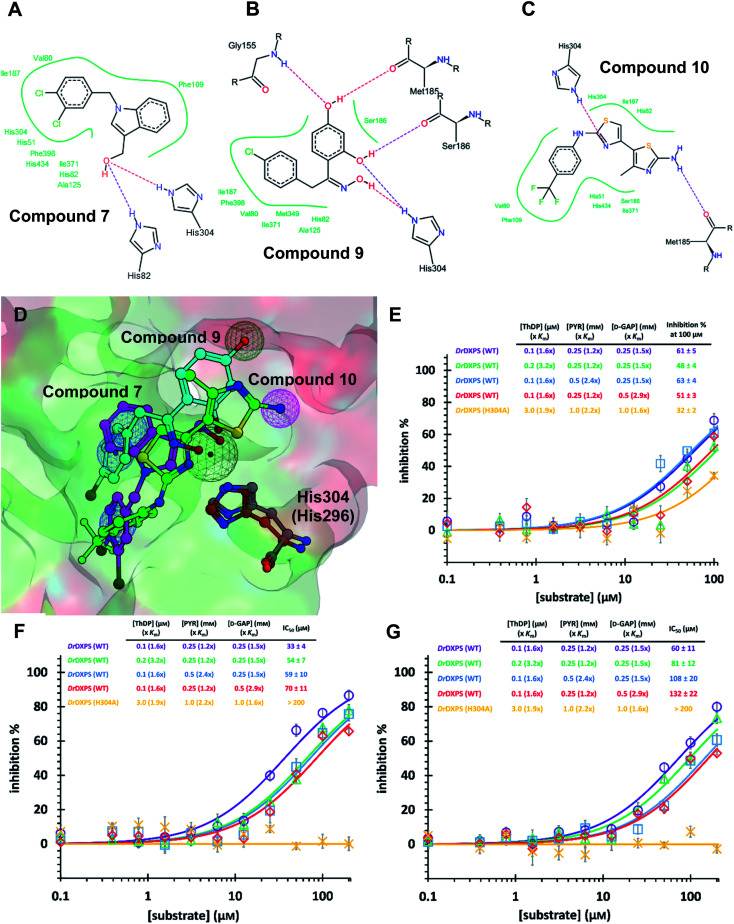
(A) (B) (C) Poseview of compounds 7, 9 and 10. (D) Superimposed docked pose of the final hits into *Dr*DXPS with pharmacophore view. Color code: C skeleton: 7: magenta; 9: light blue; 10: green; His304 (*Dr*DXPS): gray; His296 (*Mt*DXPS): brown. Surface: hydrophobic site: green; hydrophilic site: red. (E), (F) and (G) Mode-of-inhibition and site-directed mutagenesis studies of the final hits against *Dr*DXPS. Fig. 5D was generated with MOE;^45^ Fig. 5A–C were generated with Poseview.^46^

For this, we used our previously created His304 mutant, in which His304 is replaced by an alanine. Based on the docking results (Fig. 5A–D), all compounds interact with His304 and should therefore, not be able to inhibit the mutant. This hypothesis was confirmed for compounds 9 and 10 that are both inactive (Fig. 5F and G), but compound 7 (Fig. 5E) retained some activity. A closer look at the docking pose of compound 7 explains this phenomenon as the hydroxyl group of 7 also interacts with His82, which has been shown to be involved in catalysis in the wild type and without His304 present, might take over this role in the mutant.^43^

### Pharmacophore validation on *Mt*DXPS

Pharmacophore validation on *Mt*DXPS was mainly based on the analysis of structure–activity relationships (SARs). We designed and synthesized a series of derivatives of compounds 7 and 9 according to docking results and the Topliss scheme, as there were no commercially available derivatives of the two hits.^61^ For compound 10, SARs were derived from the third round of LBVS. Their inhibitory activities were calculated as *K*_i_* against *Mt*DXPS (Table 1A–C and S9A–C†). We focused on three moieties: R^0^, the key pharmacophore that interacts with His304 (His296 in *Mt*DXPS) and positions the inhibitor in the right orientation, *i.e.* the hydroxyl group of compound 7, the oxime group of compound 9, and the nitrogen atom of the thiazolyl ring of compound 10; ring 1, the methylene- or imino-linked aromatic groups that grow into the hydrophobic pocket lined by phenylalanine and histidine residues, possibly contributing to π–π or halogen–π interactions; ring 2, the aromatic rings that flip towards the opening of the diphosphate binding pocket (Fig. 5D). The results indicate that R^0^ is an essential anchor for all derivatives, but inhibition can be lost if ring 1 is not electron-deficient. Compounds 15, 16 and 22 still contain a hydroxyl or oxime group, but R^1^ is not sufficiently electron-withdrawing and activity is lost. If R^0^ is replaced with a different group, as in derivatives 13, 14 and 20, activity is lost as well, but the oxime hydroxyl group can be replaced with an amino group (compound 21) and activity is partially retained. Modifications on ring 2 lead to a moderate increase or decrease of activity. Compounds 17 and 25 are more active than their parent molecules 7 and 9 due to different substituents on ring 2. Derivatives of compound 10 showed the same trends. Ring 1 has to be electron-deficient, otherwise activity is lost (compound 26), while different R^2^ groups are tolerated in most cases. For the aminothiazole class, no improved hit could be found, but the parent compound 10 is a potent inhibitor already. We could demonstrate that R^0^ is essential for binding to *Mt*DXPS as predicted and that strategic planning can reduce the synthetic effort significantly while still giving important insights into the SAR.

(A), (B) and (C) *K*_i_* and structures of the derivatives of the final hits

**Table:** 

A 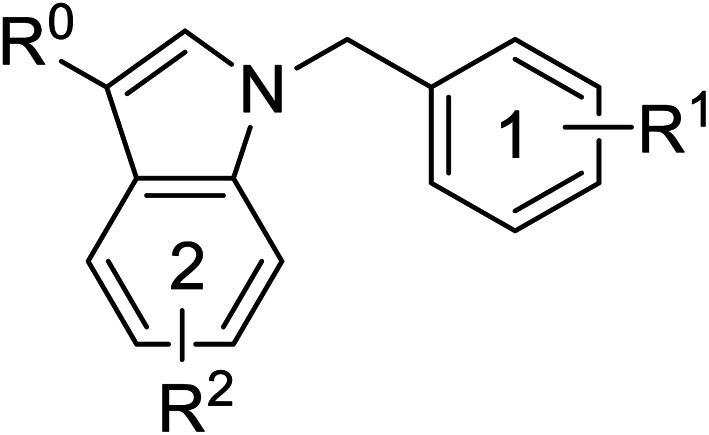					
Cmpd no.	R^0^	R^1^		R^2^	*K*_i_* (μM)
		*meta*	*para*		
7	–CH_2_OH	–Cl	–Cl	—	1.3 ± 0.2
13	–CHO	–Cl	–Cl	—	>10
14	–CH_2_OCH_3_	–Cl	–Cl	—	>5
15	–CH_2_OH	–Cl	–H	—	>10
16	–CH_2_OH	–H	–Cl	—	5.5 ± 0.6
17	–CH_2_OH	–Cl	–Cl	5-F	0.4 ± 0.06
18	–CH_2_OH	–Cl	–Cl	6-CN	1.7 ± 0.3
19	–CH_2_OH	–Cl	–Cl	7-OCH_3_	1.4 ± 0.3

**Table:** 

B 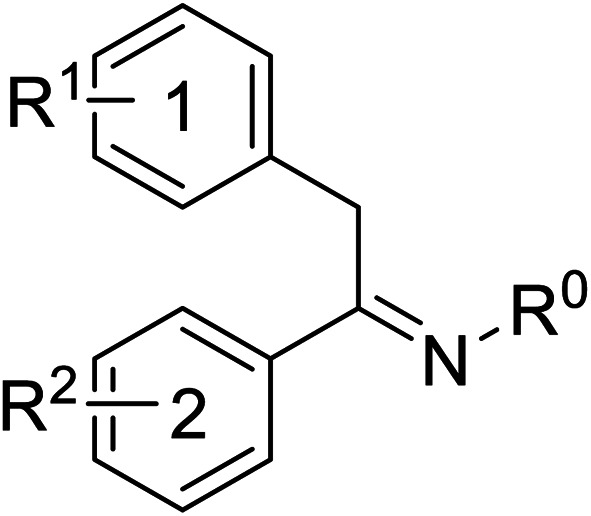					
Cmpd no.	R^0^	R^1^	R^2^		*K*_i_* (μM)
			*ortho*	*para*	
9	–OH	4-Cl	–OH	–OH	0.3 ± 0.02
20	–OCH_3_	4-Cl	–OH	–OH	>10
21	–NH_2_	4-Cl	–OH	–OH	0.4 ± 0.05
22	–OH	4-OCH_3_	–OH	–OH	>10
23	–OH	4-Cl	–H	–H	1.1 ± 0.2
24	–OH	4-Cl	–OH	–OCH_3_	0.5 ± 0.1
25	–OH	4-Cl	–OH	–NH_2_	0.1 ± 0.03

**Table:** 

C 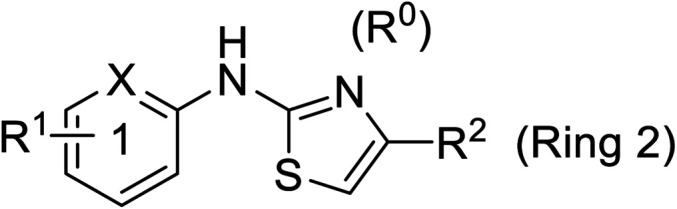				
Cmpd no.	X	R^1^	R^2^	*K*_i_* (μM)
10	–CH	4-CF_3_	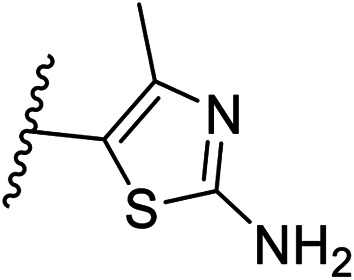	0.2 ± 0.02
26	–CH	–H	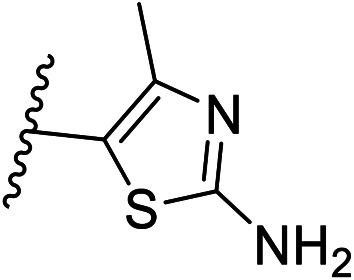	>10
27	–CH	2-CF_3_	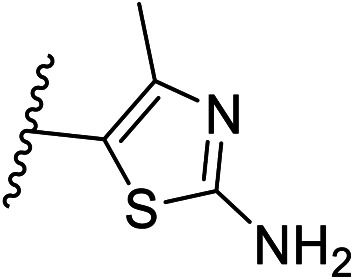	0.3 ± 0.02
28	–CH	3-CF_3_	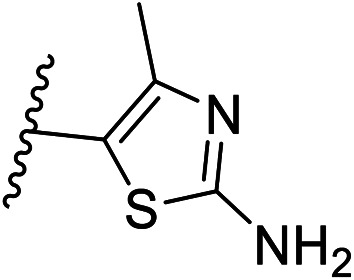	0.4 ± 0.06
29	–CH	3-CF_3_	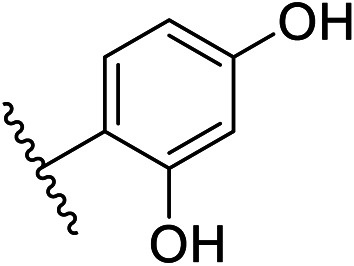	0.5 ± 0.04
30	–N	4-Cl	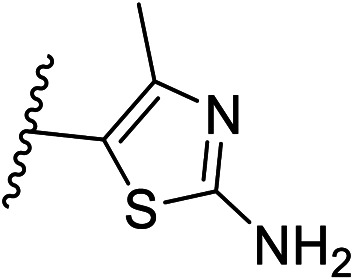	0.3 ± 0.1
31	–N	4-Cl	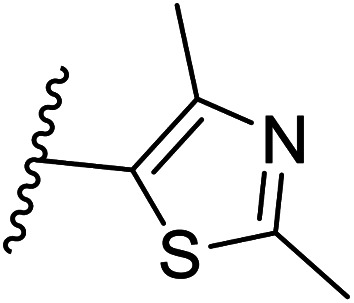	>10

### Intracellular target validation, cell-based antitubercular activity and ADMET study

**Fig. 6:**
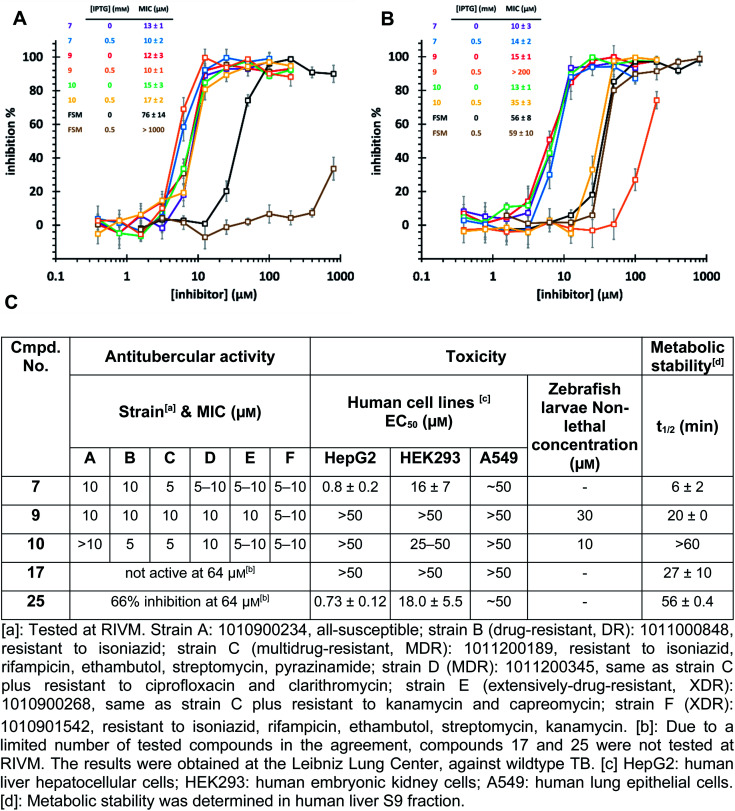
(A) Minimum inhibitory concentration (MIC) determination on *E. coli* strain BL21 (DE3) pQEYAEM. The overexpression of *E. coli* IspC triggered by isopropyl-β-d-thiogalactopyranosid (IPTG) leads to a significant increase in MIC of fosmidomycin, but has little effect on hits 7, 9 and 10. (B) MIC determination on *E. coli* strain BL21 (DE3) pET22b-H6TEVEKMTDXS. The overexpression of *Mt*DXPS triggered by IPTG leads to significant attenuation of inhibitory activity of compounds 9 and 10, but has little effect on compound 7 and fosmidomycin. (C) Cell-based antitubercular activity of compounds 7, 9, 10, 17 and 25, and their ADMET study results.

The high biosafety level and slow growth of *M. tuberculosis* strains complicate target-validation studies. Therefore, we used *E. coli* (BL21(DE3)) as a model strain for target validation, as our hits are also active on *E. coli* (*Ec*)DXPS (Fig. S10†). To check if *E. coli* is suitable for target validation by overexpression of MEP pathway enzymes, we used the nanomolar inhibitor of *Ec*IspC fosmidomycin in an *E. coli* strain carrying an IspC-expressing plasmid. We observed attenuation of inhibition by fosmidomycin by adding isopropyl β-d-1-thiogalactopyranoside (IPTG) to trigger the IspC overexpression, while our hits showed unchanged MIC values, making *E. coli* a suitable model for target validation (Fig. 6A). In turn, for the *E. coli* strain carrying *Mt*DXPS-expressing plasmids, adding IPTG leads to a significant increase of MIC values for compounds 9 and 10, but shows little effect on compound 7 and fosmidomycin (Fig. 6B). The results suggest *Mt*DXPS could be the intracellular target of 9 and 10, but not the main target of compound 7.

Our LBVS hits show promising antitubercular activities (5–10 μM) against multiple strains of *M. tuberculosis*, including drug-resistant (DR), multidrug-resistant (MDR) and extensively-drug-resistant (XDR) strains (Fig. 6C). The improved indole derivative 17, however, shows no activity against *M. tuberculosis* strains and oxime 25 retains some activity, which can have different reasons such as additional targets or a different uptake behavior in *M. tuberculosis* and has to be investigated further.

An *in vitro* ADMET study indicated toxicity against human cell lines and a short half-life of 7, but compounds 9 and 10 display promising ADMET properties of lead quality. Compared with sub-micromolar activities on the target DXPS and low micromolar MIC values against *M. tuberculosis* strains, hits 9 and 10 have a relatively wide safety window in human cell lines and zebrafish larvae embryos (Fig. 6C and Table S4†). Furthermore, their metabolic half-lives are acceptable (Fig. 6C and S11†). In addition, compound 10 displays a lower frequency-of-resistance development than clinically used antibiotics (Tables S5 and S6†). With these encouraging properties, the hit scaffolds 9 and 10 could be selected as early drug leads to develop antitubercular agents with a novel MOI, fighting against AMR.

## Conclusions

In the midst of the current AMR crisis where current research efforts are struggling to fill the antibiotic pipeline, we show that a thorough study of the challenges and risks paired with strategic steps, makes the use of computational tools on difficult antibiotic targets as smooth as on conventional targets as exemplified by DXPS. We found that: (i) targeting the catalytic center under careful consideration of known parameters is essential. For antimicrobial targets with large, hydrophilic binding sites, tight natural binders and complex catalytic mechanisms, locating and focusing on the key amino acids involved in key interactions boosts the efficacy of hit identification. (ii) Pseudo-inhibitors are effective starting points to initiate LBVS. Introducing the concept of pseudo-inhibitors has helped us work with a target with few known inhibitors. The selection of versatile structures ensured successful LBVS rounds. (iii) Structure-based methods successfully support LBVS. Pharmacophore validation of the initial ligands (here pseudo-inhibitors) provided guidance for LBVS and implementing modeling and docking to pre-order candidates helped to “enrich” the hits with the desired inhibition pattern. In addition, we confirmed that *in vitro* inhibitory potency of inhibitors should be evaluated correctly. For the antibiotic agents interfering with the behavior of multiple reaction components of the target, IC_50_ values may not truly reflect their inhibition activity. Inhibition or dissociation constants are preferred.

By strategic application of LBVS on the challenging target DXPS, we achieved small-molecule, drug-like antibiotic agents 9 and 10 as slow-, tight-binding inhibitors competitive with both cofactor and substrates, with submicromolar activity (*K*_i_* = 0.2–0.3 μM, *K*_D_ = 0.2 μM) against *Mt*DXPS, which are the most potent drug-like inhibitors against any DXPS homologue to date. Compounds 9 and 10 are selective over mammalian ThDP-dependent enzymes. SDM and SAR studies indicate that the H-bonding with His304 (His296) and hydrophobic interactions in the histidine-rich pocket are essential for the inhibitory activities. Meanwhile, compounds 9 and 10 display promising cell-based antitubercular activity (MIC 5–10 μM) against multiple susceptible and resistant strains. We confirmed DXPS as the intracellular target in *E. coli* and hope to do the same in *M. tuberculosis* in the future. The promising antitubercular activity and ADMET profile are encouraging for hit-to-lead optimization. They are the first antitubercular, drug-like agents targeting the MEP pathway discovered by CADD. Our approach should find application to other challenging targets.

## Data availability

The datasets supporting this article have been uploaded as part of the ESI† material.

## Author contributions

D. Z.: conceptualization, methodology, validation, formal analysis, investigation, writing, visualization. S. J.: methodology, validation, formal analysis, investigation, writing, visualization. T. M., C. S.: conceptualization, methodology, software, formal analysis, investigation. J. H., B. I., A. A., R. M. G., T. v. d. L., E. B., N. R.: formal analysis, investigation. M. A.: software, formal analysis. R. v. d. V., R. N., M. P.: investigation. R. M., M. F.: resources, supervision. J.-L. R., A. K. H. H.: resources, supervision, project administration, funding acquisition. All authors: writing – review & editing.

## Conflicts of interest

The authors declare no competing financial interest.

## Supplementary Material

SC-013-D2SC02371G-s001

SC-013-D2SC02371G-s002

SC-013-D2SC02371G-s003

SC-013-D2SC02371G-s004
