# Supplementary material for: Discovery of novel drug-like antitubercular hits targeting the MEP pathway enzyme DXPS by strategic application of ligand-based virtual screening
Source: Chem Sci. 2022 Aug 8;13(36):10686–98. doi: 10.1039/d2sc02371g (PMC9491098; doi:10.1039/d2sc02371g)
Supplement: SC-013-D2SC02371G-s001 [file SC-013-D2SC02371G-s001.pdf]

Electronic Supplementary Material (ESI) for Chemical Science.
This journal is © The Royal Society of Chemistry 2022

Supplementary Figures

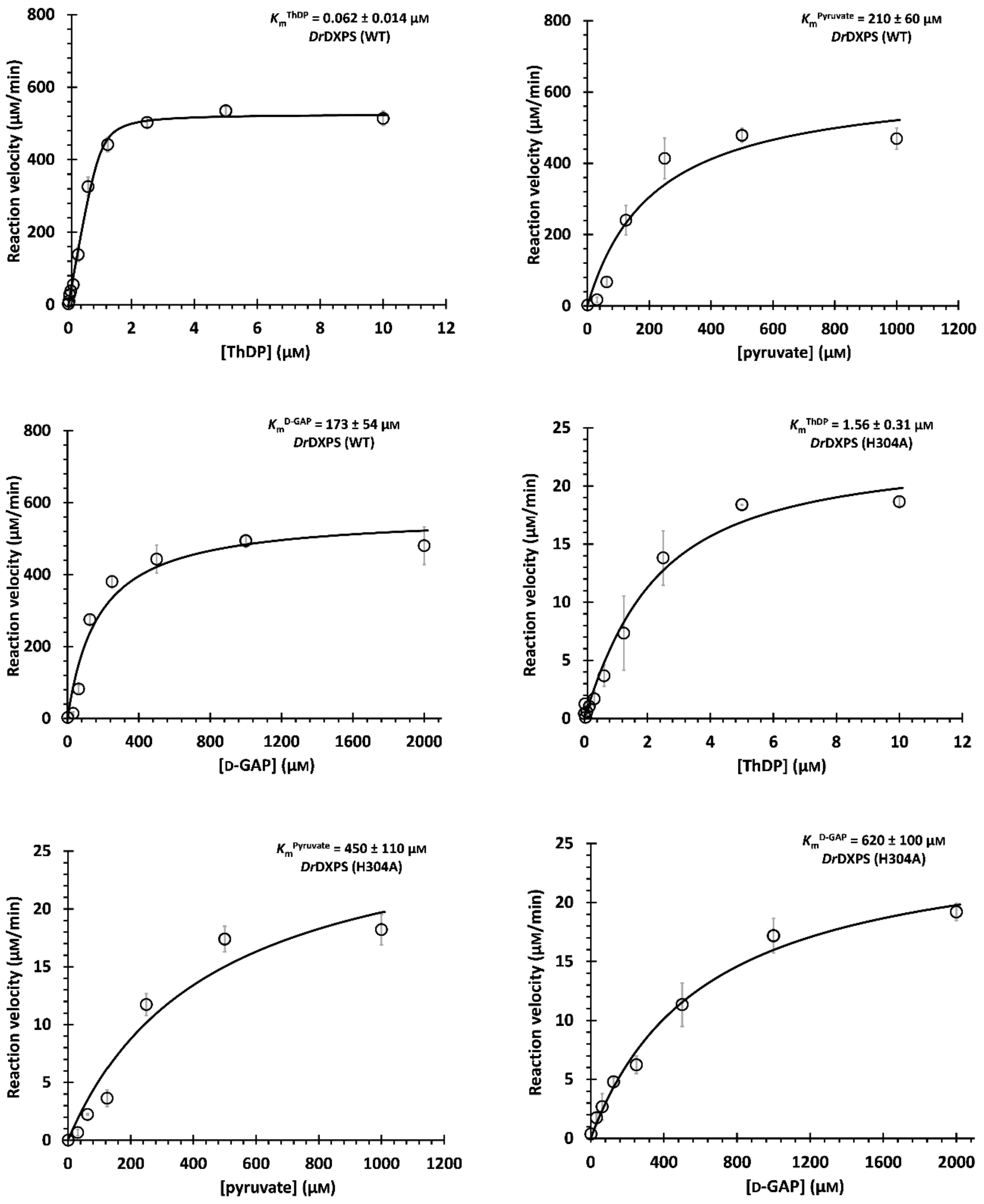

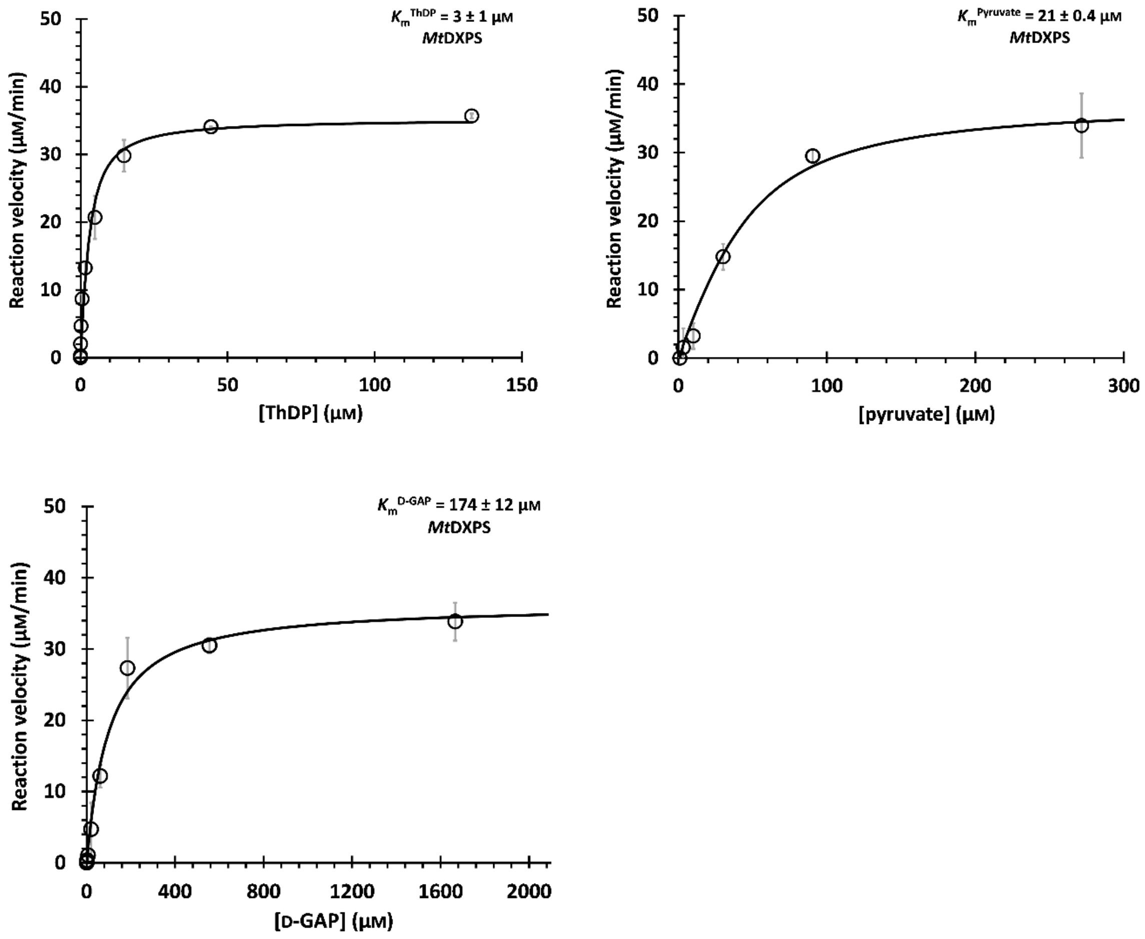

Figure S1: Kinetic characterization of *Dr*DXPS (WT), *Dr*DXPS (H304A) and *Mt*DXPS.

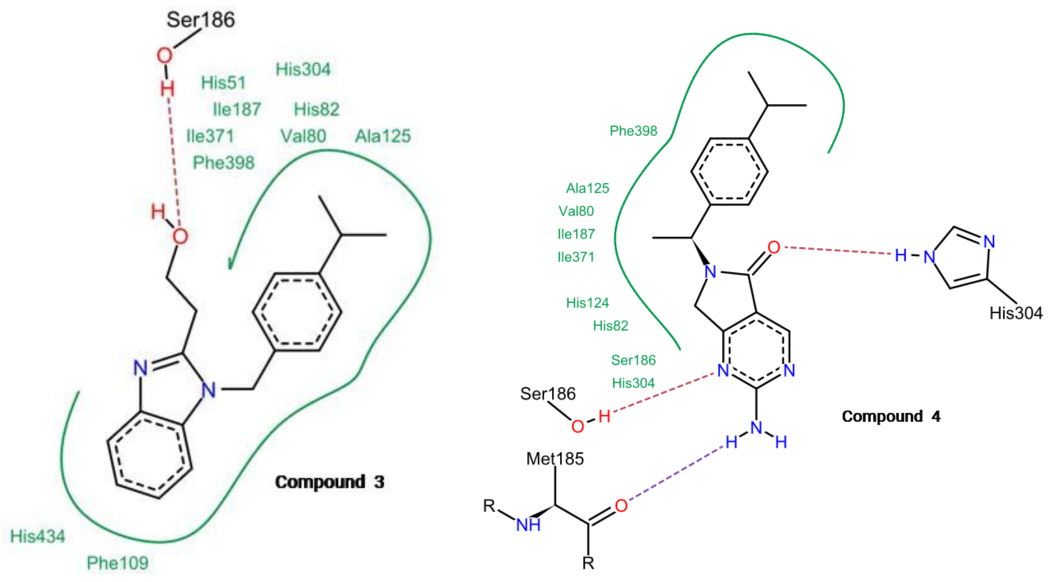

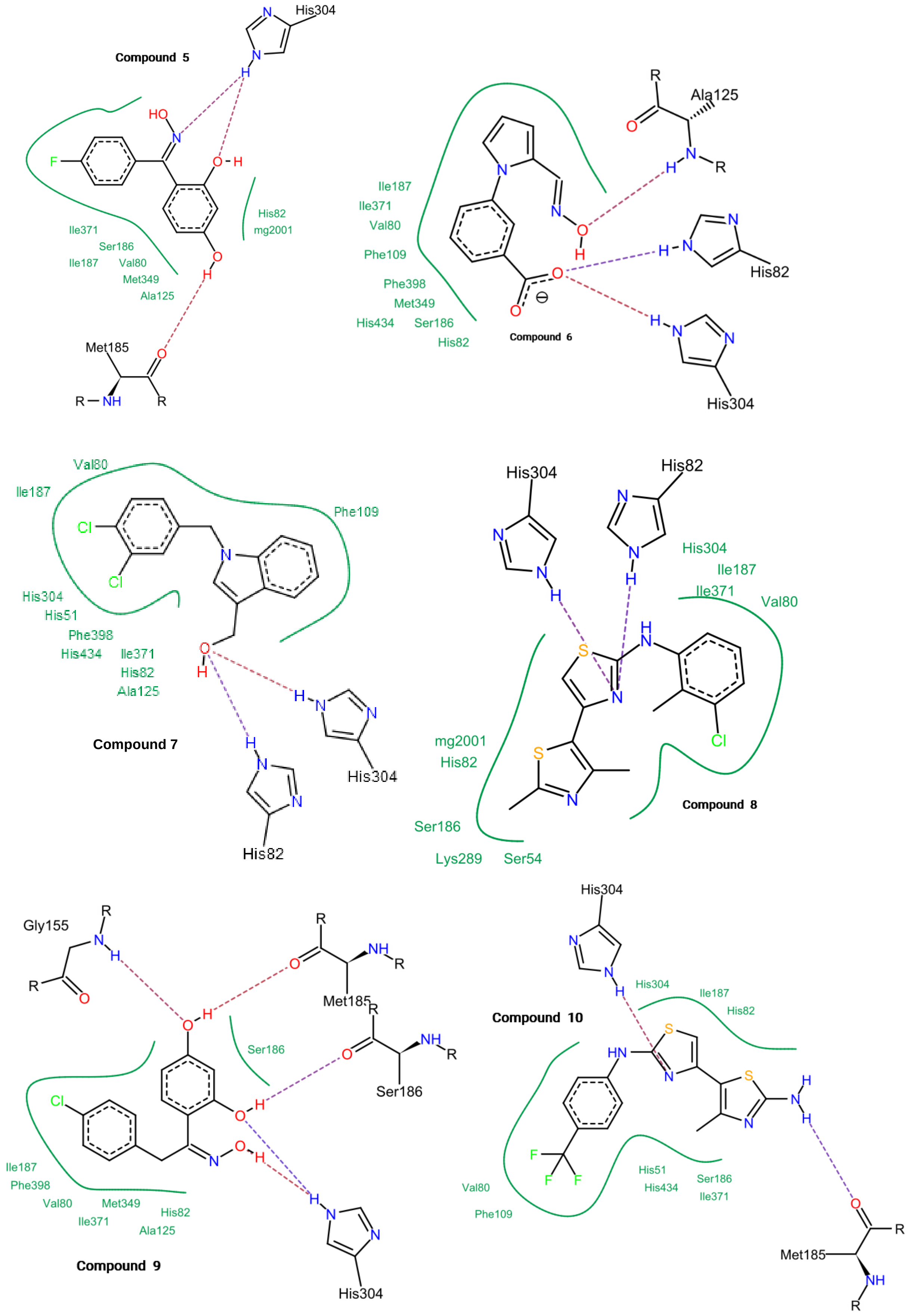

Figure S2: Top-ranked docking pose of compounds **3**–**10**.

A

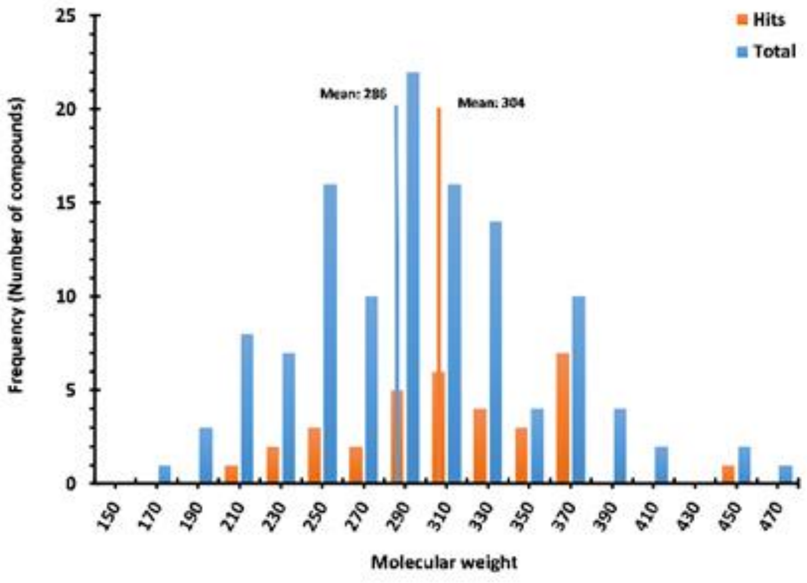

B

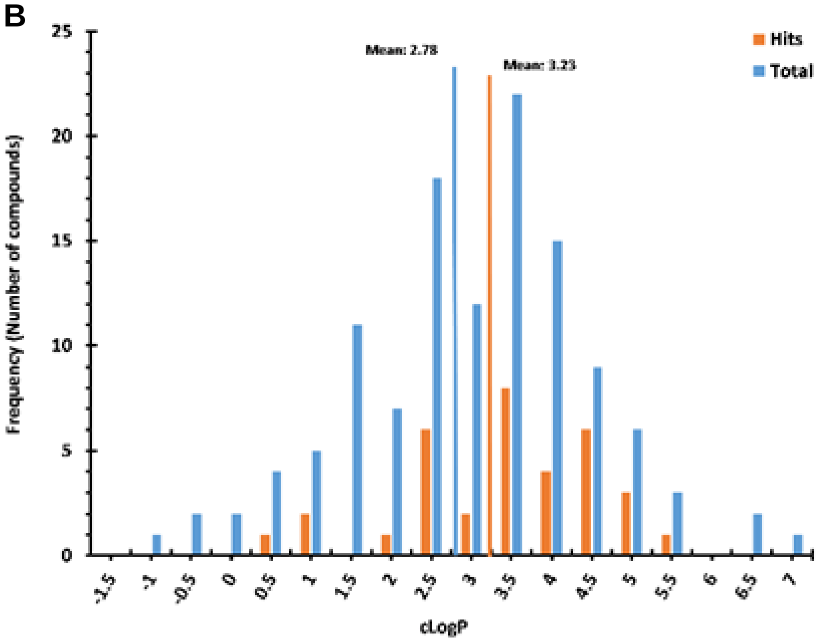

Figure S3: (**A**) Distribution of molecular weight of purchased candidates and hits. (**B**) Distribution of cLogP of purchased candidates and hits.

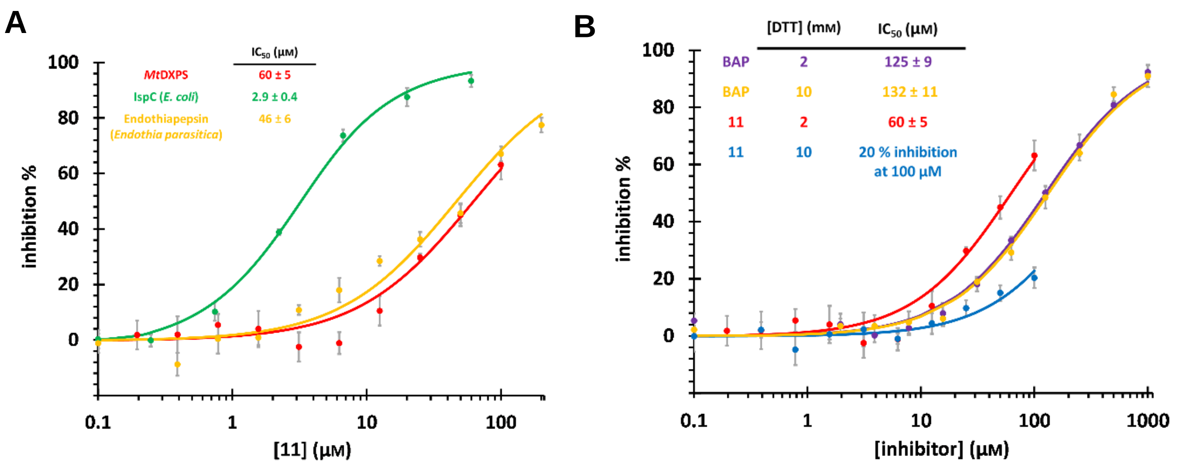

Figure S4: (**A**) Non-selective inhibition of **11** and (**B**) the effect of DTT concentration on the activity of **11**. Compound **11** was proved to be a reactive false positive against *Mt*DXPS.

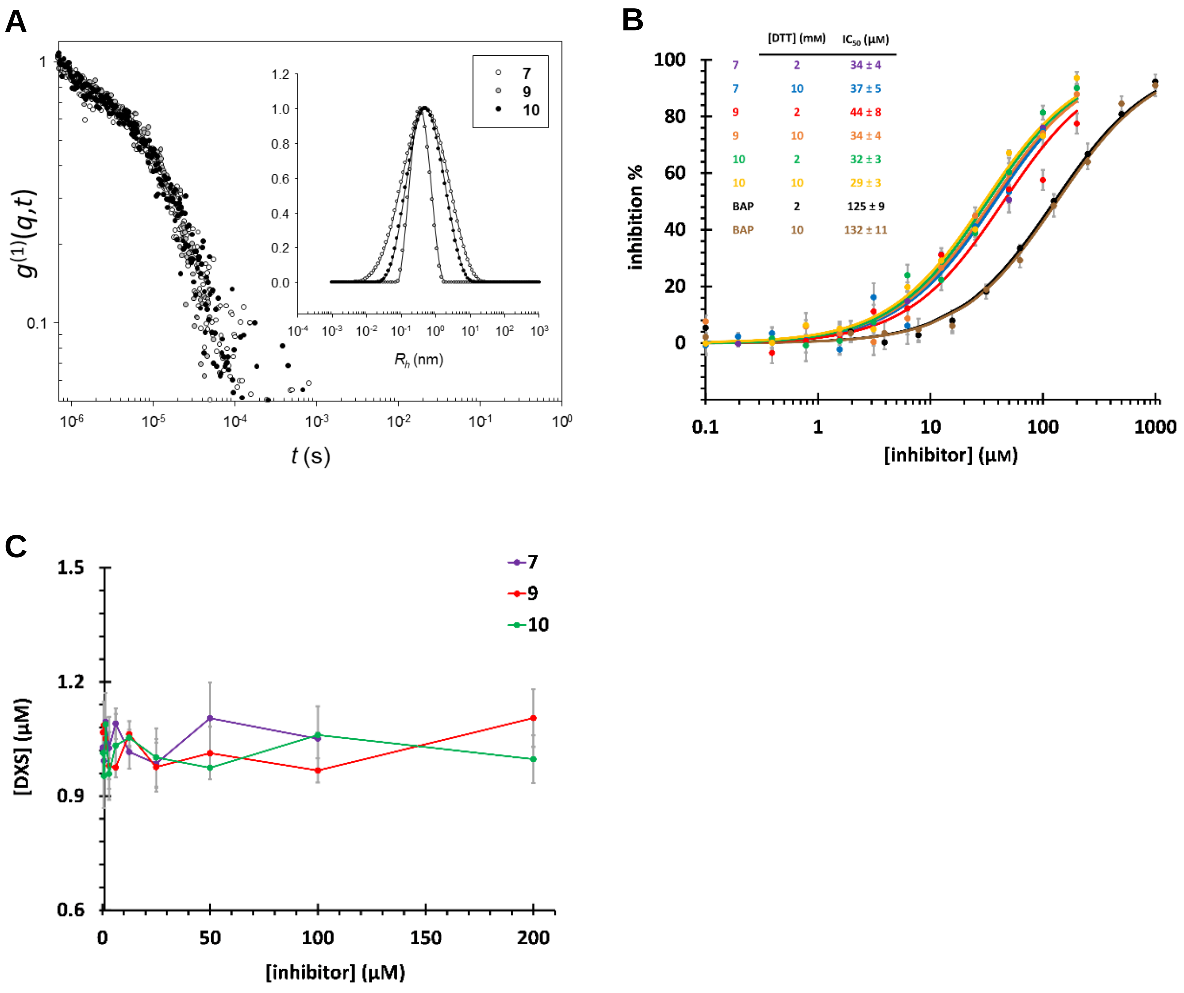

Figure S5: Hit validation of compounds **7**, **9** and **10** by (**A**) DLS, (**B**) DTT dependency and (**C**) DXPS co-precipitation. The hits were validated as true inhibitors against *Mt*DXPS, not aggregators, reactive false positives or co-precipitators.

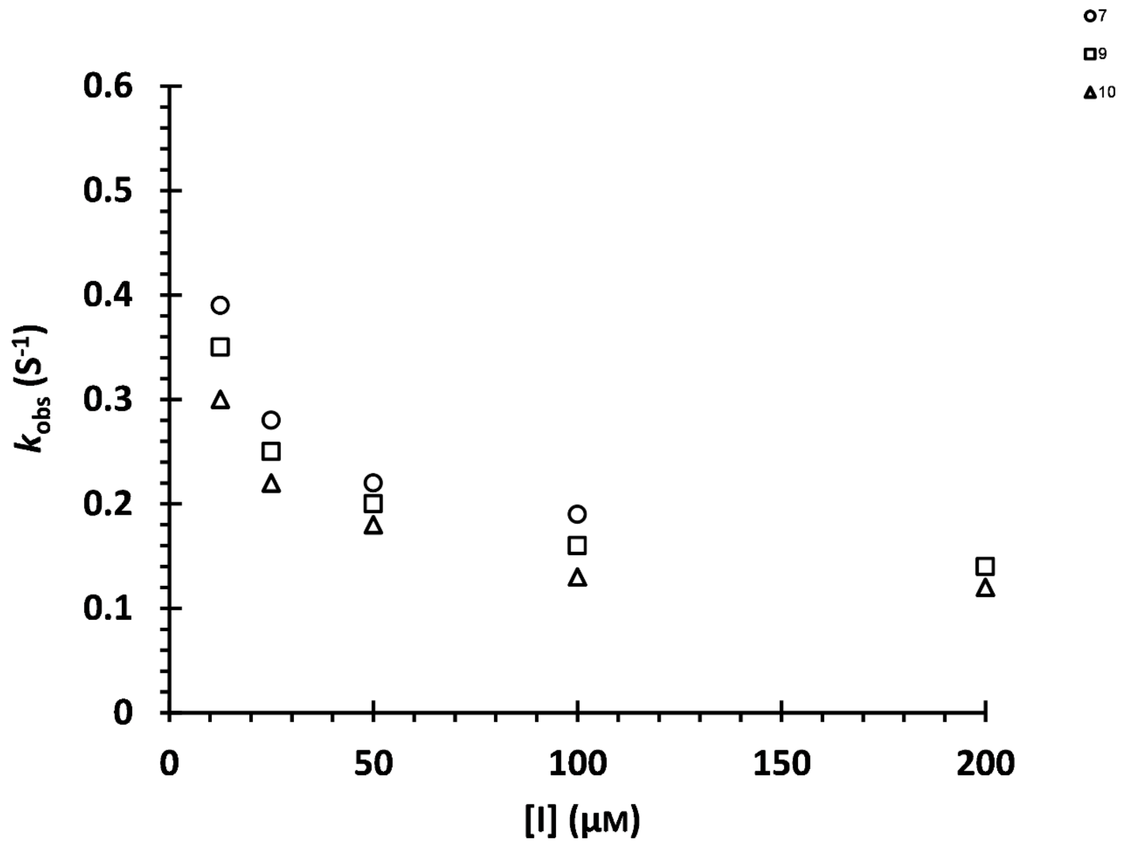

Figure S6: The hyperbolic curves for compounds **7**, **9** and **10** when comparing inhibitor concentration to k_{obs} confirms that the two-step Morrison model is appropriate to characterize the inhibitors' activity of the inhibitors against *Mt*DXPS.

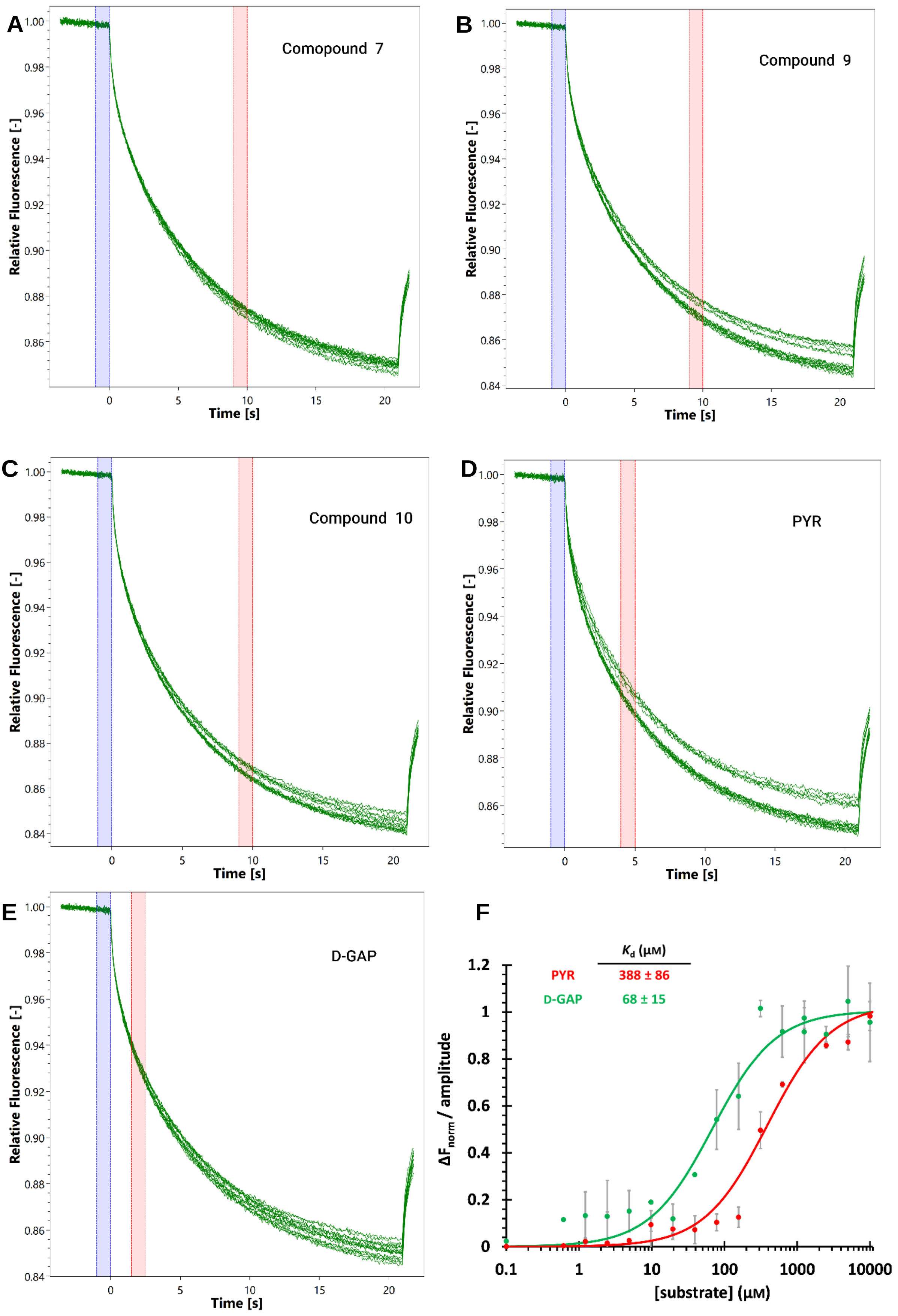

Figure S7: Raw data of MST analysis of the interaction between *Mt*DXPS and ligands: Compounds (**A**) **7**, (**B**) **9** and (**C**) **10**, and substrates (**D**) pyruvate and (**E**) D-GAP. The blue and red squares represent the MST off and on time as used to extract the binding curves. (**F**) Binding curves of pyruvate and D-GAP with *Mt*DXPS.

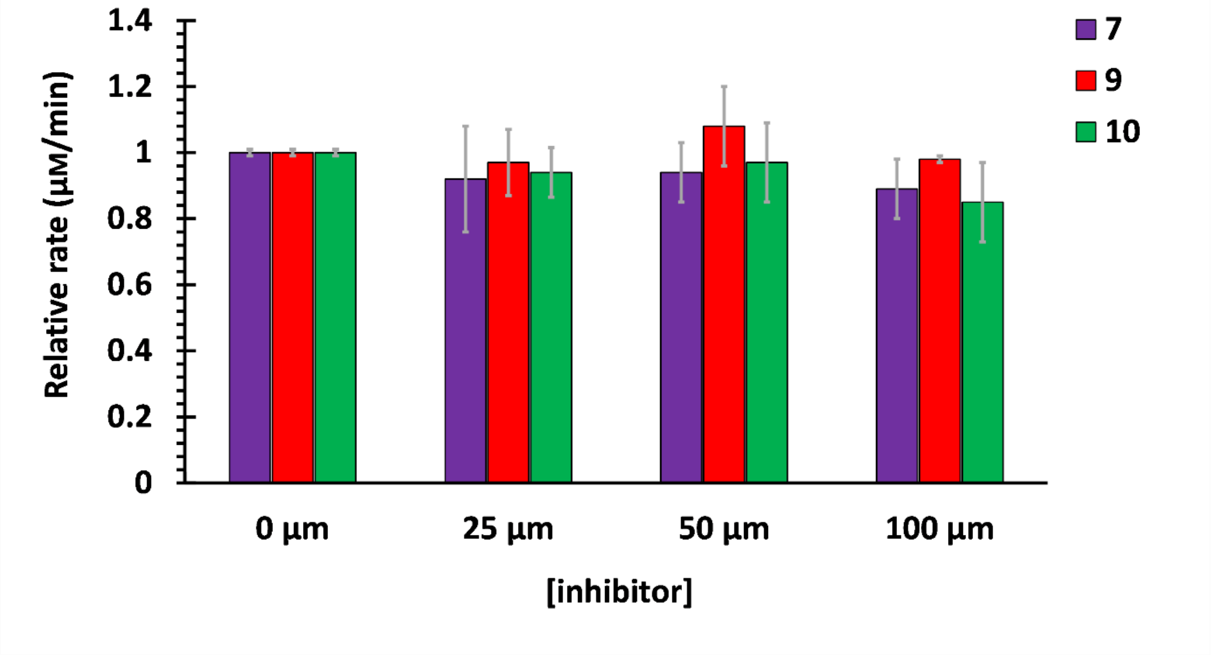

Figure S8: Dose-response bar-chart of the final hits against porcine PDH.

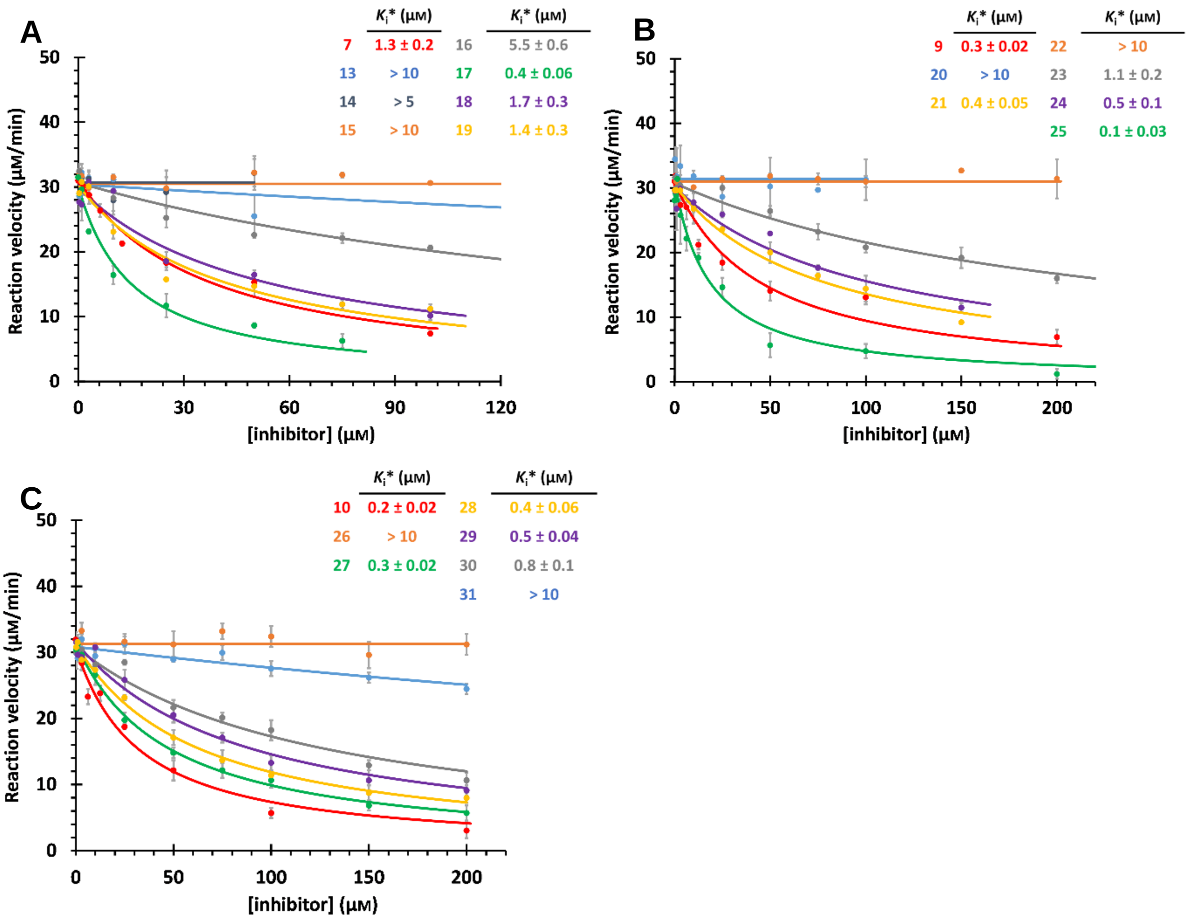

Figure S9: (**A**) (**B**) (**C**) Dose-response curves of the final hit derivatives used for K_i* determination.

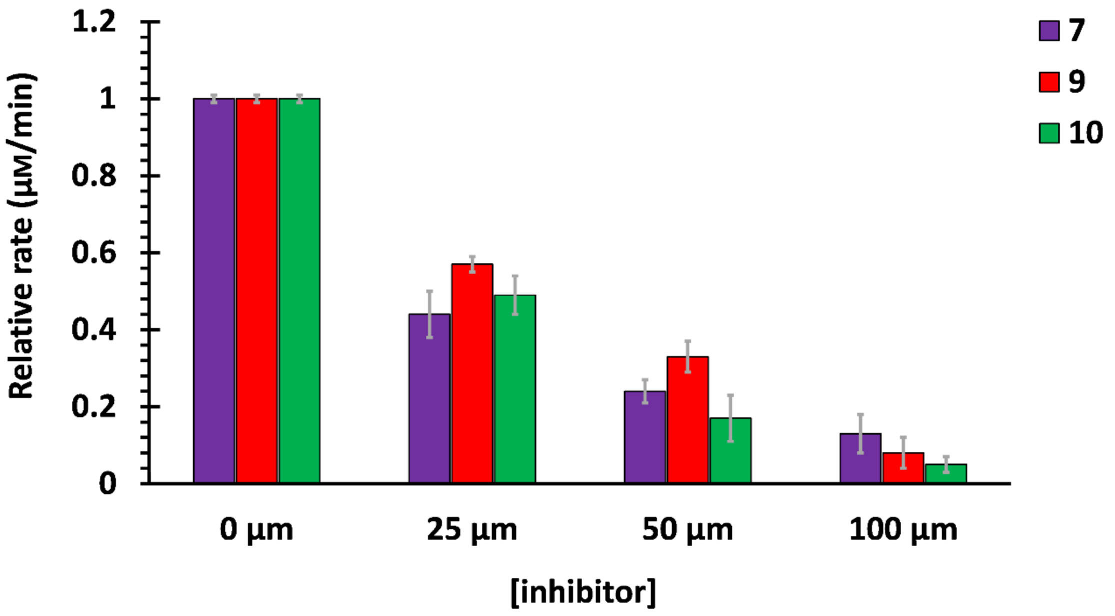

Figure S10: Dose-response bar-chart of compounds **7**, **9** and **10** against *E. coli* DXPS.

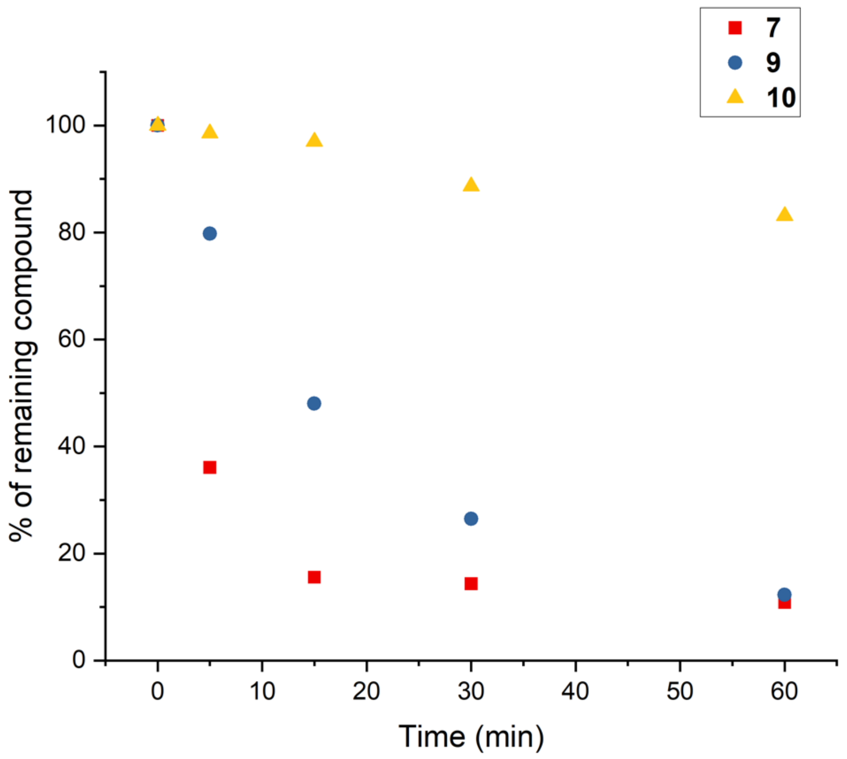

Figure S11: Metabolic stability of **7**, **9** and **10** in human liver S9 fraction. The residual percentage of the initial compound concentration is shown at different time points. Values are means of two independent determinations.
