# Supplementary material for: Discovery of novel drug-like antitubercular hits targeting the MEP pathway enzyme DXPS by strategic application of ligand-based virtual screening
Source: Chem Sci. 2022 Aug 8;13(36):10686–98. doi: 10.1039/d2sc02371g (PMC9491098; doi:10.1039/d2sc02371g)
Supplement: SC-013-D2SC02371G-s002 [file SC-013-D2SC02371G-s002.pdf]

Electronic Supplementary Material (ESI) for Chemical Science.
This journal is © The Royal Society of Chemistry 2022

Supplementary Methods

Discovery of novel drug-like antitubercular hits targeting the MEP pathway enzyme DXPS by strategic application of ligand-based virtual screening

Di Zhu,[a][b][c]† Sandra Johannsen,[a][b]† Tiziana Masini,[c]† Céline Simonin,[d]† Jörg Haupenthal,[a] Boris Illarionov,[e] Anastasia Andreas,[a][b] Mahendra Awale,[d] Robin M. Gierse,[a][b][c] Tridia van der Laan,[f] Ramon van der Vlag,[c] Rita Nasti,[c] Mael Poizat,[g] Eric Buhler,[h] Norbert Reiling,[i][j] Rolf Müller,[a][b][k] Markus Fischer,[e] Jean-Louis Reymond,*[d] Anna K. H. Hirsch*[a][b][c][k]

[a] Helmholtz Institute for Pharmaceutical Research Saarland (HIPS) – Helmholtz Centre for Infection Research (HZI), Campus Building E8.1, 66123 Saarbrücken, Germany.

[b] Department of Pharmacy, Saarland University, Campus Building E8.1, 66123 Saarbrücken, Germany.

[c] Stratingh Institute for Chemistry, University of Groningen, Nijenborgh 7, 9747 AG Groningen, The Netherlands.

[d] Department of Chemistry and Biochemistry, University of Bern, Freiestrasse 3, 3012 Bern, Switzerland.

[e] Hamburg School of Food Science, Institute of Food Chemistry, Grindelallee 117, 20146 Hamburg, Germany.

[f] Department of Mycobacteria, National Institute of Public Health and the Environment (RIVM), Infectious Diseases Research, Diagnostics and Laboratory Surveillance (IDS), Antonie van Leeuwenhoeklaan 9, 3721 MA Bilthoven, The Netherlands.

[g] Syncom BV, Kadijk 3, 9747 AT Groningen, The Netherlands.

[h] Laboratoire Matière et Systèmes Complexes (MSC), UMR CNRS 7057, Université Paris Cité, Bâtiment Condorcet, 75205 Paris Cedex 13, France.

[i] RG Microbial Interface Biology, Research Center Borstel, Leibniz Lung Center, Borstel, Germany.

[j] German Center for Infection Research (DZIF), Partner site Hamburg-Lübeck-Borstel-Riems, Borstel, Germany.

[k] Helmholtz International Lab for Anti-infectives, Helmholtz Centre for Infection Research (HZI), Campus Building E8.1, 66123 Saarbrücken, Germany.

† D. Zhu, S. Johannsen, T. Masini and C. Simonin contributed equally to this work.

1. Table of content

1. Table of content 3
2. Abbreviations 5
3. Supporting methods 6
3.1 Computational methods 6
3.1.1 Compound database for LBVS 6
3.1.2 LBVS 6
3.1.3 Docking 7
3.1.4 Modeling 7
3.2 Biochemical and biological methods 7
3.2.1 Gene expression and protein purification of *Dr*DXPS and *Mt*DXPS 7
3.2.2 Gene expression and protein purification of *E. coli* IspC 8
3.2.3 Gene expression and protein purification of *E. coli* DXPS 8
3.2.4 SDM of *Dr*DXPS 9
3.2.5 MOI study of DZT against *Dr*DXPS, and evaluating DZT against *Dr*DXPS (H304A) and *Mt*DXPS 9
3.2.6 Kinetic study of *Dr*DXPS (WT), *Dr*DXPS (H304A) and *Mt*DXPS 10
3.2.7 Biochemical evaluation of screened inhibitors against *Mt*DXPS 11
3.2.8 Slow-binding of compounds 7, 9 and 10 12
3.2.9 Tight-binding of compounds 7, 9 and 10 12
3.2.10 MOI study of compounds 7, 9 and 10 13
3.2.11 Determination of tight-binding Morrison K_i* of compounds 7, 9, 10 and their derivatives against *Mt*DXPS 14
3.2.12 Determination of dissociation constant (K_d) of compounds 7, 9 and 10 with microscale thermophoresis (MST) 16
3.2.13 Biochemical evaluation of screened inhibitors against mammalian PDH 16
3.2.14 Target validation in *E. coli* strains expressing *Mt*DXPS 17
3.2.15 Determination of MIC of the hits against multiple *M. tuberculosis* strains 17
3.2.16 Metabolic stability 18
3.2.17 Toxicity against human cell lines 19
3.2.18 Toxicity against zebrafish larvae 19
3.2.19 Determination of MIC and frequency of resistance in *E. coli* TolC 19

3.2.20 Determination of mutations of the DXPS coding sequence (CDS) as the mechanism of resistance 20
3.2.21 Hit validation with DLS 20
3.2.22 Hit validation by evaluating DTT influence 21
3.2.23 Hit validation by co-precipitation experiments 22
4. Chemical synthesis and/or characterization 23
4.1 General methods 23
4.2 Characterization of hits 3–5, 7–12 purchased for LBVS 23
4.3 Re-synthesis/synthesis and characterization of hits 7, 9 and 10 and their active derivatives 27
4.3.1 Re-synthesis of hit 7 and synthesis of its active derivatives 27
4.3.2 Re-synthesis of hit 9 and synthesis of its active derivatives 30
4.3.3 Re-synthesis of hit 10 and its active derivatives 33
5. References 37

2. Abbreviations

CFU	colony-forming unit
C. jejuni	*Campylobacter jejuni*
D. radiodurans	*Deinococcus radiodurans*
DLS	dynamic light scattering
DMF	*N*,*N*-dimethylformamide
DR	drug-resistant
DTT	dithiothreitol
DXPS	1-deoxy-d-xylulose-5-phosphate synthase
E. coli	*Escherichia coli*
EDTA	ethylenediaminetetraacetic acid
EGTA	ethylene glycol tetraacetic acid
GAP	glyceraldehyde 3-phosphate
HEPES	(4-(2-hydroxyethyl)-1-piperazine ethanesulfonic acid
HPLC	high-performance liquid chromatography
IspC	1-deoxy-d-xylulose-5-phosphate reductase
LB	lysogeny broth
LBVS	ligand-based virtual screening
M. tuberculosis	*Mycobacterium tuberculosis*
MDR	multidrug-resistant
MIC	minimal inhibitory concentration
MOI	mode of inhibition
MTC	maximum tolerated concentration
NADP	nicotinamide adenine dinucleotide phosphate
OAc	acetate
OD	optical density
PAPS	3'-phosphoadenosine-5'-phosphosulfate
PDH	pyruvate dehydrogenase
SDM	site-directed mutagenesis
TCEP	(tris(2-carboxyethyl)phosphine
THF	tetrahydrofuran
ThDP	thiamine diphosphate
Tris	tris(hydroxymethyl)aminomethane
UDGPA	uridine diphosphate glucuronic acid
XDR	extensively-drug-resistant
XfP	extended atom pair FingerPrint
xLOS	extended Ligand Overlap Score

3. Supporting methods

3.1 Computational methods

3.1.1 Compound database for LBVS

The Princeton catalogue containing ~800K commercially available compounds was downloaded from http://www.princetonbio.com/. Molecules were processed in SMILES format, counter ions were removed (if any), molecules were checked for valence errors, and ionized at pH 7.4 using an in-house written Java program partially utilizing Jchem Chemistry library from ChemAxon Pvt. Ltd. A single lowest-energy 3D-structure was then generated for each compound using CORINA software available from Molecular Networks GmbH.[1] A similar procedure was followed to process the query ligands mentioned in the next section.

3.1.2 LBVS

Three different rounds of LBVS were performed using xLOS, a previously described in-house designed 3D-shape and pharmacophore matching algorithm, and Xfp, a previously described in-house designed topological pharmacophore and shape fingerprint.[2,3] Both xLOS and Xfp were implemented using Java programming language partially utilizing JChem Chemistry library.

First round of virtual screening: The ~800K compounds of the Princeton catalogue were screened independently against three query ligands (DZT, compound **1**, compound **2**) using xLOS. In each case, the input for xLOS was the 3D-structure of a query ligand (sdf file) and a database (sdf file) containing 3D-structures of all the compounds from the Princeton catalog. In each case, the output of xLOS was a SMILES file containing all the compounds from the Princeton catalog, wherein each of the compounds was annotated with its xLOS similarity score to the query compound. For each of the three query ligands, the top-1000 scoring compounds (having highest xLOS similarity scores) from the database were selected. This subset of 3000 compounds was clustered using k-means clustering. Following visual inspection combined with docking results, 67 compounds were selected for purchasing.

Second round of virtual screening: In this round the Princeton catalog was screened independently using xLOS and Xfp fingerprint using five different query compounds (compound **3**, compound **4**, compound **5**, compound **6** and compound **12**). In total, the Princeton catalogue was screened 10 times (2 methods and 5 query compounds). The inputs for the Xfp program were, 2D-structure of a query molecule (SMILES file) and database (SMILES file) containing 2D-structutres of all the compounds from the Princeton database. The output of the Xfp program was the SMILES file containing all the compounds from the Princeton database, wherein each of the compound was annotated with its distance (inverse of similarity) to the query compound. For each query molecule, the top-300 scoring compounds (having highest similarity) from the

Princeton database were selected. The resulting subset of 1500 compounds were clustered and 37 compounds were selected for purchasing based on visual inspection and docking results.

Third round of virtual screening: The second round of virtual screening was repeated to select another small subset of compounds for purchasing.

3.1.3 Docking

The docking was performed using the crystal structure of *Dr*DXS in complex with ThDP (PDB ID: 2O1X).[4] The molecules were docked in the binding pocket of ThDP with the aid of the FlexX docking module in the LeadIT suite.[5] During docking, the binding site in the protein was restricted to 6.5 Å around the co-crystallized ThDP. All docking parameters from LeadIT were used as they were pre-set by the program. The top-scored solutions were used for visualization in MOE.

3.1.4 Modeling

The modelling was performed using the crystal structure of *Dr*DXPS (PDB ID: 2O1X) and the homology model of *Mt*DXPS.[4,6] The docked molecules were modelled in the binding pocket of ThDP with the compute glide of the software MOE.[7] During modelling, the binding site in the protein was restricted to 8.0 Å around the docked molecule. All modelling parameters from MOE were used as they were pre-set by the program.

3.2 Biochemical and biological methods

3.2.1 Gene expression and protein purification of *Dr*DXPS and *Mt*DXPS

Gene expression and protein purification of *Dr*DXPS and *Mt*DXPS followed a previously reported protocol.[8] Cells of *E. coli* BL21(DE3) carrying plasmid pET22b-H6TEVEKDRDXS (for *Dr*DXPS) or pET22b-H6TEVEKMTDXS (for *Mt*DXPS) were inoculated into LB medium supplemented with ampicillin (100 mg/mL) and cultivated in shaking flasks at 37 °C until OD_{590} = 0.4. IPTG was added to 0.5 mM, and the cell culture was further incubated at 20 °C for 20 h. Cells were harvested by centrifugation (4000 rpm, 40 min, 4 °C), washed once with aq. NaCl solution (0.9%) and resuspended in buffer A (50 mM Tris-HCl pH 8.0, 300 mM NaCl, 15 mM imidazole, 0.02% NaN_3), 5 mL per 1 g of cells. Cells in buffer A were disrupted with FrenchPress, debris was removed by centrifugation, and the supernatant was applied to a Ni-chelating sepharose column (1 cm x 15 cm) equilibrated with buffer A. The column was washed with buffer A until OD_{280} of the effluent came back to the base line and then developed with the gradient of imidazole (15–800 mM). Fractions containing DXPS were combined, transferred to buffer B (50 mM Tris-HCl pH 8.0, 100 mM NaCl, 5 mM DTT, 0.02% NaN_3), using desalting column HiPrep 26/10 Desalting (GE Healthcare), concentrated to 19 mg/mL (*Dr*DXPS) or 1.1 mg/mL (*Mt*DXPS) using Amicon Stirred Ultrafiltration Cell (Amicon) equipped with polyethersulfone ultrafiltration membrane

(pore size 10 kDa, Pall Life Sciences) and frozen at −80 °C for long-term storage. Isolated and purified DXPS showed no measurable DXPS activity unless enough external ThDP was added.

3.2.2 Gene expression and protein purification of *E. coli* IspC

Gene expression and purification of *E. coli* IspC were performed according to a literature procedure with modification.[9] 1-Deoxy-D-xylulose-5-phosphate reductoisomerase (IspC) of *E. coli* bearing a His6-tag at its N-terminal end was produced in and purified from recombinant *E. coli* strain BL21(DE3) pQEYAEM. Cells were grown in LB medium in a shaker at 37 °C supplied with ampicillin (100 mg/mL) until the OD_{600} value reached 0.4. Thereafter, IPTG was added to a final concentration of 1 mM, and the cell suspension was incubated further at 30 °C with vigorous agitation for 16 h. Thereafter, cells were harvested by centrifugation, washed once with 0.9% NaCl solution, and frozen at –20 °C for storage. For IspC purification, cell paste (5 g) was resuspended in Tris-HCl buffer (25 mL, 50 mM, pH 8.0), NaCl (300 mM), 0.02% NaN_3, and 15% imidazole and disrupted in French Press; cell debris was centrifuged down, and the supernatant was placed on the top of a Ni-NTA column (1V10 cm). After unbound proteins were washed from the column with the same buffer, the column was developed with an imidazole gradient (15–800 mM). Eluent fractions containing IspC were identified with SDS-polyacrylamide gel electrophoresis, combined, dialyzed versus Tris-HCl (30 mM, pH 8.0), 30 mM NaCl, 1 mM DTT, and 0.02% NaN_3 and concentrated by ultrafiltration and frozen at –80 °C for storage.

3.2.3 Gene expression and protein purification of *E. coli* DXPS

Purification of recombinant *E. coli* DXPS was from previous report.[10] *E. coli* BL21 (DE3) cells harboring dxs-pET37b were grown to OD600 reaching around 0.6 and induced with isopropyl β-D-thiogalactoside (IPTG, 100 µM) at 37 °C. Shaking was continued for 5 hours. Bacterial cells were harvested by centrifugation at 4 °C and stored at –20 °C overnight. Thawed cell pellets were resuspended in protein purification buffer (3 mL of buffer per gram of cell pellet): 50 mM Tris, pH = 8.0, 5 mM $MgCl_2$, 1 mM thiamin diphosphate (ThDP), 10% glycerol (v/v), 1 mM β-mercaptoethanol, 100 µM PMSF, and 100 µM protease inhibitor cocktail (for Histidine-tagged proteins). Cells were then disrupted by sonication. The cell debris was removed by centrifugation at 4 °C. The supernatant was incubated with Ni-NTA in 20 mM imidazole at 4 °C for 1.5 hours. DXP synthase was then eluted from the resin over a stepwise gradient of 20 mM–500 mM imidazole. Elutions were analyzed by SDS-polyacrylamide gel electrophoresis (12%) and stained with Coomassie Brilliant Blue G. Fractions containing a major band at 68.7 kDa were combined and dialyzed at 4 °C overnight against 1 L of 50 mM Tris, pH 8.0, 10% glycerol (v/v), 10 mM $MgCl_2$, and 1 mM ThDP. A second dialysis was carried out in 1 L of 50 mM Tris, pH 8.0, 10% glycerol, 10 mM $MgCl_2$, 1 mM ThDP, and 1 mM β-mercaptoethanol (4 h). Protein concentration was determined by Biorad Protein

Assay with bovine serum albumin (BSA) as a standard. Protein was then flash frozen in liquid N_2 and stored at –80 °C.

3.2.4 SDM of *Dr*DXPS

A pET28a(+) plasmid with a synthetic, codon optimized gene of *D.radiodurans* DXPS, already bearing the His304 to Ala mutation, was kindly shared with us by David J. Merkeler. The expression and purification of the DXPS mutant followed the published protocol by David J. Merkeler with only minor changes.[11] Instead of sonication, the cells were lysed using a Microfluidizer M-110P in a lysis buffer containing 50 mM Tris-HCl pH 8.0, 300 mM NaCl, 20 mM imidazole, 5 mM 2-mercaptoethanol, 2.5 U/mL benzonase and 1 tablet/200 mL Complete Protease inhibitor, EDTA free (Roche). The following purification steps were performed as described by Merkeler and coworkers. Dialysis of the purified enzyme was not performed, instead the pooled, enzyme containing fractions from the chromatography step were diluted three times with double the volume of storage buffer containing 50 mM Tris-HCl pH 8.0, 300 mM NaCl and 5 mM 2-mercaptoethanol and concentrated by ultrafiltration using Vivaspin concentrators with a cut off of 30 kDa. The final yield of the H304A DXPS enzyme was 17.7 mg/L of *E.coli* culture medium. The purity was determined to be >95 % using SDS-PAGE.

3.2.5 MOI study of DZT against *Dr*DXPS, and evaluating DZT against *Dr*DXPS (H304A) and *Mt*DXPS

MOI study of DZT against *Dr*DXPS followed a previously reported protocol.[12] Photometric assays were conducted in transparent flat-bottomed 384-well plates (Nunc® MaxiSorp™). The reaction was carried out in 100 mM Tris-HCl (pH 7.6), and buffer A (color code: red), buffer B (color code: green) and reaction mixture (color code: black) of each assay condition contained the following components:

MOI study of DZT against *Dr*DXPS:

	[ThDP] (µM)	[DTT] (mM)	[$MnCl_2$] (mM)	[NADPH] (mM)	[*Dr*DXPS] (µM)	[IspC] (µM)	[PYR] (mM)	[D-GAP] (mM)
Reference condition	1.0<br>0.5	4.0<br>2.0	4.0<br>2.0	1.0<br>0.5	0.2<br>0.1	16.6<br>8.3	1.0<br>0.5	1.0<br>0.5
Varying [ThDP]	0.2<br>0.1	4.0<br>2.0	4.0<br>2.0	1.0<br>0.5	0.2<br>0.1	16.6<br>8.3	1.0<br>0.5	1.0<br>0.5
Varying [PYR]	1.0<br>0.5	4.0<br>2.0	4.0<br>2.0	1.0<br>0.5	0.2<br>0.1	16.6<br>8.3	0.5<br>0.25	1.0<br>0.5
Varying [D-GAP]	1.0<br>0.5	4.0<br>2.0	4.0<br>2.0	1.0<br>0.5	0.2<br>0.1	16.6<br>8.3	1.0<br>0.5	2.0<br>1.0

Biochemical evaluation of DZT against *Dr*DXPS (H304A):

	[ThDP] (µM)	[DTT] (mM)	[$MnCl_2$] (mM)	[NADPH] (mM)	[*Dr*DXPS] (H304A) (µM)	[IspC] (µM)	[PYR] (mM)	[D-GAP] (mM)
*Dr*DXPS (H304A)	6.0<br>3.0	4.0<br>2.0	4.0<br>2.0	1.0<br>0.5	4.0<br>2.0	16.6<br>8.3	2.0<br>1.0	2.0<br>1.0

Biochemical evaluation of DZT against *Mt*DXPS:

	[ThDP] (µM)	[DTT] (mM)	[$MnCl_2$] (mM)	[NADPH] (mM)	[*Mt*DXPS] (µM)	[IspC] (µM)	[PYR] (mM)	[D-GAP] (mM)
*Mt*DXPS	6.0<br>3.0	4.0<br>2.0	4.0<br>2.0	1.0<br>0.5	2.5<br>1.25	16.6<br>8.3	0.4<br>0.2	1.0<br>0.5

The dilution series was performed in 3.0 µL DMSO in each well, starting from 4 mM in the reaction mixture. The reaction was started by adding 30 µL of buffer A to 30 µL of buffer B. The reaction was monitored photometrically at room temperature at 340 nm using a Synergy H1 (Biotek) microplate reader. Initial rate values were evaluated with a nonlinear regression method using the program Dynafit (Biokin, http://www.biokin.com/dynafit/).[13] MOI information was achieved by comparison of IC_{50} values under different conditions in duplicates.

3.2.6 Kinetic study of *Dr*DXPS (WT), *Dr*DXPS (H304A) and *Mt*DXPS

Kinetic study of *Dr*DXPS (WT), *Dr*DXPS (H304A) and *Mt*DXPS followed a previously reported protocol.[8] Photometric assays were conducted in transparent flat-bottomed 384-well plates (Nunc® MaxiSorp™). The reaction was carried out in 100 mM Tris-HCl (pH 7.6), and buffer A (color code: red), buffer B (color code: green) and reaction mixture (color code: black) of each assay condition contained the following components:

	[ThDP] (µM)	[DTT] (mM)	[$MnCl_2$] (mM)	[NADPH] (mM)	[DXPS] (µM)	[IspC] (µM)	[PYR] (mM)	[D-GAP] (mM)
K_m^{ThDP} (*Dr*DXPS WT)	X<br>0.4–200	4.0<br>2.0	4.0<br>2.0	1.0<br>0.5	0.2<br>0.1	16.6<br>8.3	1.0<br>0.5	1.0<br>0.5
K_m^{PYR} (*Dr*DXPS WT)	30<br>15	4.0<br>2.0	4.0<br>2.0	1.0<br>0.5	0.2<br>0.1	16.6<br>8.3	X<br>0.001 – 1	1.0<br>0.5
$K_m^{D\text{-}GAP}$ (*Dr*DXPS WT)	30<br>15	4.0<br>2.0	4.0<br>2.0	1.0<br>0.5	0.2<br>0.1	16.6<br>8.3	1.0<br>0.5	X<br>0.001–2
K_m^{ThDP}	X	4.0	4.0	1.0	4.0	16.6	2.0	2.0

(*Dr*DXPS H304A)	0.01–10	2.0	2.0	0.5	2.0	8.3	1.0	1.0
K_m^{PYR} (*Dr*DXPS H304A)	200<br>100	4.0<br>2.0	4.0<br>2.0	1.0<br>0.5	4.0<br>2.0	16.6<br>8.3	X<br>0.001 – 1	2.0<br>1.0
$K_m^{D\text{-}GAP}$ (*Dr*DXPS H304A)	200<br>100	4.0<br>2.0	4.0<br>2.0	1.0<br>0.5	4.0<br>2.0	16.6<br>8.3	2.0<br>1.0	X<br>0.001–2
K_m^{ThDP} (*Mt*DXPS)	X<br>0.001–133	4.0<br>2.0	4.0<br>2.0	1.0<br>0.5	2.5<br>1.25	16.6<br>8.3	1.0<br>0.5	1.0<br>0.5
K_m^{PYR} (*Mt*DXPS)	400<br>200	4.0<br>2.0	4.0<br>2.0	1.0<br>0.5	2.5<br>1.25	16.6<br>8.3	X<br>0.001 – 0.3	1.0<br>0.5
$K_m^{D\text{-}GAP}$ (*Mt*DXPS)	400<br>200	4.0<br>2.0	4.0<br>2.0	1.0<br>0.5	2.5<br>1.25	16.6<br>8.3	1.0<br>0.5	X<br>0.001–2

The dilution series was performed in the buffer containing X factor. The reaction was started by adding 30 μL of non X-containing buffer to 30 μL of X containing buffer. The reaction was monitored photometrically at room temperature at 340 nm using a Synergy H1 (Biotek) microplate reader. Initial rate values were evaluated and the kinetic parameters were plotted with a nonlinear regression method using the program Dynafit.

3.2.7 Biochemical evaluation of screened inhibitors against *Mt*DXPS

Biochemical evaluation of screened inhibitors against *Mt*DXPS following a previously reported protocol with some modifications.[8] Photometric assays were conducted in transparent flat-bottomed 384-well plates (Nunc® MaxiSorp™). The reaction was carried out in 100 mM Tris-HCl (pH 7.6), and buffer A (color code: red), buffer B (color code: green) and reaction mixture (color code: black) of each assay condition contained the following components:

[ThDP] (μM)	[DTT] (mM)	[$MnCl_2$] (mM)	[NADPH] (mM)	[*Mt*DXPS] (μM)	[IspC] (μM)	[PYR] (mM)	[D-GAP] (mM)
30	4.0	4.0	1.0	2.5	16.6	1.0	1.0
15	2.0	2.0	0.5	1.25	8.3	0.5	0.5

The dilution series was performed in 3.0 μL DMSO in each well, starting from highest soluble concentrations in the reaction mixture. The reaction was started by adding 30 μL of buffer A to 30 μL of buffer B. The reaction was monitored photometrically at room temperature at 340 nm using a Synergy H1 (Biotek) microplate reader. IC_{50} values were determined with the steady-state reaction rate after the slow-binders reaching the equilibrium, with the software Dynafit.

3.2.8 Slow-binding of compounds 7, 9 and 10

Slow-binding of compounds **7**, **9** and **10** was confirmed by the time-dependent progress curve. Photometric assays were conducted in transparent flat-bottomed 384-well plates (Nunc® MaxiSorp™). The reaction was carried out in 100 mM Tris-HCl (pH 7.6), and buffer A (color code: red), buffer B (color code: green) and reaction mixture (color code: black) of each assay condition contained the following components:

	[ThDP] (µM)	[DTT] (mM)	[$MnCl_2$] (mM)	[NADPH] (mM)	[*Mt*DXPS] (µM)	[IspC] (µM)	[PYR] (mM)	[D-GAP] (mM)
Ref.	30	4.0	4.0	1.0	2.5	16.6	1.0	1.0
(no inh)	15	2.0	2.0	0.5	1.25	8.3	0.5	0.5
**7**	30	4.0	4.0	1.0	2.5	16.6	1.0	1.0
(100 µM)	15	2.0	2.0	0.5	1.25	8.3	0.5	0.5
**9**	30	4.0	4.0	1.0	2.5	16.6	1.0	1.0
(100 µM)	15	2.0	2.0	0.5	1.25	8.3	0.5	0.5
**10**	30	4.0	4.0	1.0	2.5	16.6	1.0	1.0
(100 µM)	15	2.0	2.0	0.5	1.25	8.3	0.5	0.5

Each sample well contains 3.0 µL DMSO stock solution of inhibitors to make the final concentration 100 µM. The reaction was started by adding 30 µL of buffer A to 30 µL of buffer B. Then the time-dependent reaction curve was recorded photometrically at room temperature at 340 nm using a Synergy H1 (Biotek) microplate reader.

3.2.9 Tight-binding of compounds 7, 9 and 10

Slow-binding of compounds **7**, **9** and **10** was confirmed by varying the concentration of *Mt*DXPS. Photometric assays were conducted in transparent flat-bottomed 384-well plates (Nunc® MaxiSorp™). The reaction was carried out in 100 mM Tris-HCl (pH 7.6), and buffer A (color code: red), buffer B (color code: green) and reaction mixture (color code: black) of each assay condition contained the following components:

	[ThDP] (µM)	[DTT] (mM)	[$MnCl_2$] (mM)	[NADPH] (mM)	[*Mt*DXPS] (µM)	[IspC] (µM)	[PYR] (mM)	[D-GAP] (mM)
**7**	30	4.0	4.0	1.0	2.5	16.6	1.0	1.0
	15	2.0	2.0	0.5	1.25	8.3	0.5	0.5
**7**	30	4.0	4.0	1.0	10	16.6	1.0	1.0
	15	2.0	2.0	0.5	5	8.3	0.5	0.5
**9**	30	4.0	4.0	1.0	2.5	16.6	1.0	1.0
	15	2.0	2.0	0.5	1.25	8.3	0.5	0.5
**9**	30	4.0	4.0	1.0	10	16.6	1.0	1.0
	15	2.0	2.0	0.5	5	8.3	0.5	0.5
**10**	30	4.0	4.0	1.0	2.5	16.6	1.0	1.0
	15	2.0	2.0	0.5	1.25	8.3	0.5	0.5
**10**	30	4.0	4.0	1.0	10	16.6	1.0	1.0

15	2.0	2.0	0.5	5	8.3	0.5	0.5

The rest of the steps follows the description in 3.2.7.

3.2.10 MOI study of compounds 7, 9 and 10

Photometric assays were conducted in transparent flat-bottomed 384-well plates (Nunc® MaxiSorp™). The reaction was carried out in 100 mM Tris-HCl (pH 7.6), and buffer A (color code: red), buffer B (color code: green) and reaction mixture (color code: black) of each assay condition contained the following components:

MOI study of compound **7** against *Mt*DXPS:

	[ThDP] (µM)	[DTT] (mM)	[MnCl2] (mM)	[NADPH] (mM)	[*Mt*DXPS] (µM)	[IspC] (µM)	[PYR] (mM)	[D-GAP] (mM)
Reference condition	50<br>25	4.0<br>2.0	4.0<br>2.0	1.0<br>0.5	2.5<br>1.25	16.6<br>8.3	1.0<br>0.5	0.5<br>0.25
Varying [ThDP]	10<br>5	4.0<br>2.0	4.0<br>2.0	1.0<br>0.5	2.5<br>1.25	16.6<br>8.3	1.0<br>0.5	0.5<br>0.25
Varying [PYR]	50<br>25	4.0<br>2.0	4.0<br>2.0	1.0<br>0.5	2.5<br>1.25	16.6<br>8.3	0.6<br>0.3	0.5<br>0.25
Varying [D -GAP]	50<br>25	4.0<br>2.0	4.0<br>2.0	1.0<br>0.5	2.5<br>1.25	16.6<br>8.3	1.0<br>0.5	1.0<br>0.5

MOI study of compound **9** against *Mt*DXPS:

	[ThDP] (µM)	[DTT] (mM)	[MnCl2] (mM)	[NADPH] (mM)	[*Mt*DXPS] (µM)	[IspC] (µM)	[PYR] (mM)	[D-GAP] (mM)
Reference condition	50<br>25	4.0<br>2.0	4.0<br>2.0	1.0<br>0.5	2.5<br>1.25	16.6<br>8.3	0.4<br>0.2	1.0<br>0.5
Varying [ThDP]	10<br>5	4.0<br>2.0	4.0<br>2.0	1.0<br>0.5	2.5<br>1.25	16.6<br>8.3	0.4<br>0.2	1.0<br>0.5
Varying [PYR]	50<br>25	4.0<br>2.0	4.0<br>2.0	1.0<br>0.5	2.5<br>1.25	16.6<br>8.3	0.2<br>0.1	1.0<br>0.5
Varying [D-GAP]	50<br>25	4.0<br>2.0	4.0<br>2.0	1.0<br>0.5	2.5<br>1.25	16.6<br>8.3	0.4<br>0.2	2.0<br>1.0

MOI study of compound **10** against *Mt*DXPS:

	[ThDP] (µM)	[DTT] (mM)	[MnCl2] (mM)	[NADPH] (mM)	[*Mt*DXPS] (µM)	[IspC] (µM)	[PYR] (mM)	[D-GAP] (mM)
Reference condition	30<br>15	4.0<br>2.0	4.0<br>2.0	1.0<br>0.5	2.5<br>1.25	16.6<br>8.3	0.4<br>0.2	1.0<br>0.5
	6	4.0	4.0	1.0	2.5	16.6	0.4	1.0

Varying [ThDP]	3	2.0	2.0	0.5	1.25	8.3	0.2	0.5
Varying	30	4.0	4.0	1.0	2.5	16.6	0.2	1.0
[PYR]	15	2.0	2.0	0.5	1.25	8.3	0.1	0.5
Varying	30	4.0	4.0	1.0	2.5	16.6	0.4	2.0
[D-GAP]	15	2.0	2.0	0.5	1.25	8.3	0.2	1.0

The dilution series was performed in 3.0 µL DMSO in each well, starting from highest soluble concentrations in the reaction mixture. The reaction was started by adding 30 µL of buffer A to 30 µL of buffer B. The reaction was monitored photometrically at room temperature at 340 nm using a Synergy H1 (Biotek) microplate reader. IC_{50} values were determined with the steady-state reaction rate after the slow-binders reaching the equilibrium, with the software Dynafit.

MOI study of compound **7**, **9** and **10** against *Dr*DXPS follows the description in 3.2.5.

3.2.11 Determination of tight-binding Morrison K_i* of compounds 7, 9, 10 and their derivatives against *Mt*DXPS

Determination of K_i* against *Mt*DXPS follows the previously reported protocol on *Dr*DXPS.[9,14] The tight-binding Morrison model is proposed based on a random sequential mechanism of *Mt*DXPS:

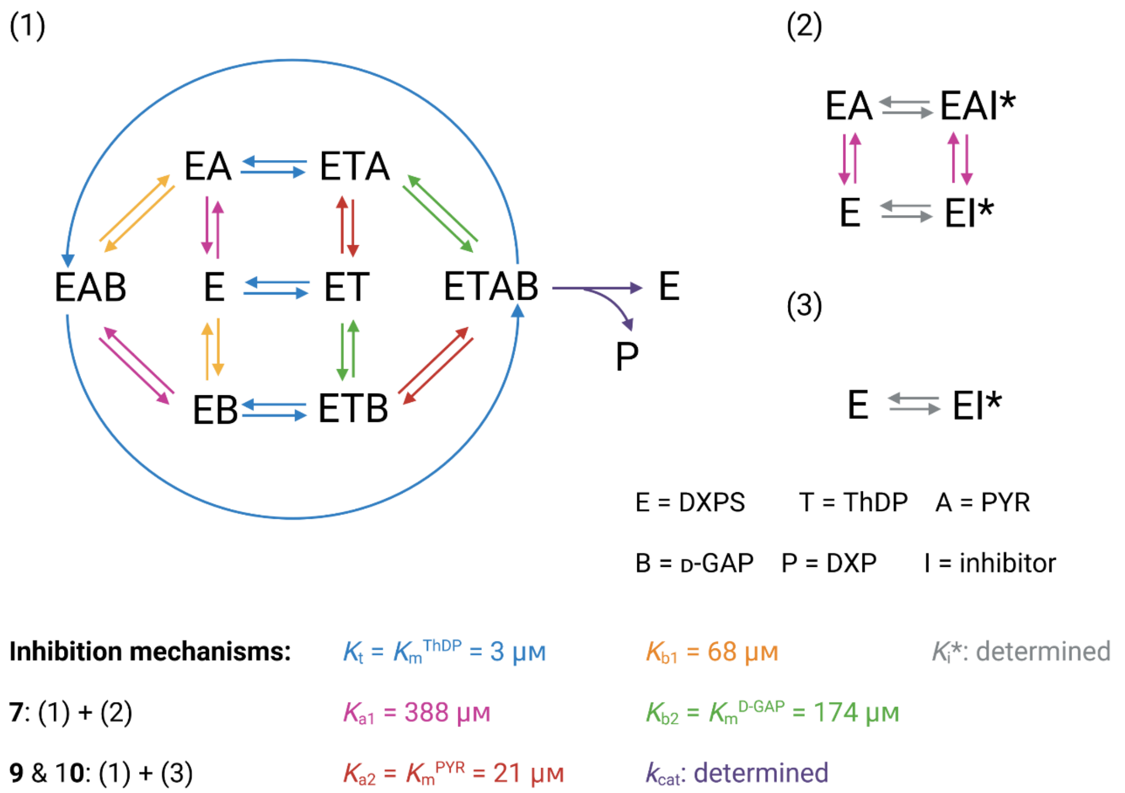

As shown in the figure above, the reaction system can be described in two parts: (1), the catalytic reaction of *Mt*DXPS (E), which follows the random sequential mechanism; (2) or (3), the tight-binding Morrison model of the inhibitor interacting with E. The MOI of compound **7**, which is competitive with ThDP (T) and D-GAP (B), but noncompetitive with pyruvate (A), could be described by the mechanisms (1) + (2); while the MOI of compounds **9** and **10**, which are competitive with T, A and B, can be described as mechanisms (1) + (3). With the known parameters, K_i* and k_{cat} could be determined with the proposed inhibition mechanisms via Dynafit. The detailed equations in Dynafit are listed below:

Compound **7** (competitive with ThDP and D-GAP, but noncompetitive with pyruvate):

E + T <===> ET : K_t (K_t is the dissociation constant of ThDP with DXPS, which equals)

E + A <===> EA : K_{a1} (K_{a1} is the dissociation constant of pyruvate with apoDXPS)

E + B <===> EB : K_{b1} (K_{b1} is the dissociation constant of D-GAP with apoDXPS)

ET + A <===> ETA : K_{a2} (K_{a2} is the dissociation constant of pyruvate with holoDXPS)

ET + B <===> ETB : K_{b2} (K_{b2} is the dissociation constant of D-GAP with holoDXPS)

ETB + A <===> ETAB : K_{a2} (K_{a2} is the dissociation constant of pyruvate with holoDXPS)

ETA + B <===> ETAB : K_{b2} (K_{b2} is the dissociation constant of ThDP with DXPS)

EA + I <===> EAI* : K_i* (K_i* is the Morrison inhibition constant of inhibitor with pyruvate-bound DXPS)

E + I <===> EI* : Ki* (K_i* is the Morrison inhibition constant of inhibitor with apoDXPS)

ETAB ---> ET + P : k_{cat} (k_{cat} is the catalytic constant of DXPS)

Compounds **9** and **10** (competitive with ThDP, pyruvate and D-GAP):

E + T <===> ET : K_t (K_t is the dissociation constant of ThDP with DXPS, which equals)

E + A <===> EA : K_{a1} (K_{a1} is the dissociation constant of pyruvate with apoDXPS)

E + B <===> EB : K_{b1} (K_{b1} is the dissociation constant of D-GAP with apoDXPS)

ET + A <===> ETA : K_{a2} (K_{a2} is the dissociation constant of pyruvate with holoDXPS)

ET + B <===> ETB : K_{b2} (K_{b2} is the dissociation constant of D-GAP with holoDXPS)

ETB + A <===> ETAB : K_{a2} (K_{a2} is the dissociation constant of pyruvate with holoDXPS)

ETA + B <===> ETAB : K_{b2} (K_{b2} is the dissociation constant of ThDP with DXPS)

E + I <===> EI* : Ki* (K_i* is the Morrison inhibition constant of inhibitor with apoDXPS)

ETAB ---> ET + P : k_{cat} (k_{cat} is the catalytic constant of DXPS)

3.2.12 Determination of dissociation constant (K_d) of compounds 7, 9 and 10 with microscale thermophoresis (MST)

MST measurements were performed on a Monolith NT.115 (NanoTemper Technologies GmbH, Germany) instrument using red-dye-NHS fluorescent labeling. Each purified *Mt*DXPS sample was labeled with red-dye-NHS second generation dye, according to the supplied protocol. Measurements were performed in 100 mM Tris-HCl (pH 7.6) containing 0.05% Tween-20 and 5% DMSO in premium treated capillaries. The final concentration of each labeled protein in the assay was 100 nM. The inhibitors were titrated in 1:1 dilutions according to the supplier's recommendations. All binding reactions were incubated at room temperature for 30 min before loading, and then pre-incubated for 5 min at room temperature after loading into the capillaries. Experiments were performed in duplicates, using 40% LED power, medium MST power, laser on time 20 s, and laser off time 3 s. K_ds of the inhibitors were extracted from raw data at a 10–20 s on time according to the manufacturer's instructions. A denaturation test was performed to exclude any nonspecific spectral interactions between the inhibitors and the red-dye.

3.2.13 Biochemical evaluation of screened inhibitors against mammalian PDH

Bio-evaluation of screened inhibitors against mammalian PDH followed a previously reported protocol.[15] PDH from porcine heart was purchased from Sigma Aldrich as a buffered aqueous 50% glycerol solution containing approximately 9 mg/mL bovine serum albumin, 30% sucrose, 1.5 mM EDTA, 1.5 mM EGTA, 1.5 mM 2-mercaptoethanol, 0.3% TRITON® X-100, 0.003% sodium azide, and 15 mM potassium phosphate, pH 6.8. Each inhibitor was diluted in 100% DMSO, and bidistilled water was slowly added. The solutions were vortexed, and then the rest of the assay components was added in the following order: HEPES, pH = 8 (100 mM), bovine serum albumin (1 mg/mL), cysteine (2 mM), ThDP (0.325 μM), coenzyme-A (150 μM), TCEP (300 μM), pyruvate (150 μM) and $MgCl_2$ (1 mM). The final % of DMSO in the assay media was 10%. The assay was carried out as previously described. The UV/Vis spectrophotometer (Beckman Coulter DU800) was first blanked with each solution, and then NAD^+ was added to a final concentration of 500 μM. The solutions were then preincubated at 30 °C for 5 min. Porcine PDH (final concentration of 0.01 U/mL) was then added to initiate the reactions, which were monitored spectrophotometrically by measuring the

appearance of NADH at 340 nm over time at 30 °C. Initial rates of product formation (plotted as [NADH] versus time) were determined using Microsoft Excel.

3.2.14 Target validation in *E. coli* strains expressing *Mt*DXPS

The *E. coli* BL21(DE3) strain carrying plasmid pQEYAEM for *E.coli* IspC, and plasmid pET22b-H6TEVEKMTDXS for *Mt*DXPS overexpression was the same with described above. Target validation followed the previously reported protocol with small modifications.[16] Starting OD_{600} 0.03 was used in a total volume of 200 µL in LB containing the compounds predissolved in DMSO (maximal DMSO concentration in the experiment: 5%). The final compound concentrations prepared from serial dilutions were:

Cmpd No.	Conc. range (µM)
**7**	0.4–100
**9**	0.4–200
**10**	0.4–200
**FSM**	0.4–800

Each assay was repeated in duplicates. The end-point ODs were determined after addition of the compounds and again after incubation for 18 h at 37 °C and 50 rpm in 96-well plates (Sarstedt, Nümbrecht, Germany) using a FLUOStar Omega (BMG labtech, Ortenberg, Germany). Given MIC values are means of three independent determinations and are defined as the lowest concentration at which bacterial growth was reduced by >95%. Target information could be achieved by comparison of MIC in absence and presence of 0.5 mM IPTG.

3.2.15 Determination of MIC of the hits against multiple *M. tuberculosis* strains

Determination of MIC of the hits against multiple *M. tuberculosis* strains followed a previously reported protocol.[17] A series of drug-susceptible, DR, MDR and XDR *M. tuberculosis* isolates, and one control strain (H37Rv) were selected and subcultured on a Middlebrook 7H10 agar medium until use. Susceptibility testing using the absolute concentration method was carried out by preparing 25-well plates with solid 7H10 medium containing different concentrations (20, 10, 5, 2.5, 1.25 µM) of each inhibitor. The plates were subsequently inoculated by adding 10 µL *M. tuberculosis* suspension to each well. Then the plates were incubated at 35.5 °C in a CO_2 incubator. After appropriate incubation, the MICs were assessed. The reading of the plates was carried out when the bacterial growth on the two control wells without inhibitor was sufficient, i.e., when colonies were clearly visible.

Compounds **17** and **25** were tested for their Mtb growth inhibitory capacity in liquid culture as previously described.[12] Tests were performed in 2-fold serial dilutions starting at 64 µM in triplicates (2×10^6 mCherry-Mtb H37Rv bacteria, volume 100 µL).

Bacterial growth was measured after 7 days of culture as described. Obtained values were normalized to solvent control (DMSO-treated bacteria set to 100%).

3.2.16 Metabolic stability

For vitro assessment of metabolic stability, 10 µL of each compound (10 µM) was incubated with human pooled liver S9-fraction (1 mg/mL, Corning, Corning, NY, USA) in a master mix containing 2 mM NADP, 10 mM glucose-6-phosphate, 10 U/mL glucose-6-phosphate dehydrogenase, 1 mM UDPGA, 0.1 mM PAPS, 10 mM $MgCl_2$. After incubating for 0, 5, 15, 30, and 60 min the reaction was stopped by adding 200 µL acetonitrile containing diphenhydramine as internal standard at 4 °C. The supernatant was used for LC-MS analysis and half life time $t_{1/2}$ was calculated from decreasing concentrations/peak areas.

LC conditions: samples were analyzed with an Ultimate 3000 HPLC (Thermo Fisher, Waltham, MA, USA) coupled to a TSQ Quantum Access Max triple quadrupole mass spectrometer (Thermo Fisher, Waltham, MA, USA) in SRM mode. Analysis was performed on a Nucleodur C18 Pyramid column (3 µm, 2x150 mm, Macherey-Nagel, Düren, Germany) with a flow of 0.6 mL/min and a water (0.1% formic acid) and acetonitrile (0.1% formic acid) eluent system. Gradient as follows: 10% acetonitrile for 1 min, 10–90% acetonitrile over 0.7 min, 90% acetonitrile for 1.8 min, equilibration at 10% acetonitrile for 1 min.

MS conditions: calibration of mass spectrometer parameters was performed individually for each compound:

	**7**	**9**	**10**
polarity	positive	positive	positive
spray voltage	3500	3500	3500
vaporizer temperature	0	350	350
sheath gas pressure	20	30	30
aux gas pressure	55	35	35
capillary temperature	275	350	350
collision pressure	1.5	1.5	1.5
collision energy	62/42/43/26	2/26/19/18	30/32/29/25
tube lens offset	75	104	124
precursor ion	287.981 $[M-H_2O+H]^+$	278.03	357.01
fragment ion 1	89.05	125.03	112.05
fragment ion 2	123.04	136.07	253.92
fragment ion 3	124.08	167.99	280.90
fragment ion 4	158.93	260.98	336.94

3.2.17 Toxicity against human cell lines

HepG2, A549 or HEK293 cells (2 x 10^5 cells per well) were seeded in 24-well, flat-bottomed plates. Culturing of cells, incubations and OD measurements were performed as described previously with small modifications.[18] Twenty-four hours after seeding the cells, the incubation was started by the addition of compounds in a final DMSO concentration of 1%. The metabolic activity of the living cell mass was determined after 48 h. At least three independent measurements were performed for each compound.

3.2.18 Toxicity against zebrafish larvae

Ethics: All of the described experiments were performed with zebrafish embryos <120 h post fertilization (hpf) and are not classified as animal experiments according to EU Directive 2010/63/EU. Protocols for husbandry and care of adult animals are in accordance with the German Animal Welfare Act (§11 Abs. 1 TierSchG).

Materials and Methods: MTC: Zebrafish husbandry was performed according to standard procedures. Zebrafish of the TLF wild-type line (obtained from Prof. R. Köster, TU Braunschweig, Germany) were raised at 27 °C. Embryos were collected and kept in a Petri dish until the next day in 0.3x Danieau's medium (17 mM NaCl, 2 mM KCl, 1.8 mM $Ca(NO_3)_2$, 1.5 mM HEPES (pH 7.1–7.3), 0.12 mM $MgSO_4$ and 1.2 µM methylene blue). Toxicity testing was performed using embryos at 1 day post fertilization (dpf). The embryos were placed in 96-well plates with one embryo per well and treated with the test compound solutions. Larvae were monitored daily (up to 120 hpf) to assess developmental defects and survival rates. Compound solutions were prepared using 0.3x Danieau's medium to obtain concentrations between 2 µM and 100 µM with a final DMSO concentration of 1% (v/v).

3.2.19 Determination of MIC and frequency of resistance in *E. coli* TolC

MIC determination (of **7**, **9**, **10** and references) in *E. coli* TolC was performed as previously described.[16] Starting OD_{600} 0.03 was used in a total volume of 200 µL in LB containing the compounds predissolved in DMSO (maximal DMSO concentration in the experiment: 1%). Final compound concentrations prepared from serial dilutions ranged from 0.02 to 100 µM (duplo values for each concentration) depending on their antibacterial activity and the observation of compound precipitation in the growth medium. The ODs were determined after addition of the compounds and again after incubation for 18 h at 37 °C and 50 rpm in 96-well plates (Sarstedt, Nümbrecht, Germany) using a FLUOStar Omega (BMG labtech, Ortenberg, Germany). Given MIC values are means of three independent determinations and are defined as the lowest concentration at which bacterial growth was reduced by >95%.

To calculate the frequency of resistance (FoR) of selected compounds, a defined number of CFU of *E. coli* TolC was plated on LB agar plates containing 2x or 4x MIC

concentration of **10**, Rifampicin, Chloramphenicol or Ciprofloxacin. After incubation at 37 °C overnight, colonies were counted and related to the total initial number of CFU. The given values are means of three independent experiments.

3.2.20 Determination of mutations of the DXPS coding sequence (CDS) as the mechanism of resistance

E. coli TolC colonies resistant against **10** were used from the experiment described in 3.2.19. Ten resistant colonies, as well as two control colonies were chosen. For each colony, 2 mL of LB medium was inoculated and grown at 37 °C overnight at 180 rpm. The genomic DNA was isolated and sent for sequencing. All ten resistant clones, as well as the two control clones, had no mutation in the DXPS-encoding region (1-deoxy-D-xylulose-5-phosphate synthase CDS: 3,626,320 -> 3,628,182) and 100% sequence identity to the parent *E. coli* TolC strain with the Genebank accession code NZ_CP018801.

3.2.21 Hit validation with DLS

Hits **7**, **9** and **10** were dissolved in assay buffer Tris-HCl (pH 7.6) at 36 µM, 56 µM and 36 µM, respectively. The solutions were submitted for DLS to exclude false positive inhibition caused by aggregation or precipitation, following a previously reported protocol.[19] The measurements used a 3D DLS spectrometer (LS Instruments, Fribourg, Switzerland) equipped with a 25 mW HeNe laser (JDS uniphase) operating at λ=632.8 nm, a two channel multiple tau correlator (1088 channels in autocorrelation), a variable-angle detection system, and a temperature-controlled index matching vat (LS Instruments). The scattering spectrum was measured using two single mode fibre detections and two high sensitivity APD detectors (Perkin Elmer, model SPCM-AQR-13-FC).

Fluctuations in the scattered intensity with time $I(q,t)$ (also called count rate), measured at a given scattering angle θ or equivalently at a given scattering wave vector $q = (4\pi n/\lambda)\sin(\theta/2)$ where n is the refractive index (equal to 1.33 at 20 °C for water), are directly reflecting the so-called Brownian motion of the scattering particles. In DLS, the fluctuation pattern is translated into the normalized time autocorrelation function of the scattered intensity, $g^{(2)}(q,t)$ defined as:

$$g^{(2)}(q,t) = \frac{\langle I(q,0)I(q,t)\rangle}{\langle I(q,0)\rangle^2} \tag{S1}$$

It is related to the so-called dynamic structure factor (or concentration fluctuations autocorrelation function), $g^{(1)}(q,t)$, via the Siegert relation:

$$g^{(2)}(q,t) - 1 = \beta\left|g^{(1)}(q,t)\right|^2 \tag{S2}$$

Where β is the coherence factor, which in our experiments is varying between 0.4 and 0.6, depending on the samples and the setup geometry. The normalized dynamical correlation function, $g^{(1)}(q,t)$, of concentration fluctuations is defined as:

$$g^{(1)}(q,t) = \frac{\langle \delta c(q,0)\delta c(q,t)\rangle}{\langle \delta c(q,0)^2\rangle} \tag{S3}$$

Where $\delta c(q,t)$ and $\delta c(q,0)$ represent fluctuations of the concentration at time t and zero, respectively. The distribution of decay rates $G(\Gamma)$ was determined using the CONTIN algorithm based on the inverse Laplace transform of $g^{(1)}(q,t)$:

$$g^{(1)}(q,t) = \int_0^\infty G(\Gamma)\exp(-\Gamma t)d\Gamma \tag{S4}$$

For a diffusive process, with characteristic time, $\tau = 1/\Gamma$, inversely proportioned to q^2, $g^{(1)}(q,t) \sim \exp(-Dq^2t)$, with D the mutual diffusion coefficient. The Stokes-Einstein relation allows one to determine the hydrodynamic radius R_h of the scattered objects; $R_h = kT/6\pi\eta D$, if the temperature T and solvent viscosity η are known (here η = 1.002 cP at 20 °C for water). In our experiments, solutions were characterized by a single relaxation mechanism. We have then also adopted the cumulant analysis:

$$ln g^{(1)}(q,t) = k_0 - k_1 t + \frac{k_2}{2}t^2 + \cdots \tag{S5}$$

Where $k_1 = 1/<\tau>$ and k_2/k_1^2 represents the polydispersity index. The extrapolation of $(<\tau>q^2)^{-1}$ to q = 0 yields the mutual diffusion coefficient D. The results were shown in **Figure S5A** displaying correlation functions obtained at scattering angle of 60° and hydrodynamic radii distributions.

3.2.22 Hit validation by evaluating DTT influence

Reaction of active hits against *Mt*DXPS following a previously reported protocol.[20] Photometric assays were conducted in transparent flat-bottomed 384-well plates (Nunc® MaxiSorp™). The reaction was carried out in 100 mM Tris-HCl (pH 7.6), and buffer A (color code: red), buffer B (color code: green) and reaction mixture (color code: black) of each assay condition contained the following components:

[ThDP] (µM)	[DTT] (mM)	[$MnCl_2$] (mM)	[NADPH] (mM)	[*Mt*DXPS] (µM)	[IspC] (µM)	[PYR] (mM)	[D-GAP] (mM)
30	20	4.0	1.0	2.5	16.6	1.0	1.0
15	10	2.0	0.5	1.25	8.3	0.5	0.5

The dilution series was performed in 3.0 µL DMSO in each well, starting from highest soluble concentrations in the reaction mixture. The reaction was started by adding 30 µL of buffer A to 30 µL of buffer B. The reaction was monitored photometrically at

room temperature at 340 nm using a Synergy H1 (Biotek) microplate reader. IC_{50} values with high concentration of DTT were then determined with Dynafit and compared with the normal IC_{50}s as described in 3.2.7.

3.2.23 Hit validation by co-precipitation experiments

Co-precipitation of active hits with *Mt*DXPS following a previously reported protocol with some modifications.[21] The dilution series was performed in 10.0 µL DMSO in 2 mL Eppendorf tubes, starting from highest soluble concentrations in the reaction mixture. The DMSO solutions were then dissolved in 200 µL Tris-HCl (100 mM, pH 7.6) containing 1.25 µM *Mt*DXPS. After ultra-centrifugation at 16000g for 30 min, the protein concentrations in the supernatant of each Eppendorf tube was determined with calibrated extinction coefficient of *Mt*DXPS.

4. Chemical synthesis and/or characterization

4.1 General methods

NMR experiments were run on a Bruker Ultrashield plus 500 (500 MHz) spectrometer. Spectra were acquired at 300 K, using deuterated dimethylsulfoxide, deuterated methanol or deuterated chloroform as solvent. Chemical shifts for ^{1}H and ^{13}C spectra were recorded in parts per million (ppm) using the residual non-deuterated solvent as the internal standard.

Solvent	^{1}H chemical shift in ppm	^{13}C chemical shift in ppm
**$(CD_3)_2OS$**	2.50	39.52
**CD_3OD**	3.31	49.00
**$CDCl_3$**	7.26	77.16

Coupling constants (*J*) are given in Hertz. Data are reported as follows: Chemical shift, multiplicity (s = singlet, d = doublet, t = triplet, m = multiplet, br = broad and combinations of these) coupling constants and integration. Mass spectrometry was performed on a SpectraSystems-MSQ LCMS system (Thermo Fisher, Dreieich, Germany). Flash chromatography was performed using the automated flash chromatography system CombiFlash Rf+ (Teledyne Isco, Lincoln, NE, USA) equipped with RediSepRf silica columns (Axel Semrau, Sprockhövel Germany). The purity of the final compounds was determined using Dionex Ultimate 3000 HPLC system (Thermo Fisher Scientific). Chromatographic separation was performed on an EC 150/2 Nucleodur C18 Pyramid (3 µm particle size) analytical column (Macherey-Nagel). The mobile phase consisted of solvent A (acetonitrile containing 0.1% formic acid) and solvent B (water containing 0.1% formic acid) with a flow rate of 0.25 ml/min.

All compounds had a purity >95%.

All chemicals were purchased at SigmaAldrich or comparable commercial suppliers and used without further purification.

4.2 Characterization of hits 3–5, 7–12 purchased for LBVS

2-(1-(4-Isopropylbenzyl)-1*H*-benzo[d]imidazol-2-yl)ethanol (**3**, supplier ID: OSSK_927261)

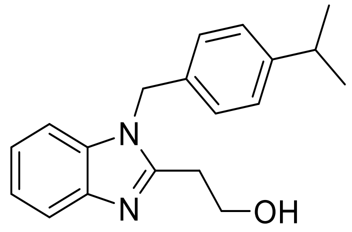

^{1}H-NMR (300 MHz, $(CD_3)_2OS$): δ = 7.61 – 7.53 (m, 1H), 7.49 – 7.41 (m, 1H), 7.22 – 7.11 (m, 4H), 7.03 (d, *J* = 8.2 Hz, 2H), 5.46 (s, 2H), 4.85 (t, *J* = 5.5 Hz, 1H), 3.85 (q, *J*

= 6.8 Hz, 2H), 3.00 (t, *J* = 6.8 Hz, 2H), 2.82 (d, *J* = 6.9 Hz, 1H), 1.15 (d, *J* = 6.9 Hz, 6H). LC-ESI-MS: calculated for $[C_{19}H_{23}N_2O]^+$: 295.18, found 295.12.

2-Amino-6,7-dihydro-6-(1-(4-isopropylphenyl)ethyl)pyrrolo[3,4-d]pyrimidin-5-one (**4**, supplier ID: OSSL_332078)

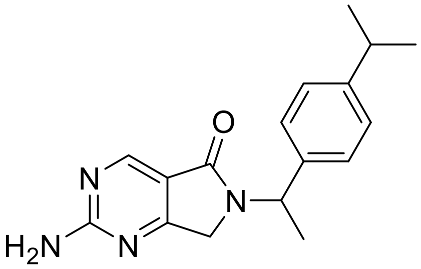

^{1}H-NMR (300 MHz, $(CD_3)_2OS$): δ = 8.51 (s, 1H), 7.38 (brs, 2H), 7.29 – 7.16 (m, 4H), 5.41 (q, *J* = 7.2 Hz, 1H), 4.37 (d, *J* = 18.4 Hz, 1H), 3.91 (d, *J* = 18.4 Hz, 1H), 2.85 (hept, *J* = 6.9 Hz, 1H), 1.57 (d, *J* = 7.2 Hz, 3H), 1.17 (d, *J* = 6.9 Hz, 6H). LC-ESI-MS: calculated for $C_{17}H_{21}N_4O$ [*M*+H]$^+$: 297.17, found 297.11.

4-[(4-Fluorophenyl)(hydroxyimino)methyl]benzene-1,3-diol (**5**, supplier ID: OSSL_151911)

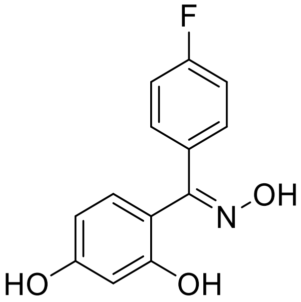

This compound was purified by RP-HPLC (gradient 85% A 15% D to 100% D in 40 minutes) prior to testing it. ^{1}H-NMR (300 MHz, $(CD_3)_2OS$): δ = 11.37 (s, 1H), 11.26 (s, 1H), 9.77 (s, 1H), 7.39 – 7.25 (m, 4H), 6.53 (d, *J* = 8.6 Hz, 1H), 6.31 (d, *J* = 2.4 Hz, 1H), 6.21 (dd, *J* = 2.4, 8.6 Hz, 1H). LC-ESI-MS: calculated for $C_{13}H_{11}FNO_3$ [*M*+H]$^+$: 248.17, found 248.02.

(1-(3,4-Dichlorobenzyl)-1*H*-indol-3-yl)methanol (**7**, supplier ID: OSSL_652383)

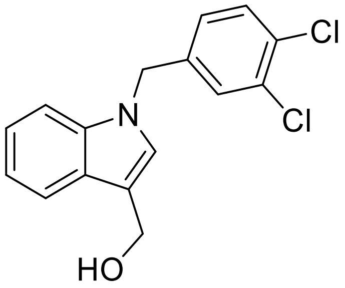

Compound **7** was purchased from Specs. ^{1}H-NMR (300 MHz, $(CD_3)_2OS$): δ = 7.61 (d, *J* = 7.8 Hz, 1H), 7.57 (d, *J* = 8.3 Hz, 1H), 7.50 (d, *J* = 2.0 Hz, 1H), 7.46–7.39 (m, 2H), 7.15 (dd, *J* = 2.0, 8.3 Hz, 1H), 7.12 – 7.07 (m, 1H), 7.06–6.99 (m, 1H), 5.39 (s, 2H), 4.82 (t, *J* = 5.3 Hz, 1H), 4.64 (d, *J* = 5.3 Hz, 2H). LC-ESI-MS: calculated for $C_{16}H_{13}Cl_2O$ [*M*-OH]$^+$: 288.03 (^{35}Cl), 290.03 (^{37}Cl), found 288.03, 290.03. Purity of the sample could not be determined by RP-UHPLC because it was insoluble in the eluents required for the measurement.

N-(3-Chloro-2-methylphenyl)-4-(2,4-dimethylthiazol-5-yl)thiazol-2-amine (**8**, supplier ID: OSSK_523229)

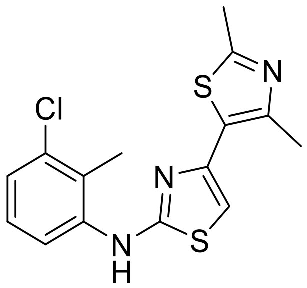

^{1}H-NMR (300 MHz, $(CD_3)_2OS$): δ = 9.63 (s, 1H), 7.92 – 7.80 (m, 1H), 7.28 –7.16 (m, 2H), 6.93 (s, 1H), 2.57 (s, 3H), 2.48 (s, 3H), 2.32 (s, 3H). LC-ESI-MS: calculated for $C_{15}H_{15}ClN_3S_2$ [*M*+H]$^+$: 336.04 (^{35}Cl), 338.04 (^{37}Cl), found 336.12, 338.01.

4-[(4-Chlorobenzyl)(hydroxyimino)methyl]benzene-1,3-diol (**9**, supplier ID: OSSK_862931)

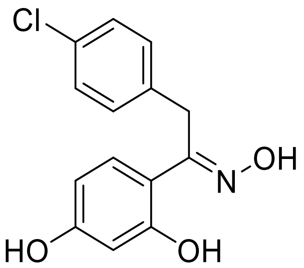

(*E*)-**21** was purchased from Vitas-M. ^{1}H-NMR (300 MHz, $(CD_3)_2OS$): δ = 11.65 (s, 1H), 11.50 (s, 1H), 9.72 (s, 1H), 7.34 (d, *J* = 8.7 Hz, 2H), 7.30 – 7.22 (m, 3H), 6.28 – 6.22 (m, 2H), 4.16 (s, 2H). LC-ESI-MS: calculated for $C_{14}H_{13}ClNO_3$ [*M*+H]$^+$: 278.06 (^{35}Cl), 280.06 (^{37}Cl), found 278.12. 280.11.

4-(2-Amino-4-methylthiazol-5-yl)-*N*-(4-(trifluoromethyl)phenyl)thiazol-2-amine (**10**, supplier ID: OSSL_650294)

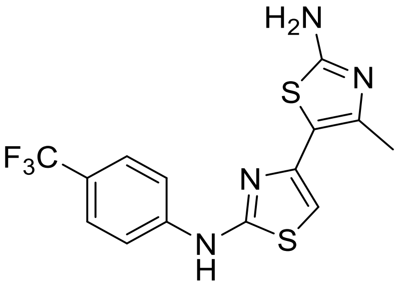

^{1}H-NMR (300 MHz, $(CD_3)_2OS$): δ = 10.65 (s, 1H), 7.82 (d, *J* = 8.46 Hz, 2H), 7.65 (d, *J* = 8.46 Hz, 2H), 7.01 (s, 2H), 6.72 (s, 1H), 2.33 (s, 3H). LC-ESI-MS: calculated for $C_{14}H_{12}F_3N_4S_2$ $[M+H]^+$: 357.05, found 357.14.

2-(2-Hydroxyethylamino)-3-((*Z*)-(3-cyclohexyl-4-oxo-2-thioxothiazolidin-5-ylidene)methyl)-4*H*-pyrido[1,2-a]pyrimidin-4-one (**11**, supplier ID: OSSK_026083)

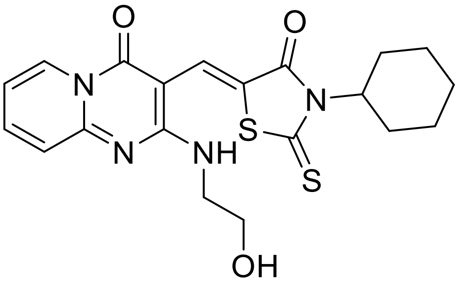

^{1}H-NMR (300 MHz, $(CD_3)_2OS$): δ = 8.77 (dd, *J* = 1.6, 6.7 Hz, 1H), 8.07 (br s, 1H), 7.89 (ddd, *J* = 1.6, 6.7, 8.7 Hz, 1H), 7.64 (s, 1H), 7.34 (d, *J* = 8.7 Hz, 1H), 7.13 (td, *J* = 1.3, 6.7 Hz, 1H), 4.93 (br t, *J* = 12.1 Hz, 1H), 4.77 (t, *J* = 5.5 Hz, 1H), 3.68 – 3.50 (m, 4H), 2.45 – 2.22 (m, 2H), 1.93 – 1.75 (m, 2H), 1.75 – 1.54 (m, 3H), 1.44 – 1.06 (m, 3H). LC-ESI-MS: calculated for $C_{20}H_{23}N_4O_3S_2$ $[M+H]^+$: 431.12, found 431.12. Purity of the sample by RP-UHPLC could not be determined because it was insoluble in the eluents required for the measurement.

4-(2,4-Dichlorophenyl)-4,10-dihydro-1*H*-[1,3,5]triazino[1,2-a]benzimidazole-2-amine (**12**, supplier ID: OSSK_676817)

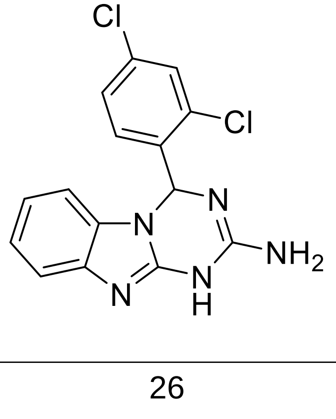

^{1}H-NMR (300 MHz, $(CD_3)_2OS$): δ = 8.04 (br s, 1H), 7.73 (d, *J* = 2.0 Hz, 1H), 7.45 (dd, *J* = 2.0, 7.8 Hz, 1H), 7.26 (d, *J* = 7.3 Hz, 1H), 7.08 (m, 2H), 6.96 (t, *J* = 7.3 Hz, 1H), 6.80 (t, *J* = 7.3 Hz, 1H), 6.62 (d, *J* = 7.8 Hz, 1H), 6.40 (br s, 2H). LC-ESI-MS: calculated for $C_{15}H_{12}Cl_2N_5^+$ $[M+H]^+$: 332.05, (^{35}Cl), 334.04 (^{37}Cl), found 332.14, 334.07.

4.3 Re-synthesis/synthesis and characterization of hits 7, 9 and 10 and their active derivatives

4.3.1 Re-synthesis of hit 7 and synthesis of its active derivatives

4.3.1.1 General procedures

S_N2 substitution

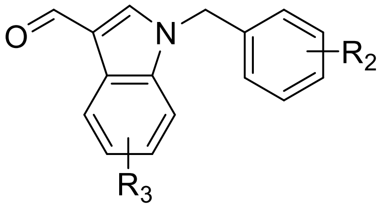

The 1-substituted-1*H*-indole-3-carbaldehyde was synthesized by following a previously reported procedure.[22] Sodium hydride (1.51 g, 63 mmol, 1.8 eq.) was suspended in *N,N*-dimethylformamide (DMF, 90 mL) and the suspension was cooled to 0 °C under nitrogen atmosphere. 1*H*-Indole-3-carbaldehyde (5.0 g, 34 mmol, 1.0 eq.) was added. The mixture was stirred at 25 °C for 30 minutes, after which 3,4-dichlorobenzylbromide (6.0 mL, 41 mmol, 1.2 eq.) was added. The resulting mixture was stirred for 16 h. Water (500 mL) and ethyl acetate (400 mL) were added, and the layers were separated. The aqueous layer was extracted with ethyl acetate (200 mL, 2 x). The combined organic layers were washed with water (150 mL, 3 x) and saturated aqueous NaCl solution (150 mL), dried over Na_2SO_4, filtered and concentrated in vacuo. The crude product was directly used without further purification for the next reaction.

Reduction

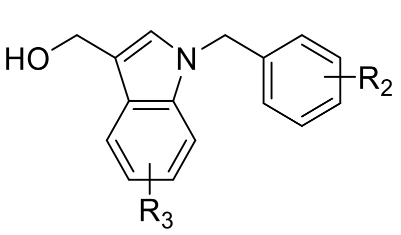

The (1-substituted-1*H*-indol-3-yl)methanol was synthesized by following a previously reported procedure.[23] To a solution of 1-substituted -1*H*-indole-3-carbaldehyde (500 mg, 1.6 mmol, 1.0 eq.) in methanol (60 mL), sodium borohydride (200 mg, 5.3 mmol, 3.2 eq.) was added portion-wise, and the reaction mixture was stirred for 1 h

at 25 °C. Water (100 mL) was added with care to the reaction, and the mixture was extracted with dichloromethane (100 mL, 2 x). The combined organic layers were dried over Na_2SO_4, filtered and concentrated in vacuo to give yields of 90–99%.

4.3.1.2 Characterization of the final compounds (intermediates were not purified)

(1-(3,4-Dichlorobenzyl)-1*H*-indol-3-yl)methanol (**7**)

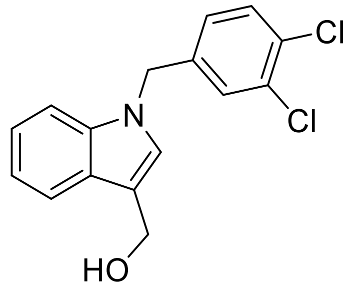

^{1}H-NMR (500 MHz, CD_3OD): δ = 7.59 (d, *J* = 7.9 Hz, 1H), 7.31 (d, *J* = 8.3 Hz, 1H), 7.18 (s, 1H), 7.17 (d, *J* = 2.3 Hz, 2H), 7.08 – 7.02 (m, 1H), 7.01 – 6.96 (m, 1H), 6.94 (dd, *J* = 8.3, 1.9 Hz, 1H), 5.25 (s, 2H), 4.71 (s, 2H). ^{13}C-NMR (126 MHz, CD_3OD): δ = 140.6, 138.1, 133.5, 132.2, 131.7, 129.9, 129.0, 128.3, 127.7, 123.1, 120.6, 120.3, 116.9, 110.8, 57.0, 49.4. HR-ESI-MS: calculated for $C_{16}H_{12}Cl_2$ [*M*-OH]$^{\cdot}$: 288.03 (^{35}Cl), 290.03 (^{37}Cl), found 288.03, 290.03.

(1-(4-Chlorobenzyl)-1*H*-indol-3-yl)methanol (**16**)

This compound has been synthesized and characterized before. Data were in accordance with literature values.[24]

(1-(3,4-Dichlorobenzyl)-5-fluoro-1*H*-indol-3-yl)methanol (**17**)

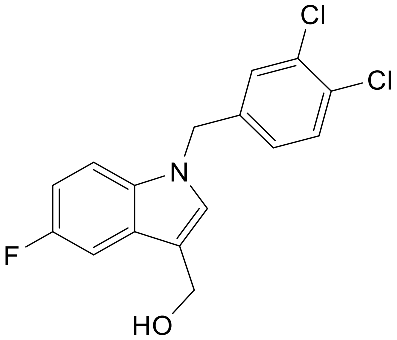

^{1}H-NMR (500 MHz, $(CD_3)_2OS$): δ = 7.57 (d, *J* = 8.3 Hz, 1H), 7.52 (m, 2H), 7.45 (dd, *J* = 4.5, 8.8 Hz, 1H), 7.36 (dd, *J* = 2.6, 9.9 Hz, 1H), 7.15 (dd, *J* = 2.1, 8.3 Hz, 1H), 6.96 (td, *J* = 2.6, 9.3 Hz, 1H), 5.39 (s, 2H), 4.89 (t, *J* = 5.5 Hz, 1H), 4.60 (d, *J* = 5. 5 Hz, 2H). ^{13}C-NMR (126 MHz, $(CD_3)_2OS$): δ = 156.9 (d, *J* = 232.05 Hz), 139.4, 132.8, 131.1, 130.9, 130.1, 129.2, 128.9, 127.6, 127.5, 116.5 (d, *J* = 4.71 Hz), 111.2 (d, *J* = 9.59 Hz), 109.6 (d, *J* = 26.03 Hz), 104.2 (d, *J* = 23.13 Hz), 55.1, 47.9. ^{19}F-NMR (470 MHz,

$(CD_3)_2OS$): δ = -124.9. HR-ESI-MS: calculated for $C_{16}H_{11}Cl_2FN^{\cdot}$ [*M*-OH]$^{\cdot}$ 306.0253 (^{35}Cl), 308.0223 (^{37}Cl), found 306.0248 (100%), 308.0215 (70%).

1-(3,4-Dichlorobenzyl)-3-(hydroxymethyl)-1*H*-indole-6-carbonitrile (**18**)

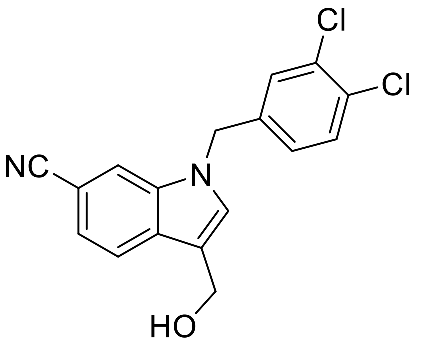

^{1}H-NMR (500 MHz, CD_3OD): δ = 7.84 (d, *J* = 8.3 Hz, 2H), 7.58 (s, 1H), 7.46 (d, *J* = 8.3 Hz, 1H), 7.43 – 7.29 (m, 1H), 7.09 (dd, *J* = 8.3, 2.1 Hz, 1H), 5.45 (s, 2H), 4.82 (s, 2H). ^{13}C-NMR (126 MHz, CD_3OD): δ = 138.3, 135.6, 132.3, 131.3, 131.0, 130.7, 130.6, 128.7, 126.5, 121.93, 120.2, 120.0, 116.7, 114.6, 104.0, 55.1, 48.1. HR-ESI-MS: calculated for $C_{17}H_{13}Cl_2N_2O$ [*M*+H]$^+$ 331.04 (^{35}Cl), 333.04 (^{37}Cl), found 313.03 (100%), 315.03, 331.04 (20%), 333.04.

(1-(3,4-Dichlorobenzyl)-7-methoxy-1*H*-indol-3-yl)methanol (**19**)

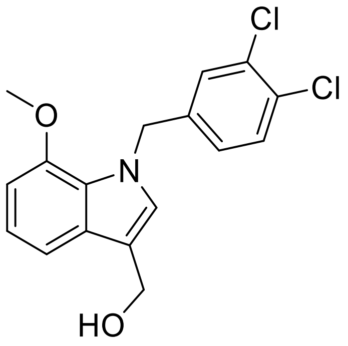

^{1}H-NMR (500 MHz, $(CD_3)_2OS$): δ = 7.55 (d, *J* = 8.3 Hz, 1H), 7.39 (d, *J* = 1.6 Hz, 1H), 7.32 (s, 1H), 7.18 (d, *J* = 7.9 Hz, 1H), 7.07 (dd, *J* = 8.3, 1.6 Hz, 1H), 6.93 (t, *J* = 7.8 Hz, 1H), 6.66 (d, *J* = 7.7 Hz, 1H), 5.53 (s, 2H), 4.84 (t, *J* = 5.4 Hz, 1H), 4.59 (d, *J* = 5.4 Hz, 2H), 3.80 (s, 3H). ^{13}C-NMR (126 MHz, $(CD_3)_2OS$): δ = 146.9, 141.2, 130.9, 130.7, 129.6, 129.5, 128.8, 127.8, 127.2, 125.3, 119.8, 116.7, 112.3, 103.1, 55.3, 55.3, 50.4. HR-ESI-MS: calculated for $C_{17}H_{14}Cl_2NO^-$ [*M*-OH]$^+$ 318.05 (^{35}Cl), 320.04 (^{37}Cl), found 318.04, 320.04.

4.3.2 Re-synthesis of hit 9 and synthesis of its active derivatives

4.3.2.1 General procedures

Claisen-decarboxylation

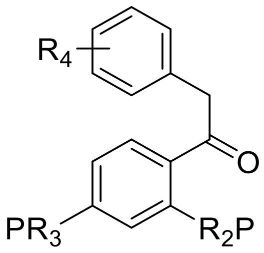

Synthesis of the protected ketone intermediate followed a previously reported procedure.[25] To a Schlenk flask, methyl benzoate (400 mg, 1.0 eq.) and phenylacetic acid (348 mg, 1.0 eq.) were added and dissolved in dry DMF (12 mL) under nitrogen. The yellow solution was cooled to –10 °C, and then sodium *bis*(trimethylsilyl)amide (2 M in THF, 5.9 mL, 4.0 eq.) was added dropwise under stirring. After full conversion of the starting material (3 h), the reaction was terminated by adding saturated NH_4Cl solution (5 mL) and concentrated in vacuo to remove DMF. Subsequently, the residue was extracted with ethyl acetate (10 mL, 3 x), and the combined organic layers were washed with brine (10 mL). The organic layer was dried over Na_2SO_4, filtered and reduced in vacuo. The obtained yellow crude was used without further purification.

Deprotection

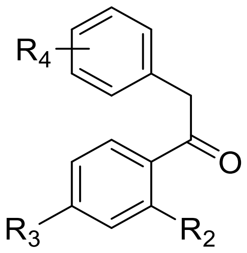

Deprotection followed a previously reported procedure.[26] To a solution of Claisen decarboxylation product (300 mg, 1.0 eq.) in dry dichloromethane (9 mL) under nitrogen, boron tribromide was added (1 M in dichloromethane, 2 mL, 12.0 eq.) under stirring at 25 °C. After five hours, a saturated Na_2CO_3-solution was added (10 mL) to the orange solution, which was extracted with dichloromethane. The organic layer was washed with water (15 mL, 2 x), then dried over Na_2SO_4, filtered and concentrated in vacuo to give yields of 44–98%.

Oximation/hydrazone reaction

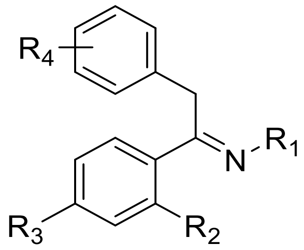

Oximation followed a previously reported procedure.[27] To a solution of de-protected product (100 mg, 0.4 mmol) in methanol (MeOH, 5 mL), potassium acetate (112 mg, 1.2 mmol, 3.0 eq.) and hydroxylamine chlorohydrate (40 mg, 0.6 mmol, 1.5 eq.) were subsequently added under stirring. The light-yellow suspension was refluxed for two hours. Subsequently, water (5 mL) was added to the mixture. The organic layer was washed with saturated aqueous NaCl solution (3 mL), dried over Na_2SO_4, filtered and concentrated in vacuo. The crude product was further purified by silica gel column chromatography (*n*-hexane/EtOAc, 8:2, v/v) to give yields of 32–73% of the *E*-isomer as main product.

4.3.2.2 Characterization of intermediates and final compounds (intermediates were not purified)

4-[(4-chlorobenzyl)(hydroxyimino)methyl]benzene-1,3-diol (**9**)

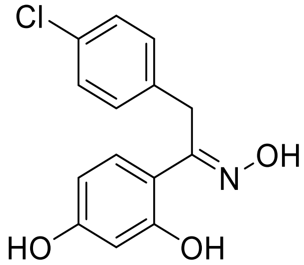

^{1}H-NMR (400 MHz, CD_3OD): δ = 7.42 (s, 1H), 7.40–7.38 (d, *J* = 8.0 Hz, 1H), 7.21 (m, 3H), 6.29–6.26 (m, 2H), 4.20 (s, 2H). ^{13}C-NMR (101 MHz, CD_3OD): δ = 160.5, 160.1, 159.8, 136.6, 132.79, 130.8, 129.9, 129.3, 111.4, 107.8, 104.1, 30.4. HR-ESI-MS: calculated for $C_{14}H_{13}ClNO_3$ [M+H]$^+$: 278.06, found: 278.06.

(*E*)-4-(2-(4-Chlorophenyl)-1-hydrazineylideneethyl)benzene-1,3-diol (**21**)

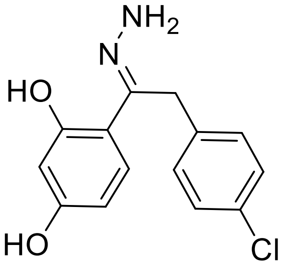

^{1}H-NMR (500 MHz, CD_3OD): δ = 7.32 – 7.26 (m, 2H), 7.22 (d, *J* = 8.4 Hz, 2H), 7.17 (d, *J* = 8.7 Hz, 1H), 6.27 (d, *J* = 2.5 Hz, 1H), 6.23 (dd, *J* = 8.7, 2.5 Hz, 1H), 4.09 (s, 2H). ^{13}C-NMR (126 MHz, CD_3OD): δ = 161.6, 160.1, 154.5, 136.1, 133.4, 130.9, 129.9, 129.4, 113.7, 107.5, 104.2, 30.8. HR-ESI-MS: calculated for $C_{14}H_{14}ClN_2O_2$ $[M+H]^+$ 277.07 (^{35}Cl), 279.07 (^{37}Cl), found 277.07, 279.07.

(*E*)-2-(4-Chlorophenyl)-1-(2-hydroxyphenyl)ethan-1-one oxime (**23**)

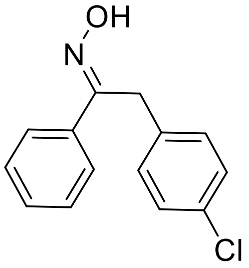

^{1}H-NMR (400 MHz, $CDCl_3$): δ = 7.64 (dd, *J* = 6.5, 2.9 Hz, 2H), 7.48–7.34 (m, 3H), 7.25 (q, *J* = 8.6 Hz, 4H), 4.23 (s, 2H). ^{13}C-NMR (101 MHz, $CDCl_3$): δ = 157.3, 135.4, 135.0, 132.3, 130.1, 129.7, 128.9, 128.8, 126.6, 31.8. HR-ESI-MS: calculated for $C_{14}H_{13}ClNO$ $[M+H]^+$ 246.07 (^{35}Cl), 248.07 (^{37}Cl), found 246.07, 248.07.

(*E*)-2-(4-Chlorophenyl)-1-(2-hydroxy-4-methoxyphenyl)ethan-1-one oxime (**24**)

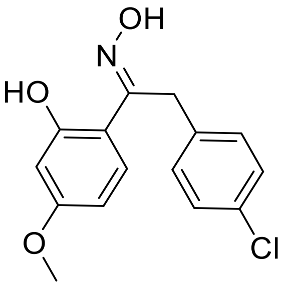

^{1}H-NMR (500 MHz, CD_3OD): δ = 7.31 (d, *J* = 8.7 Hz, 1H), 7.26 (s, 4H), 6.42 – 6.37 (m, 2H), 4.22 (s, 2H), 3.75 (s, 3H). ^{13}C-NMR (126 MHz, CD_3OD): δ = 162.9, 161.0, 160.1, 137.2, 133.1, 131.2, 130.1, 129.6, 112.5, 106.6, 102.7, 55.7, 30.5. HR-ESI-MS: calculated for $C_{15}H_{15}ClNO_3$ $[M+H]^+$ 292.07 (^{35}Cl), 294.07 (^{37}Cl), found 292.07, 294.07.

(*E*)-1-(4-Amino-2-hydroxyphenyl)-2-(4-chlorophenyl)ethan-1-one oxime (**25**)

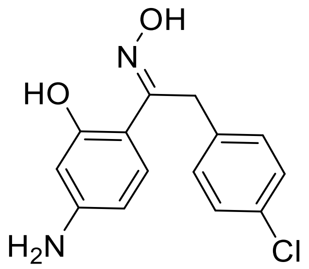

^{1}H-NMR (400 MHz, CD_3OD): δ = 7.18 (s, 4H), 7.08 (d, *J* = 8.5 Hz, 1H), 6.36–6.00 (m, 2H), 4.12 (s, 2H). ^{13}C-NMR (101 MHz, CD_3OD): δ = 159.2, 158.9, 149.2, 135.5, 131.8, 129.7, 128.8, 128.4, 108.9, 106.7, 102.2, 29.6. HR-ESI-MS: calculated for $C_{14}H_{14}ClN_2O_2$ [*M*+H]$^+$ 277.07 (^{35}Cl), 279.07 (^{37}Cl), found 277.07, 279.07.

4.3.3 Re-synthesis of hit 10 and its active derivatives

4.3.3.1 General procedures

Synthesis of α-bromoketones

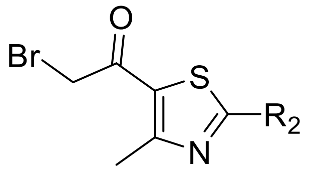

Synthesis of α-bromoketones followed a previously reported procedure.[28] 5-Acetyl-2-substituted-4-methylthiazole (6.4 mmol) was dissolved in 48% HBr solution in water (20 mL) and the mixture was heated to 60 °C. Bromine (260 µL, 5.1 mmol, 0.8 eq.) in dioxane (20 mL) was added dropwise over 1 h. The reaction mixture was stirred at 60 °C for 4 h before the mixture was concentrated in vacuo and co-evaporated with toluene (5 mL, 3 x). The remaining residue was diluted with water (5 mL) and basified with a saturated aqueous $NaHCO_3$ solution (5 mL). Then, the aqueous layer was extracted with EtOAc (10 mL, 3 x) and the combined organic layers were washed with saturated aqueous NaCl Solution (20 mL), dried over $MgSO_4$, filtered and concentrated. Silica gel column chromatography (dichloromethane/MeOH with main separation step at 5% MeOH) gave a mixture of starting material and product (28–55%) that could not be separated further. The mixture was directly used in the next reaction.

Synthesis of the aminothiazole derivatives

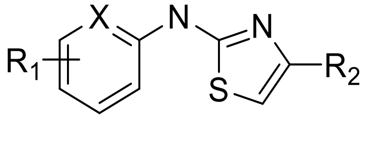

Synthesis of aminothiazoles followed a previously reported procedure.[28] The impure mixture of α-bromoketones (0.35 mmol) and substituted thiourea (0.33 mmol) were dissolved in absolute EtOH. Then, DIPEA (0.39 mmol, 68 µL, 1.1 eq.) was added and the mixture stirred for 3 days. Thin layer chromatography analysis showed that the product spot turned red after irradiation with UV-light. The solvent was evaporated, and the residue diluted with EtOAc (5 mL) and filtered over Celite. The filtrate was washed with water (2 mL, 3 x) and saturated aqueous NaCl solution (2 mL, 2 x), dried over $MgSO_4$, filtered and concentrated in vacuo. Purification by HPLC (water with 0.5% formic acid/MeOH) yielded the product to give yields of 37–80%.

4.3.3.2 Characterization of the final compounds (intermediates were not purified)

4-(2-amino-4-methylthiazol-5-yl)-*N*-(4-(trifluoromethyl)phenyl)thiazol-2-amine (**10**)

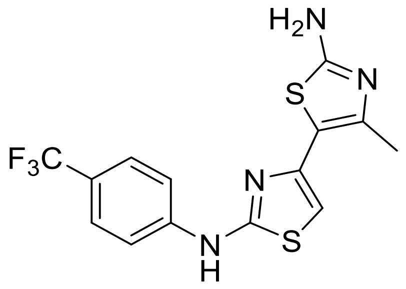

^{1}H-NMR (500 MHz, $(CD_3)_2OS$): δ = 10.67 (s, 1H), 8.15 (s, 1H, formic acid), 7.83 (d, *J* = 8.6 Hz, 2H), 7.66 (d, *J* = 8.6 Hz, 2H), 7.00 (s, 2H), 6.71 (s, 1H), 2.34 (s, 3H). ^{13}C-NMR (126 MHz, $(CD_3)_2OS$): δ = 166.3, 163.6 (formic acid), 162.1, 145.1, 144.8, 144.1, 126.6 (q, J_{CF} = 3.6 Hz), 125.1 (q, J_{CF} = 270.9 Hz), 121.4 (q, J_{CF} = 32.0 Hz), 116.9, 114.4, 101.6, 17.5. ^{19}F NMR (470 MHz, $(CD_3)_2OS$): δ = –59.8 (s). HR-ESI-MS: calculated for $C_{14}H_{12}F_3N_4S_2$ $[M+H]^+$: 357.04, found 357.04.

4'-Methyl-N^2-(2-(trifluoromethyl)phenyl)-[5,5'-bithiazole]-2,2'-diamine (**27**)

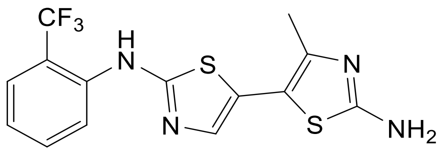

^{1}H-NMR (500 MHz, CD_3OD): δ = 8.10 (d, *J* = 8.3 Hz, 1H), 7.68 (d, *J* = 7.8 Hz, 1H), 7.61 (t, *J* = 7.8 Hz, 1H), 7.27 (t, *J* = 7.7 Hz, 1H), 6.58 (s, 1H), 2.34 (s, 3H). ^{13}C-NMR (126 MHz, CD_3OD): δ = 169.1, 166.5, 144.6, 144.3, 140.0, 134.1, 127.6 (q, *J* = 5.44 Hz), 125.7, 125.4 (q, *J* = 272.28 Hz), 125.1, 122.6 (q, *J* = 29.43 Hz), 116.2, 103.5, 16.5. ^{19}F-NMR (470 MHz, $(CD_3)_2OS$): δ (ppm) = -59.2. HR-ESI-MS: calculated for $C_{14}H_{12}F_3N_4S_2$ $[M+H]^+$ 357.04, found 357.04.

4'-Methyl-*N*[2]-(3-(trifluoromethyl)phenyl)-[4,5'-bithiazole]-2,2'-diamine (**28**)

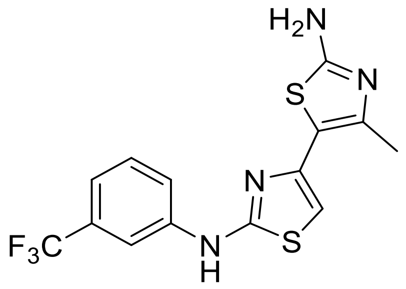

^{1}H-NMR (500 MHz, $(CD_3)_2OS$): δ = 10.61 (s, 1H), 8.42 (s, 1H), 7.63 (d, *J* = 8.2 Hz, 1H), 7.52 (t, *J* = 7.9 Hz, 1H), 7.27 (d, *J* = 7.6 Hz, 1H), 7.01 (s, 2H), 6.67 (s, 1H), 2.34 (s, 3H). ^{13}C-NMR (126 MHz, $(CD_3)_2OS$): δ = 165.8, 161.8, 144.6, 143.6, 141.7, 129.9, 129.7 (q, J_{CF} = 31.3 Hz), 123.2 (q, J_{CF} = 272.5 Hz), 120.3, 117.2 (q, J_{CF} = 4.4 Hz), 113.9, 112.6, 100.7, 17.0. ^{19}F-NMR (470 MHz, $(CD_3)_2OS$): δ = -61.31. HR-ESI-MS: calculated for $C_{14}H_{12}F_3N_4S_2$ $[M+H]^+$ 357.05, found 357.04.

4-(2-((3-(Trifluoromethyl)phenyl)amino)thiazol-5-yl)benzene-1,3-diol (**29**)

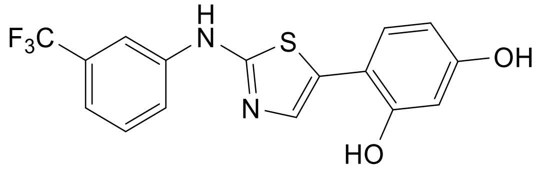

^{1}H-NMR (500 MHz, $(CD_3)_2OS$): δ = 10.63 (s, 1H), 10.47 (s, 1H), 9.50 (s, 1H), 8.18 (s, 1H), 7.76 (m, 2H), 7.57 (t, *J* = 7.9 Hz, 1H), 7.30 (d, *J* = 7.6 Hz, 1H), 7.25 (s, 1H), 6.37 (d, *J* = 2.07 Hz, 1H), 6.31 (dd, *J* = 2.09, 8.52 Hz, 1H). ^{13}C-NMR (126 MHz, $(CD_3)_2OS$): δ = 161.5, 158.1, 156.3, 147.4, 141.7, 130.3, 129.8 (q, *J* = 31.51 Hz), 129.0, 124.3 (q, *J* = 272.36 Hz), 120.4, 117.4 (q, *J* = 3.70 Hz), 112.8 (q, *J* = 4.13 Hz), 112.0, 107.0, 102.9, 102.4. ^{19}F-NMR (470 MHz, $(CD_3)_2OS$): δ = -61.4. HR-ESI-MS: calculated for $C_{16}H_{12}F_3N_2O_2S$ $[M+H]^+$ 353.06, found 353.06.

N[2]-(5-Chloropyridin-2-yl)-4'-methyl-[4,5'-bithiazole]-2,2'-diamine (**30**)

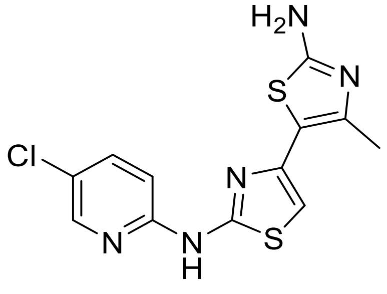

^{1}H-NMR (500 MHz, $(CD_3)_2OS$): δ = 11.51 (s, 1H), 8.34 (d, *J* = 2.6 Hz, 1H), 7.80 (dd, *J* = 8.9, 2.6 Hz, 1H), 7.26 – 7.05 (m, 1H), 6.95 (s, 2H), 6.78 (s, 1H), 2.31 (s, 3H). ^{13}C-NMR (126 MHz, $(CD_3)_2OS$): δ = 165.8, 158.5, 150.4, 144.6, 144.1, 142.5, 137.9, 122.1,

114.3, 112.3, 103.6, 17.0. HR-ESI-MS: calculated for $C_{12}H_{11}ClN_5S_2$ [*M*+H]$^+$ 324.01 (^{35}Cl), 326.01 (^{37}Cl), found 324.01, 326.01.

5. References

(1) CORINA. Molecular Networks GmbH – Computerchemie:Neumeyerstraße 22-34 90411 Nürnberg, Germany **2021**.

(2) Awale, M.; Reymond, J.-L. Atom pair 2D-fingerprints perceive 3D-molecular shape and pharmacophores for very fast virtual screening of ZINC and GDB-17. *J. Chem. Inf. Model.* **2014**, *54* (7), 1892–1907.

(3) Simonin, C.; Awale, M.; Brand, M.; van Deursen, R.; Schwartz, J.; Fine, M.; Kovacs, G.; Häfliger, P.; Gyimesi, G.; Sithampari, A.; Charles, R.-P.; Hediger, M. A.; Reymond, J.-L. Optimization of TRPV6 Calcium Channel Inhibitors Using a 3D Ligand-Based Virtual Screening Method. *Int. Ed. Angew. Chem.* **2015**, *54* (49), 14748–14752.

(4) Xiang, S.; Usunow, G.; Lange, G.; Busch, M.; Tong, L. Crystal structure of 1-deoxy-D-xylulose 5-phosphate synthase, a crucial enzyme for isoprenoids biosynthesis. *J. Biol. Chem.* **2007**, *282* (4), 2676–2682.

(5) LeadIT version 2.3.2. BioSolveIT GmbH:An der Ziegelei 79, 53757 Sankt Augustin **2021**.

(6) Masini, T.; Lacy, B.; Monjas, L.; Hawksley, D.; Voogd, A. R. de; Illarionov, B.; Iqbal, A.; Leeper, F. J.; Fischer, M.; Kontoyianni, M.; Hirsch, A. K. H. Validation of a homology model of Mycobacterium tuberculosis DXS: rationalization of observed activities of thiamine derivatives as potent inhibitors of two orthologues of DXS. *Org. Biomol. Chem.* **2015**, *13* (46), 11263–11277.

(7) Molecular Operating Environment. 2020.09 Chemical Computing Group ULC:1010 Sherbooke St. West, Suite #910, Montreal, OC, Canada, H3A 2R7 **2022**.

(8) Masini, T.; Pilger, J.; Kroezen, B. S.; Illarionov, B.; Lottmann, P.; Fischer, M.; Griesinger, C.; Hirsch, A. K. H. De novo fragment-based design of inhibitors of DXS guided by spin-diffusion-based NMR spectroscopy. *Chem. Sci.* **2014**, *5* (9), 3543–3551.

(9) Marcozzi, A.; Masini, T.; Di Zhu; Pesce, D.; Illarionov, B.; Fischer, M.; Herrmann, A.; Hirsch, A. K. H. Phage Display on the Anti-infective Target 1-Deoxy-d-xylulose-5-phosphate Synthase Leads to an Acceptor-Substrate Competitive Peptidic Inhibitor. *ChemBioChem* **2018**, *19* (1), 58–65.

(10) Brammer, L. A.; Meyers, C. F. Revealing substrate promiscuity of 1-deoxy-D-xylulose 5-phosphate synthase. *Organic letters* **2009**, *11* (20), 4748–4751.

(11) Handa, S.; Dempsey, D. R.; Ramamoorthy, D.; Cook, N.; Guida, W. C.; Spradling, T. J.; White, J. K.; Woodcock, H. L.; Merkler, D. J. Mechanistic studies of 1-deoxy-D-xylulose-5-phosphate synthase from Deinococcus radiodurans. *Biochemistry & molecular biology journal* **2018**, *4* (1).

(12) Jumde, R. P.; Guardigni, M.; Gierse, R. M.; Alhayek, A.; Di Zhu; Hamid, Z.; Johannsen, S.; Elgaher, W. A. M.; Neusens, P. J.; Nehls, C.; Haupenthal, J.; Reiling, N.; Hirsch, A. K. H. Hit-optimization using target-directed dynamic combinatorial chemistry: development of inhibitors of the anti-infective target 1-deoxy-d-xylulose-5-phosphate synthase. *Chem. Sci.* **2021**, *12* (22), 7775–7785.

(13) P. Kuzmic. Analytical biochemistry. (Biokin, http://www.biokin.com/dynafit/) **1996** (237), 260–273.

(14) Bartee, D.; Freel Meyers, C. L. Targeting the unique mechanism of bacterial 1-deoxy-d-xylose-5-phosphate synthase. *Biochem.* **2018**, *57* (29), 4349–4356.
(15) Smith, J. M.; Warrington, N. V.; Vierling, R. J.; Kuhn, M. L.; Anderson, W. F.; Koppisch, A. T.; Freel Meyers, C. L. Targeting DXP synthase in human pathogens: enzyme inhibition and antimicrobial activity of butylacetylphosphonate. *J. Antibiot.* **2014**, *67*, 77-83.
(16) Sahner, J. H.; Groh, M.; Negri, M.; Haupenthal, J.; Hartmann, R. W. Novel small molecule inhibitors targeting the "switch region" of bacterial RNAP: structure-based optimization of a virtual screening hit. *Eur. J. Med. Chem.* **2013**, *65*, 223–231.
(17) van Klingeren, B.; Dessens-Kroon, M.; van der Laan, T.; Kremer, K.; van Soolingen, D. Drug susceptibility testing of Mycobacterium tuberculosis complex by use of a high-throughput, reproducible, absolute concentration method. *J. Clin. Microbiol.* **2007**, *45* (8), 2662–2668.
(18) Haupenthal, J.; Baehr, C.; Zeuzem, S.; Piiper, A. RNAse A-like enzymes in serum inhibit the anti-neoplastic activity of siRNA targeting polo-like kinase 1. *Int. J. Cancer* **2007**, *121* (1), 206–210.
(19) Chmielewski, M. J.; Buhler, E.; Candau, J.; Lehn, J.-M. Multivalency by self-assembly: binding of concanavalin A to metallosupramolecular architectures decorated with multiple carbohydrate groups. *Chemistry* **2014**, *20* (23), 6960–6977.
(20) Zhu, T.; Cao, S.; Su, P.-C.; Patel, R.; Shah, D.; Chokshi, H. B.; Szukala, R.; Johnson, M. E.; Hevener, K. E. Hit identification and optimization in virtual screening: practical recommendations based on a critical literature analysis. *J. Med. Chem.* **2013**, *56* (17), 6560–6572.
(21) McGovern, S. L.; Helfand, B. T.; Feng, B.; Shoichet, B. K. A specific mechanism of nonspecific inhibition. *J. Med. Chem.* **2003**, *46* (20), 4265–4272.
(22) Meguellati, A.; Ahmed-Belkacem, A.; Yi, W.; Haudecoeur, R.; Crouillère, M.; Brillet, R.; Pawlotsky, J.-M.; Boumendjel, A.; Peuchmaur, M. B-ring modified aurones as promising allosteric inhibitors of hepatitis C virus RNA-dependent RNA polymerase. *Eur. J. Med. Chem.* **2014**, *80*, 579–592.
(23) Giraud, F.; Loge, C.; Pagniez, F.; Crepin, D.; Barres, S.; Picot, C.; Le Pape, P.; Le Borgne, M. Design, synthesis and evaluation of 3-(imidazol- 1-ylmethyl)indoles as antileishmanial agents. Part II. *J. Enzyme Inhib. Med. Chem.* **2009**, *24* (5), 1067–1075.
(24) Wu, S.; Wang, L.; Guo, W.; Liu, X.; Liu, J.; Wei, X.; Fang, B. Analogues and derivatives of oncrasin-1, a novel inhibitor of the C-terminal domain of RNA polymerase II and their antitumor activities. *J. Med. Chem.* **2011**, *54* (8), 2668–2679.
(25) Wu, G.; Yin, W.; Shen, H. C.; Huang, Y. One-pot synthesis of useful heterocycles in medicinal chemistry using a cascade strategy. *Green Chem.* **2012**, *14* (3), 580–585.
(26) Snyder, S. A.; Zografos, A. L.; Lin, Y. Total synthesis of resveratrol-based natural products: a chemoselective solution. *Angew. Chem. Int. Ed Engl.* **2007**, *46* (43), 8186–8191.
(27) Luo, Y.; Song, R.; Li, Y.; Zhang, S.; Liu, Z.-J.; Fu, J.; Zhu, H.-L. Design, synthesis, and biological evaluation of chalcone oxime derivatives as potential

immunosuppressive agents. *Bioorganic & medicinal chemistry letters* **2012**, *22* (9), 3039–3043.

(28) Azzali, E.; Machado, D.; Kaushik, A.; Vacondio, F.; Flisi, S.; Cabassi, C. S.; Lamichhane, G.; Viveiros, M.; Costantino, G.; Pieroni, M. Substituted N-Phenyl-5-(2-(phenylamino)thiazol-4-yl)isoxazole-3-carboxamides Are Valuable Antitubercular Candidates that Evade Innate Efflux Machinery. *J. Med. Chem.* **2017**, *60* (16), 7108–7122.
