# Supplementary material for: Discovery of novel drug-like antitubercular hits targeting the MEP pathway enzyme DXPS by strategic application of ligand-based virtual screening
Source: Chem Sci. 2022 Aug 8;13(36):10686–98. doi: 10.1039/d2sc02371g (PMC9491098; doi:10.1039/d2sc02371g)
Supplement: SC-013-D2SC02371G-s003 [file SC-013-D2SC02371G-s003.pdf]

Electronic Supplementary Material (ESI) for Chemical Science.
This journal is © The Royal Society of Chemistry 2022

Supplementary Spectra

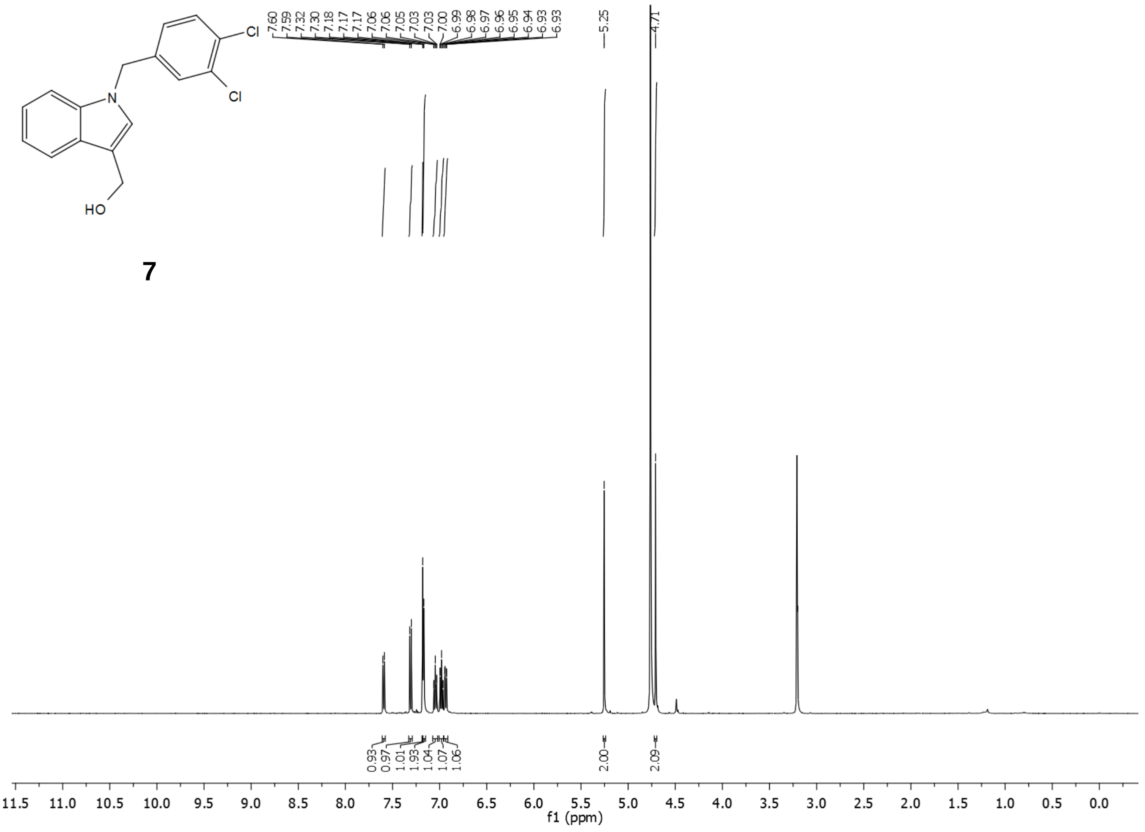

Spectrum 1: ^{1}H NMR of indole **7**.

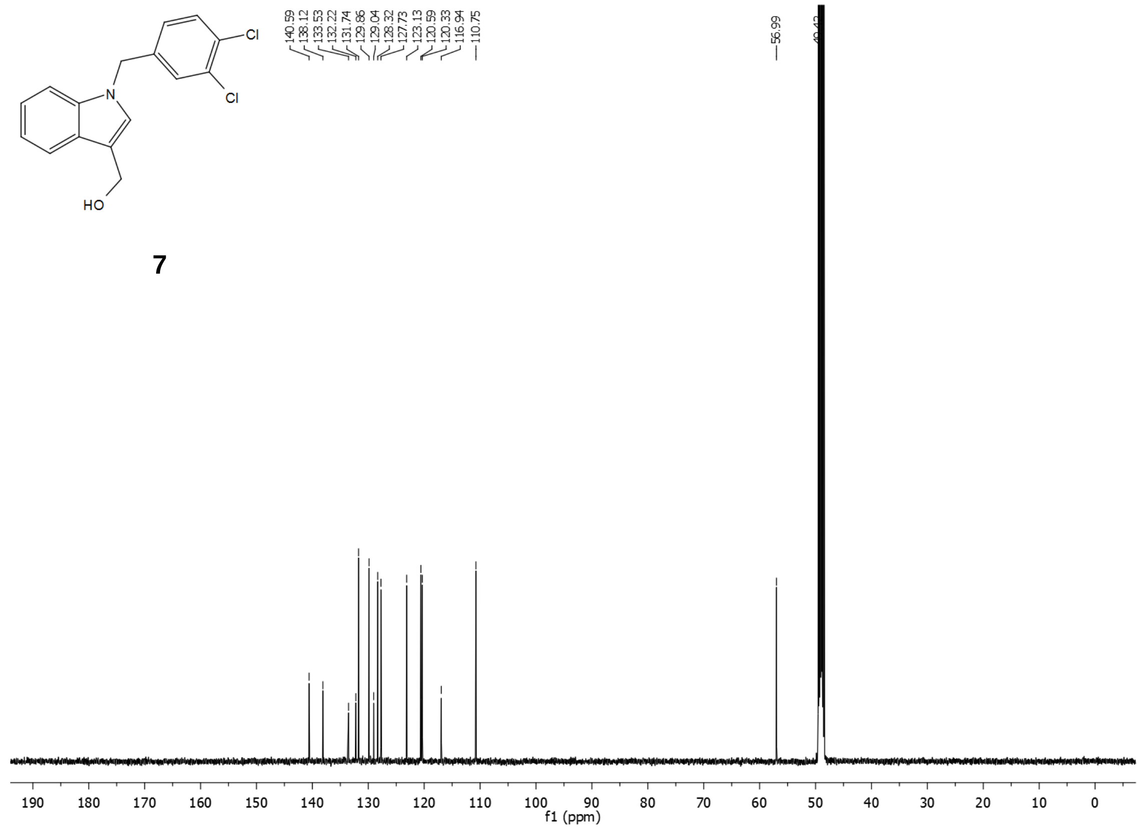

Spectrum 2: ^{13}C NMR of indole **7**.

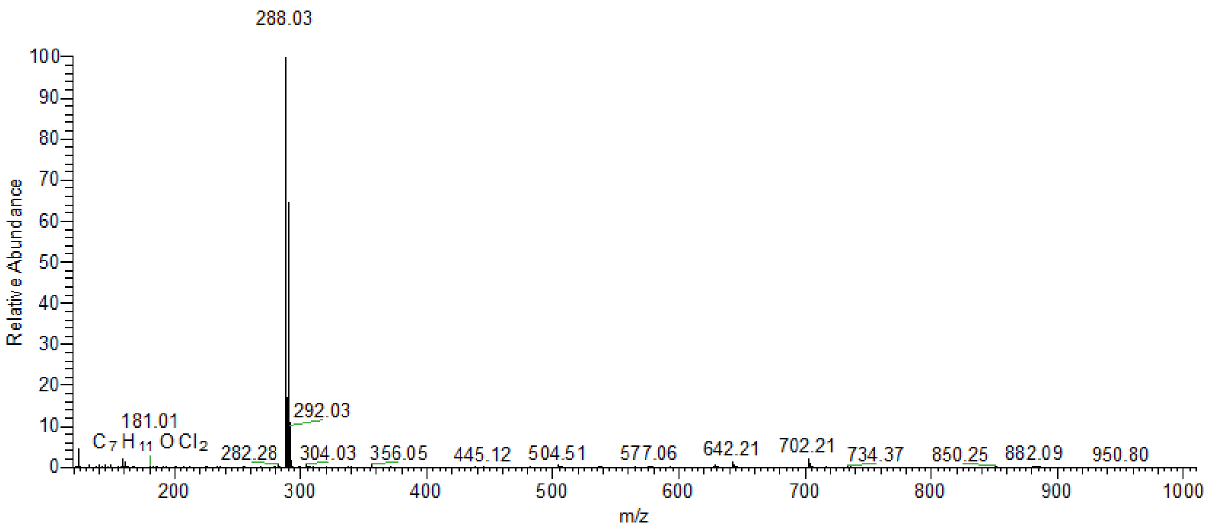

Spectrum 3: HR-ESI-MS spectrum of indole **7**.

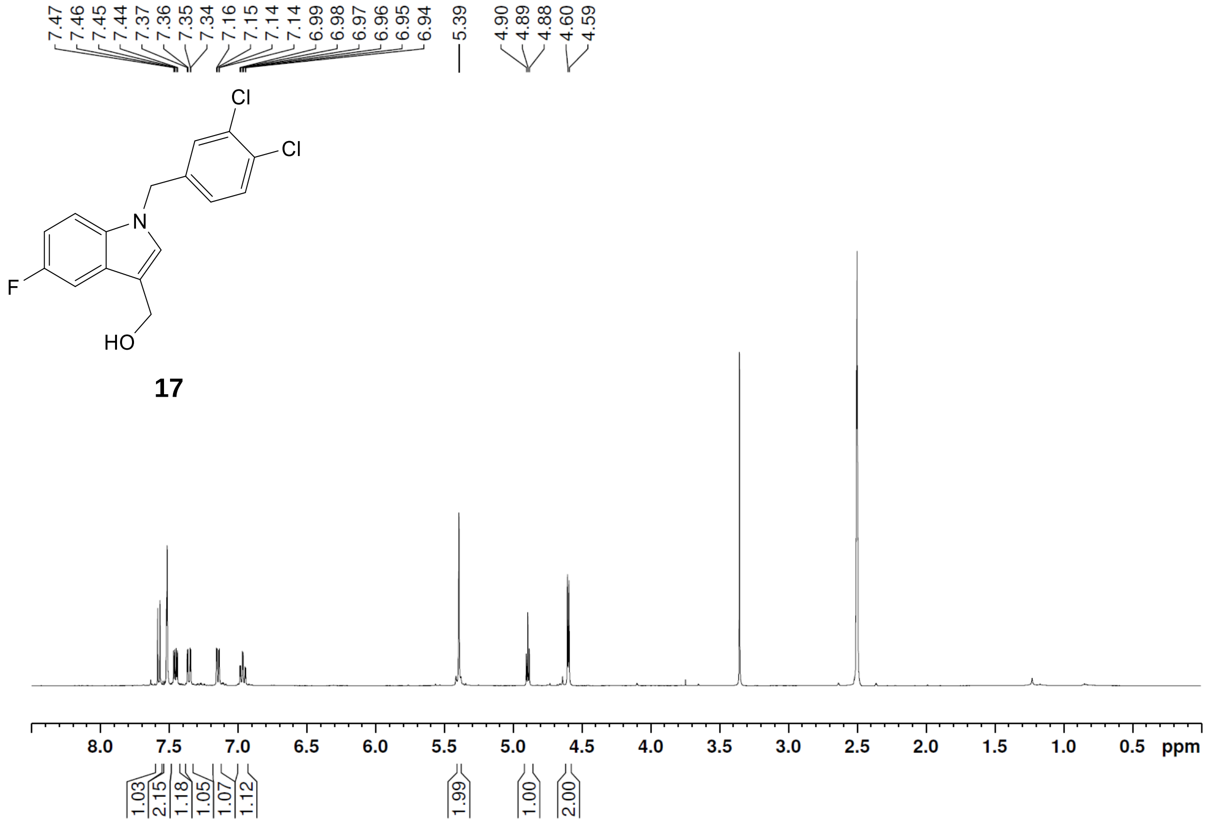

Spectrum 4: ^{1}H NMR of indole **17**.

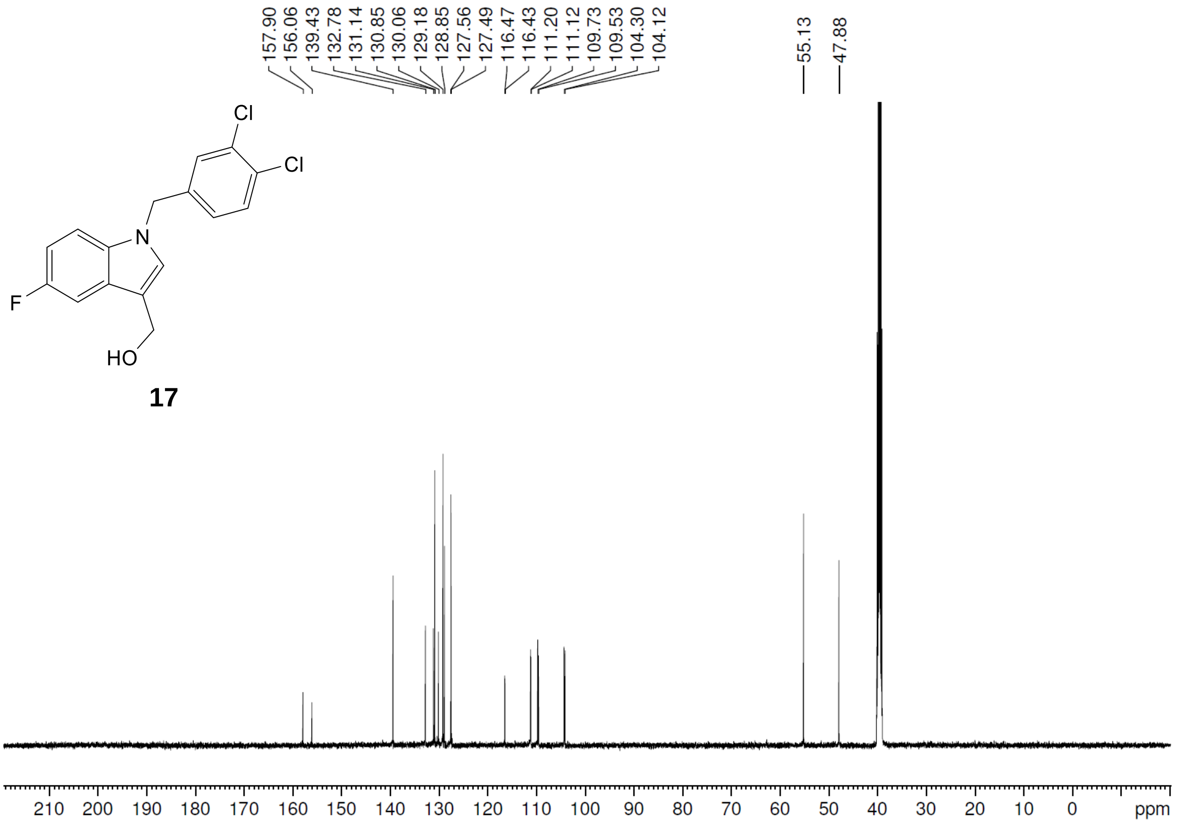

Spectrum 5: ^{13}C NMR of indole **17**.

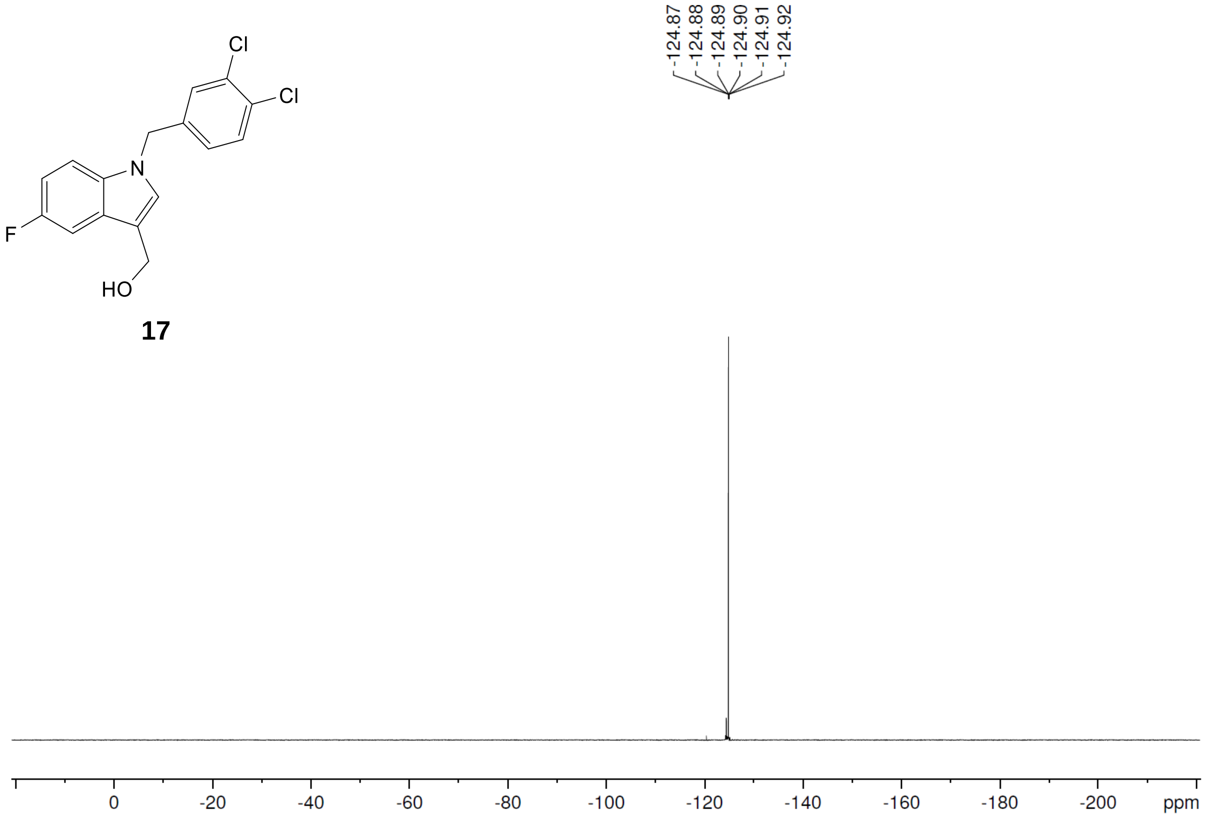

Spectrum 6: ^{19}F NMR of indole **17**.

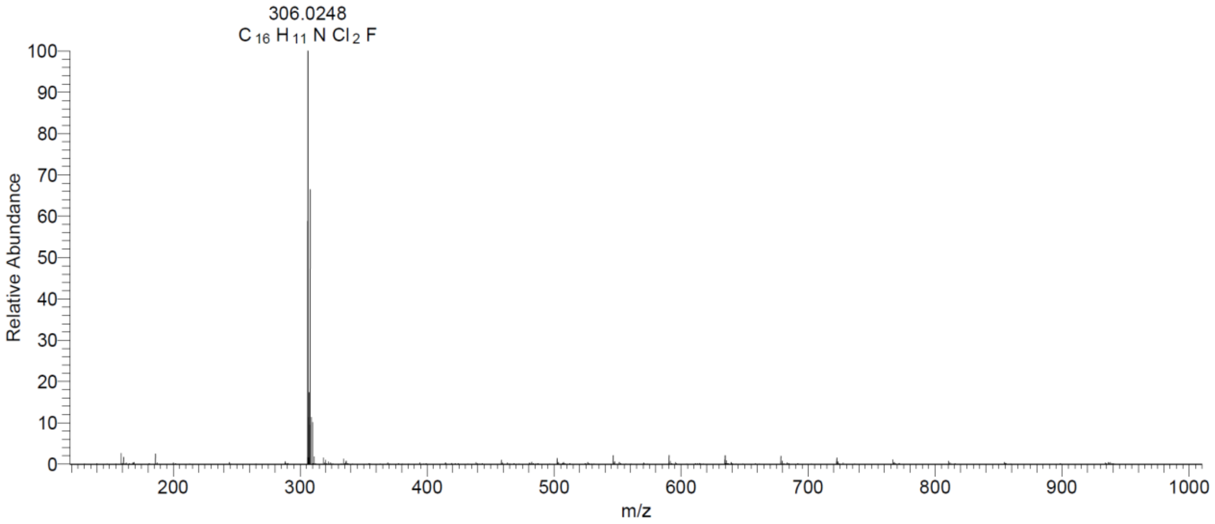

Spectrum 7: HR-ESI-MS spectrum of indole **17**.

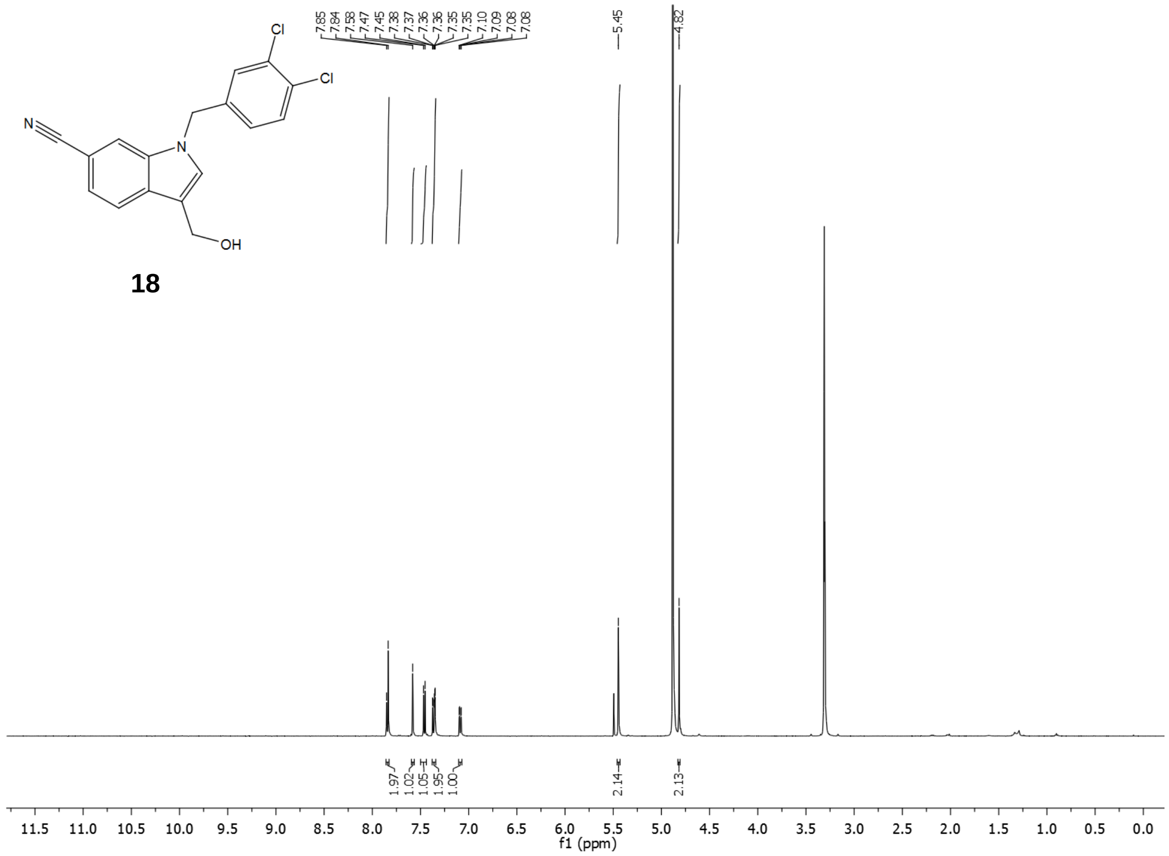

Spectrum 8: ^{1}H NMR of indole **18**.

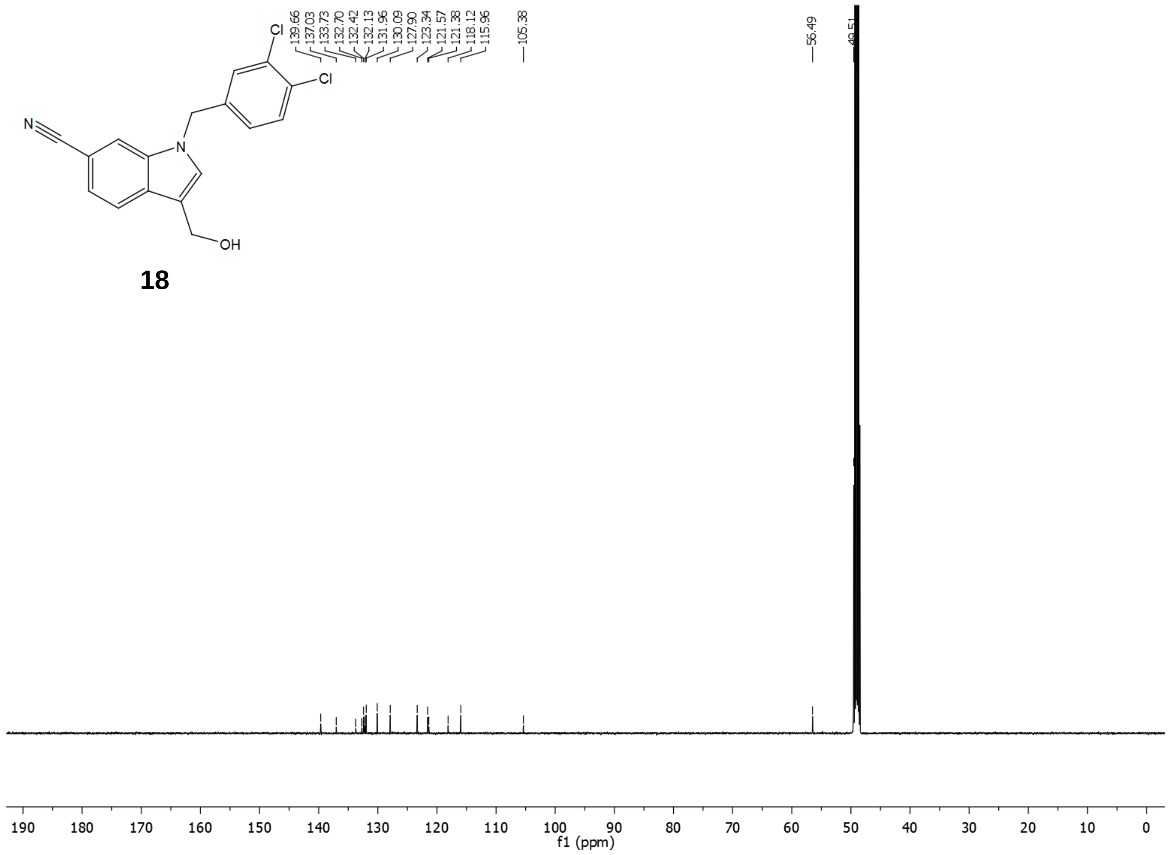

Spectrum 9: ^{13}C NMR of indole **18**.

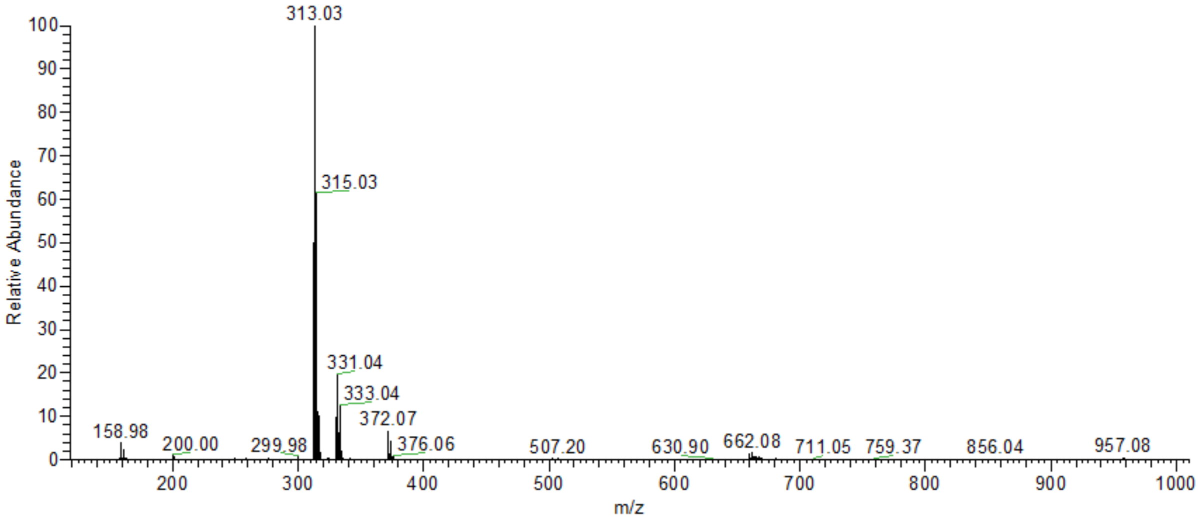

Spectrum 10: HR-ESI-MS spectrum of indole **18**.

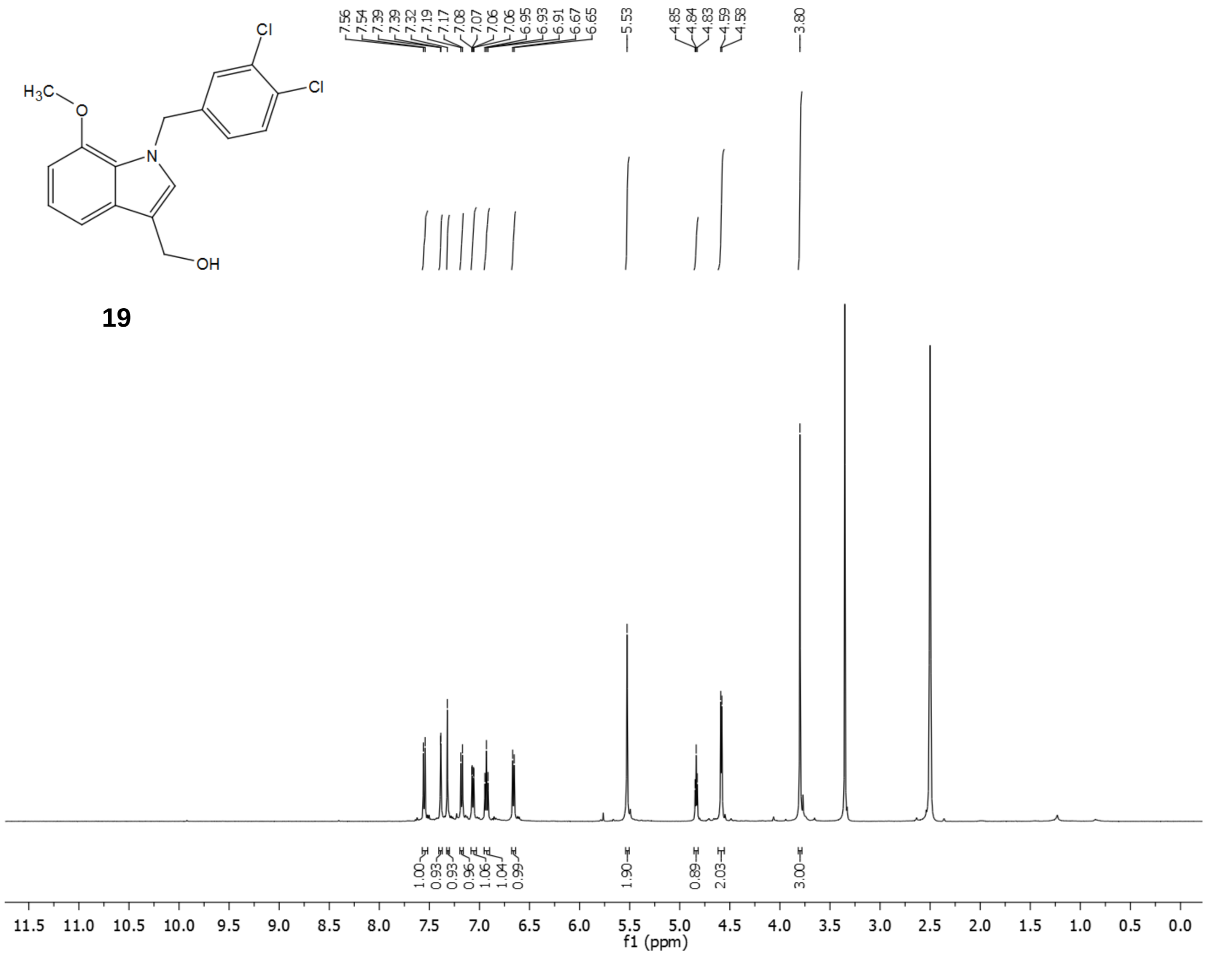

Spectrum 11: ^{1}H NMR of indole **19**.

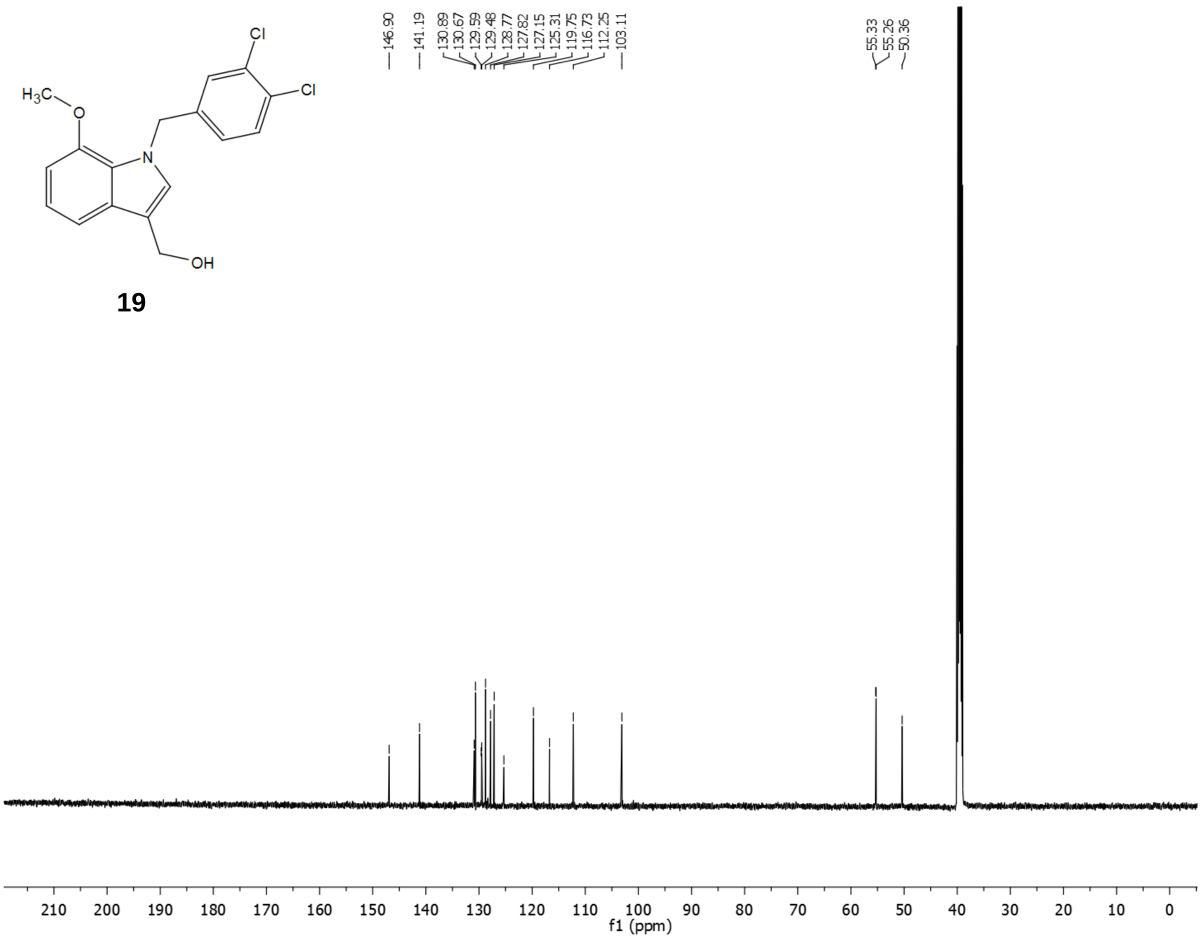

Spectrum 12: ^{13}C NMR of indole **19**.

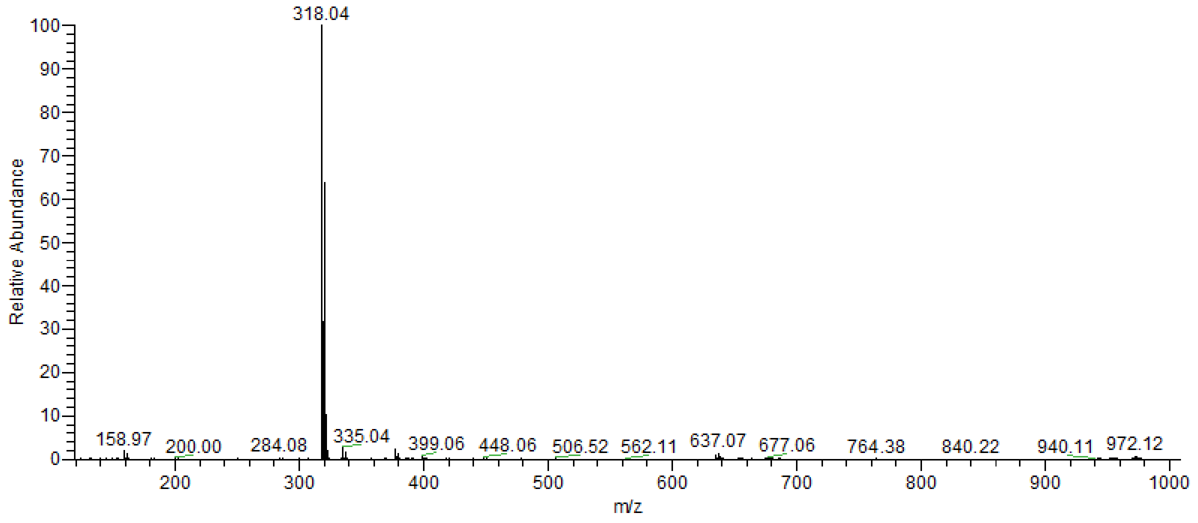

Spectrum 13: HR-ESI-MS spectrum of indole **19**.

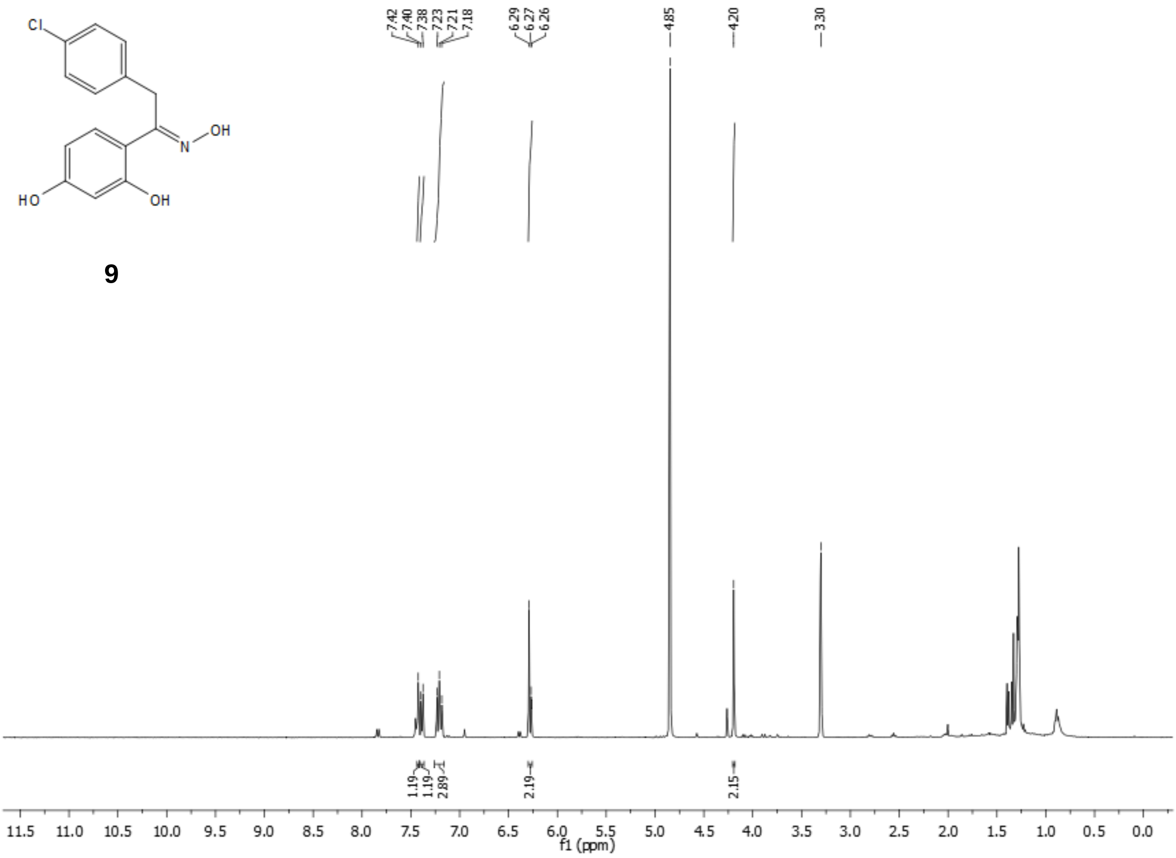

Spectrum 14: ^{1}H NMR of oxime **9**.

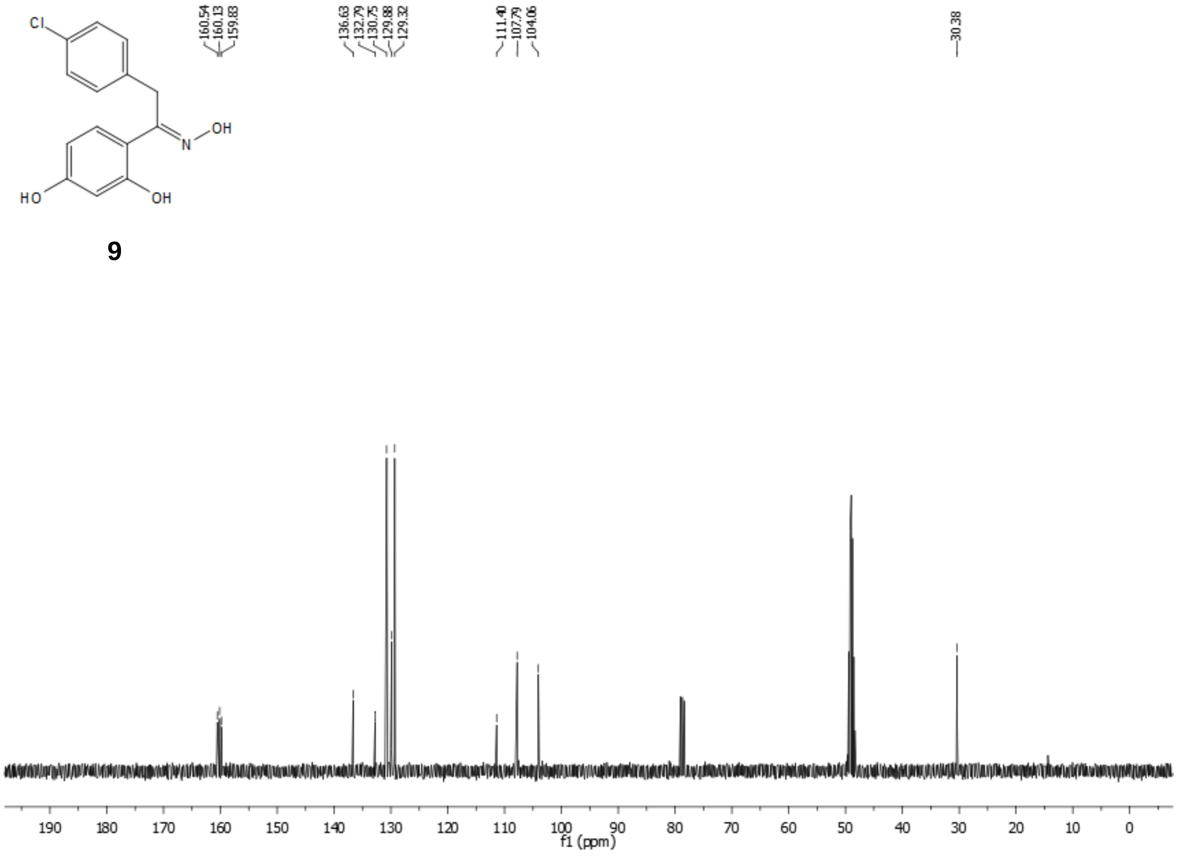

Spectrum 15: ^{13}C NMR of oxime **9**.

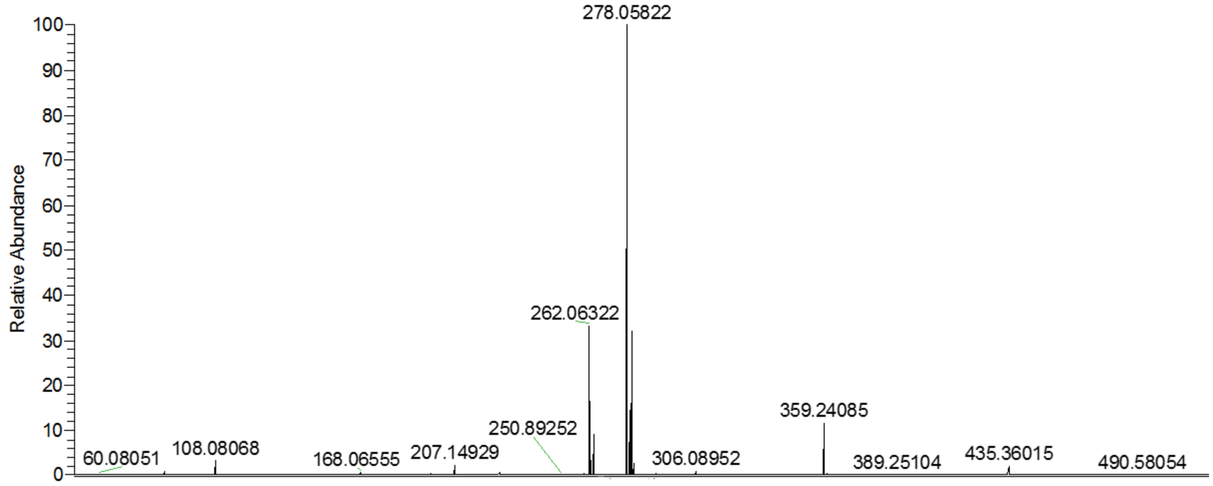

Spectrum 16: HR-ESI-MS spectrum of oxime **9**.

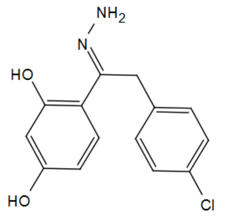

21

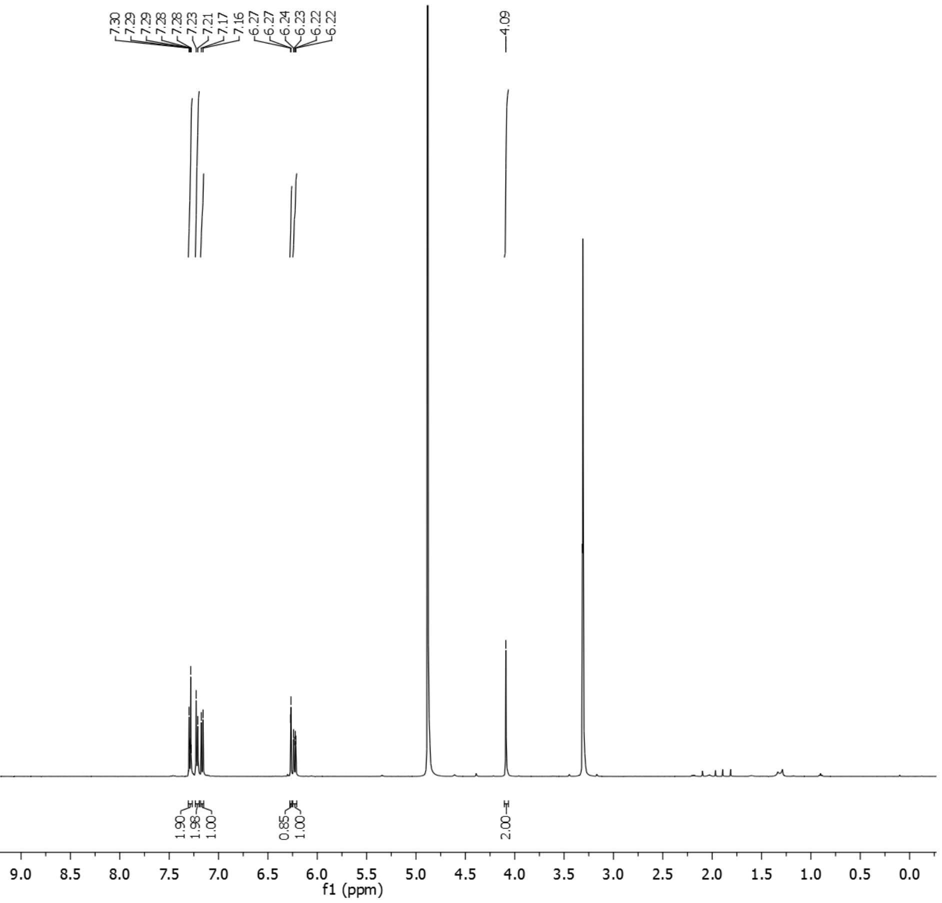

Spectrum 17: ^{1}H NMR of oxime **21**.

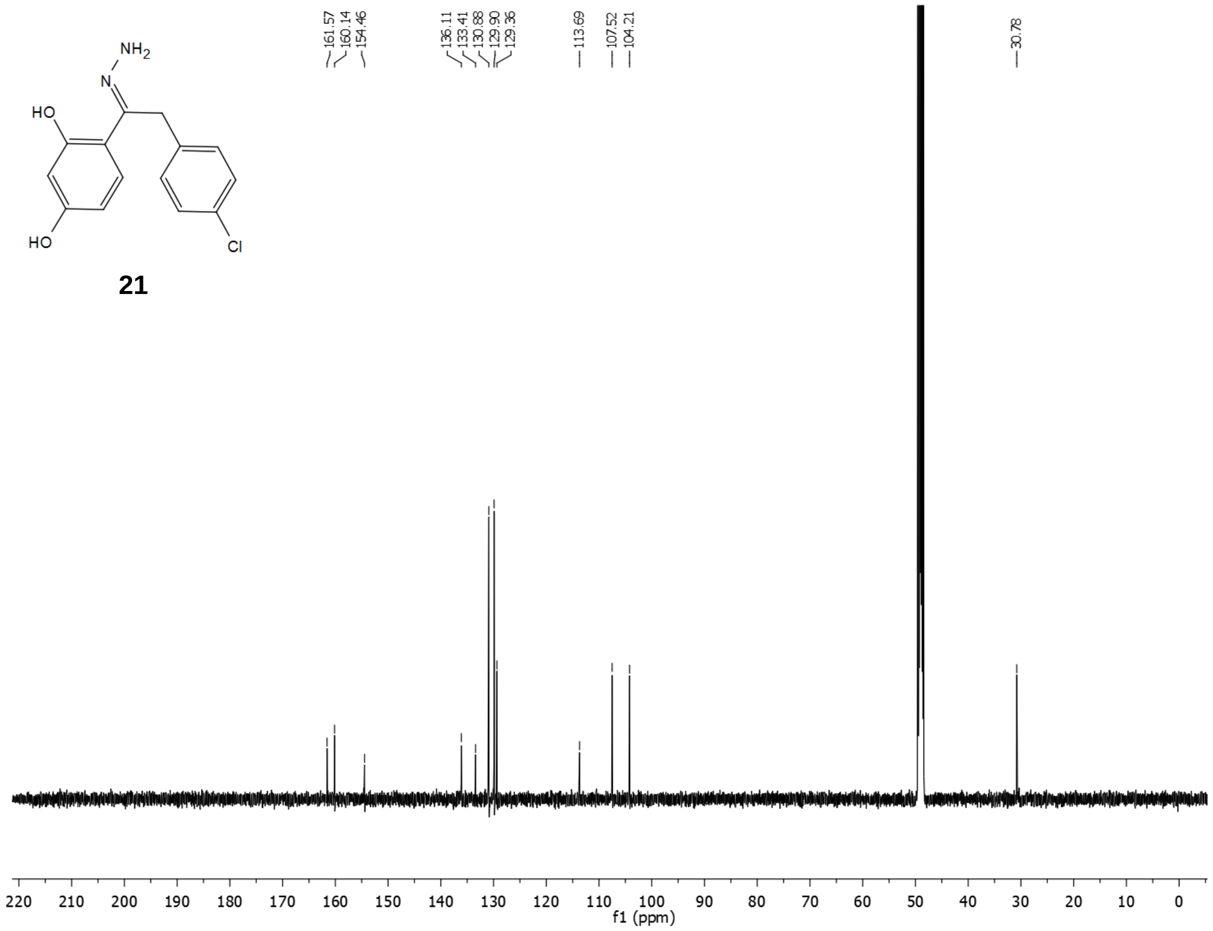

Spectrum 18: ^{13}C NMR of oxime **21**.

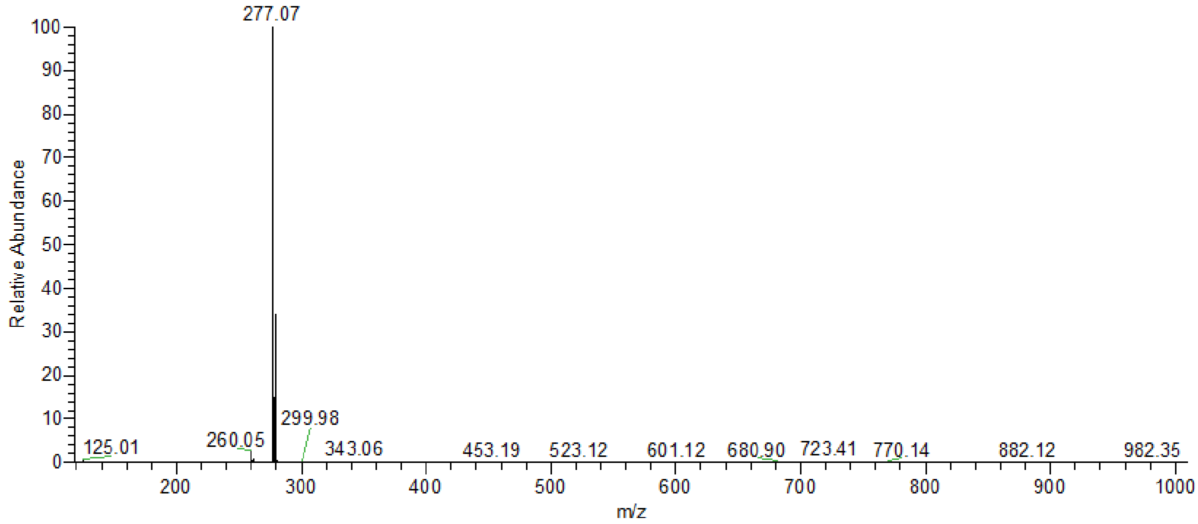

Spectrum 19: HR-ESI-MS spectrum of oxime **21**.

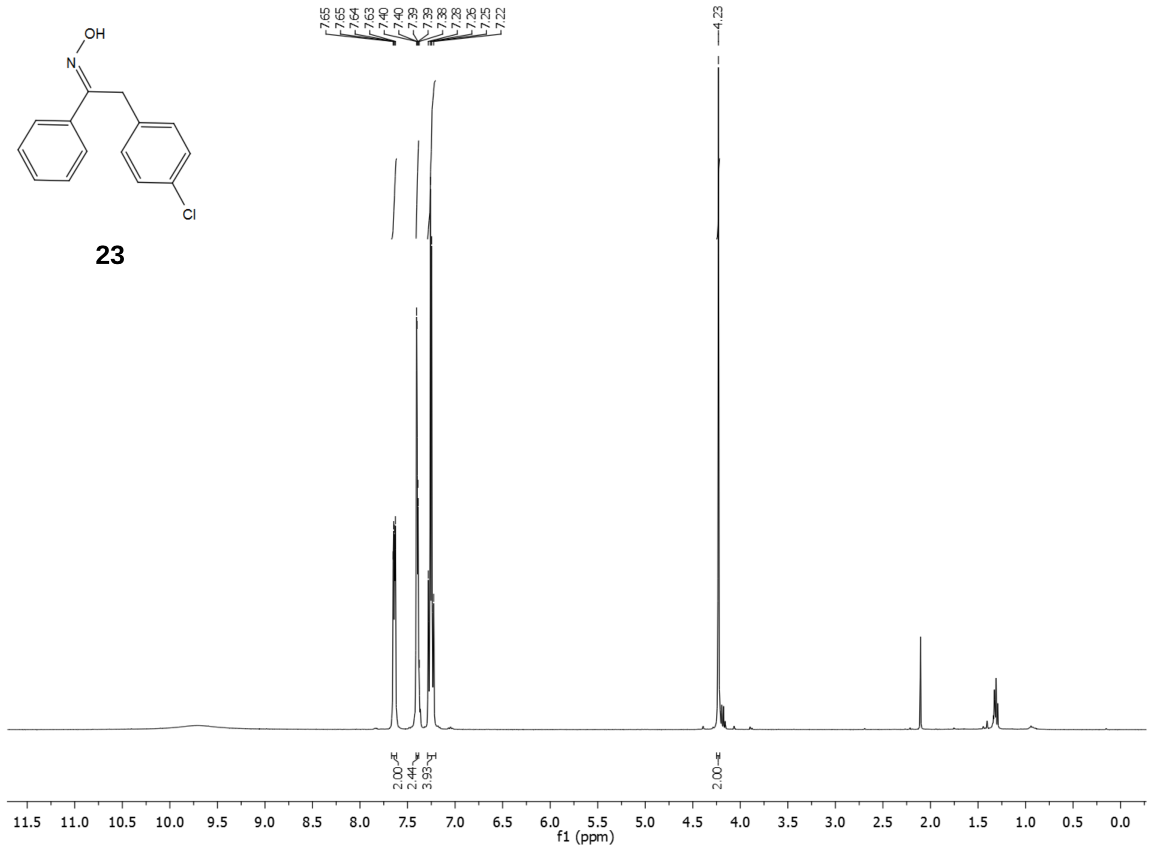

Spectrum 20: ^{1}H NMR of oxime **23**.

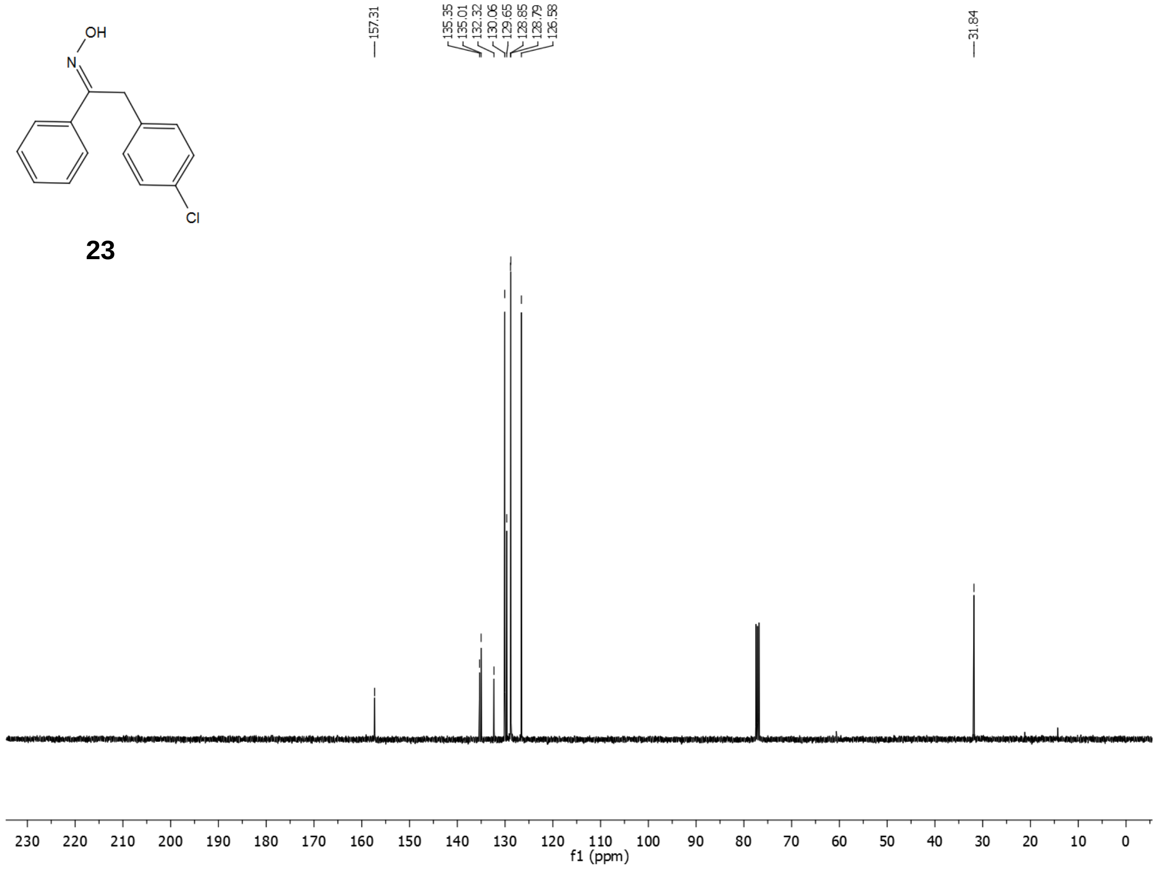

Spectrum 21: ^{13}C NMR of oxime **23**.

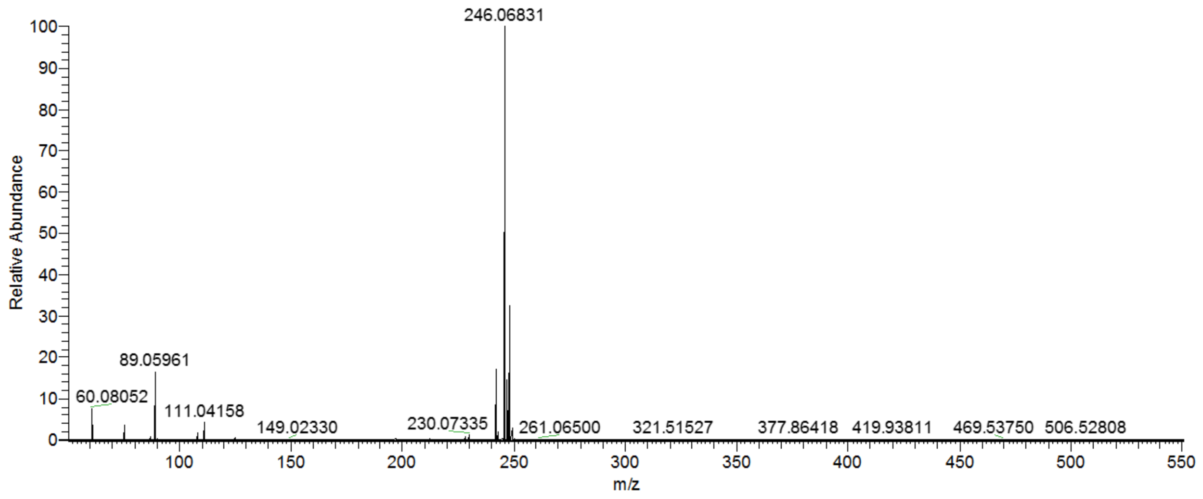

Spectrum 22: HR-ESI-MS spectrum of oxime **23**.

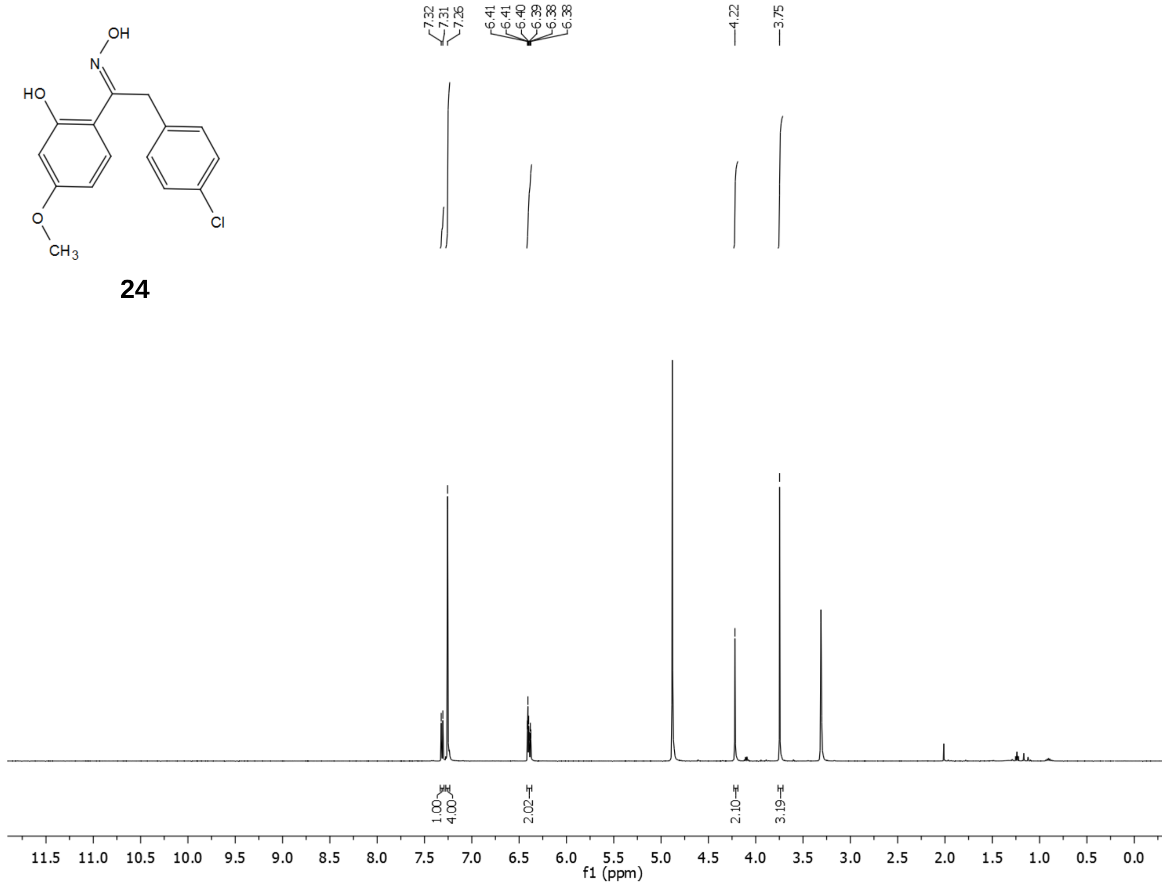

24

Spectrum 23: ¹H NMR of oxime **24**.

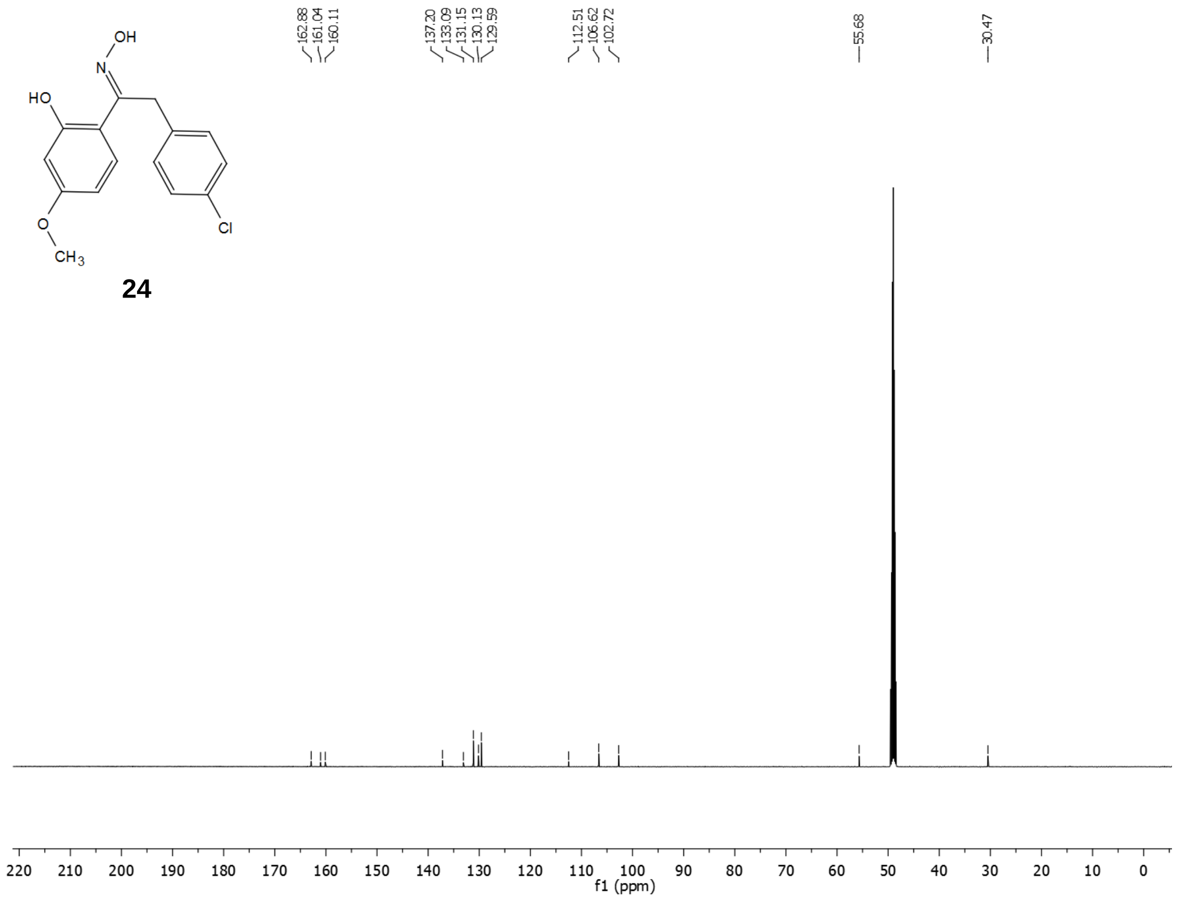

Spectrum 24: ^{13}C NMR of oxime **24**.

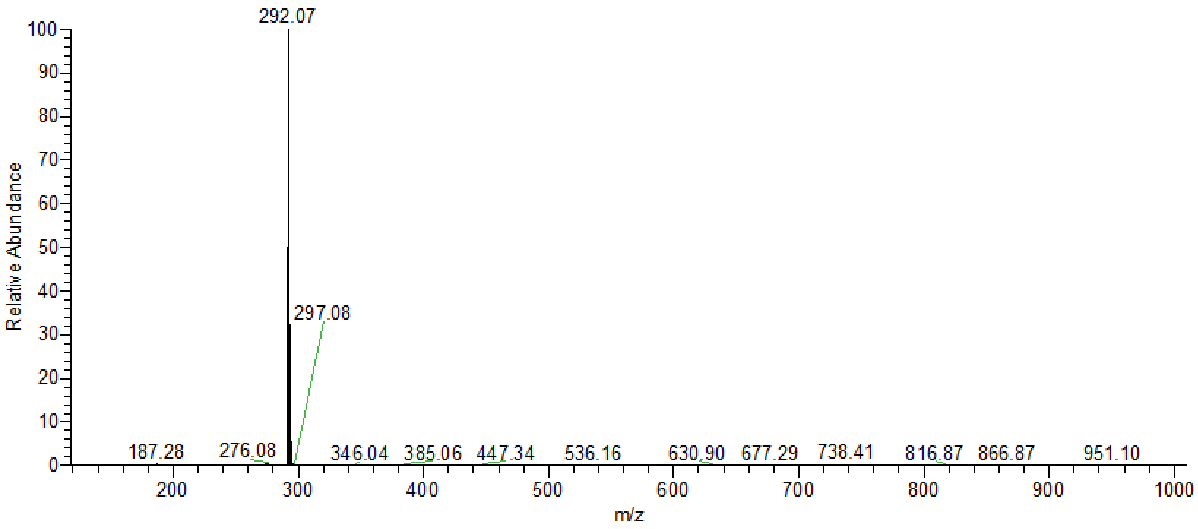

Spectrum 25: HR-ESI-MS spectrum of oxime **24**.

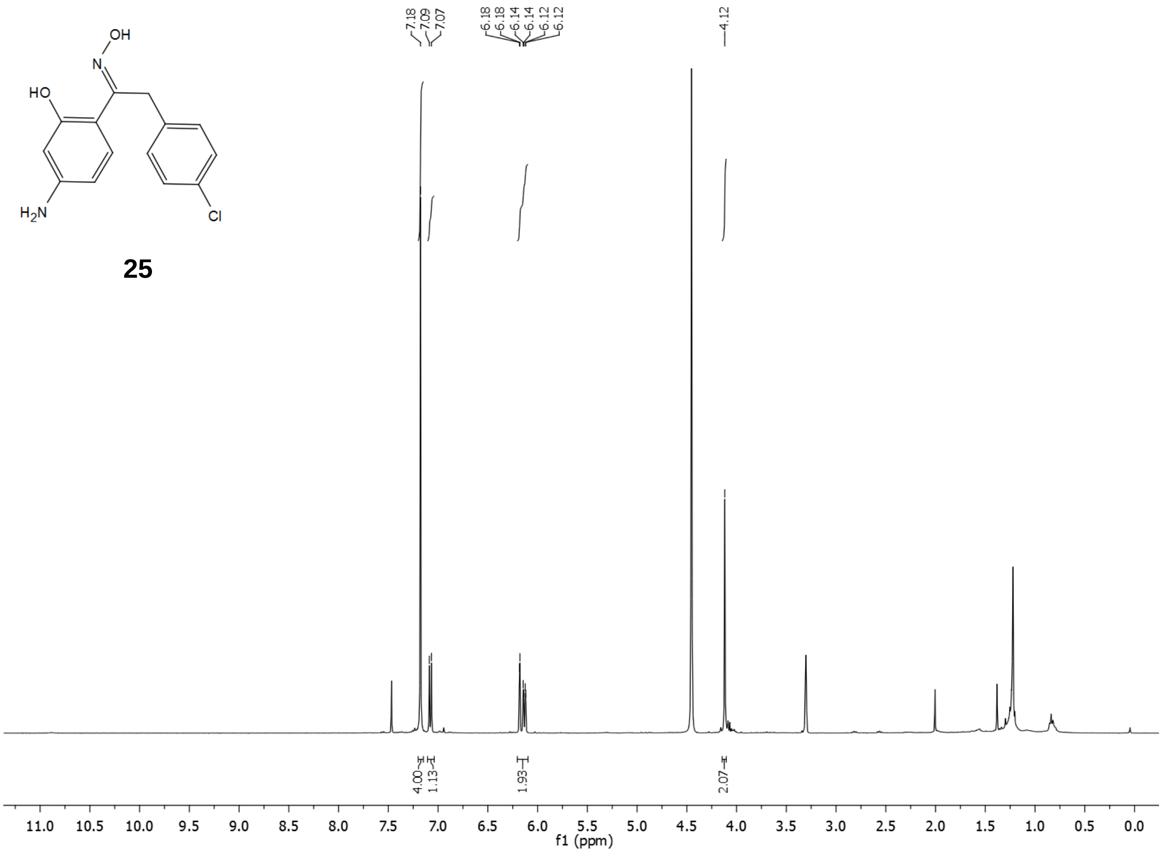

25

Spectrum 26: ^{1}H NMR of oxime **25**.

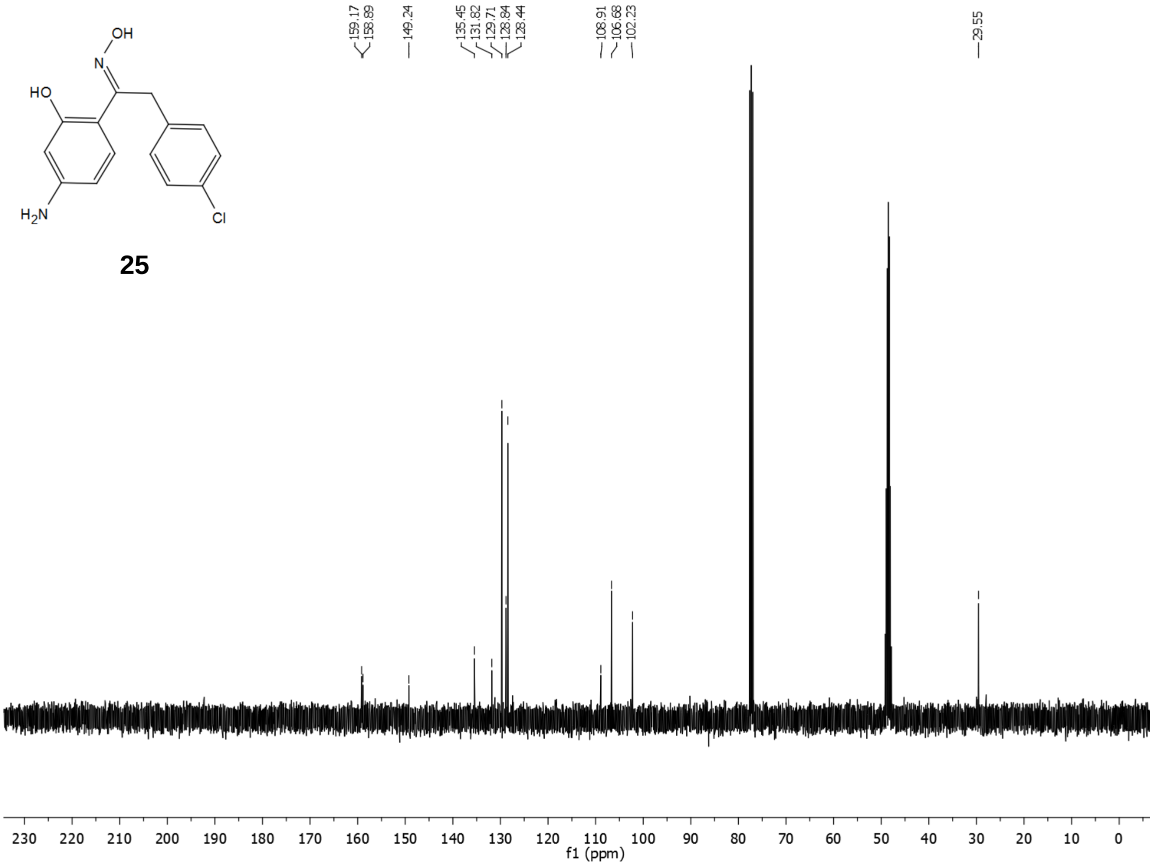

25

Spectrum 27: ^{13}C NMR of oxime **25**.

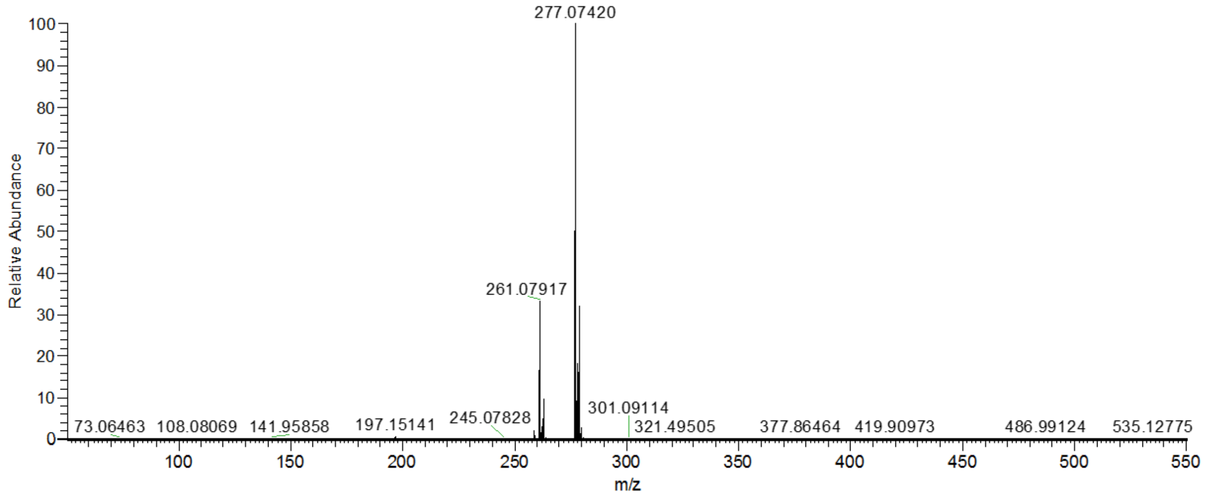

Spectrum 28: HR-ESI-MS spectrum of oxime **25**.

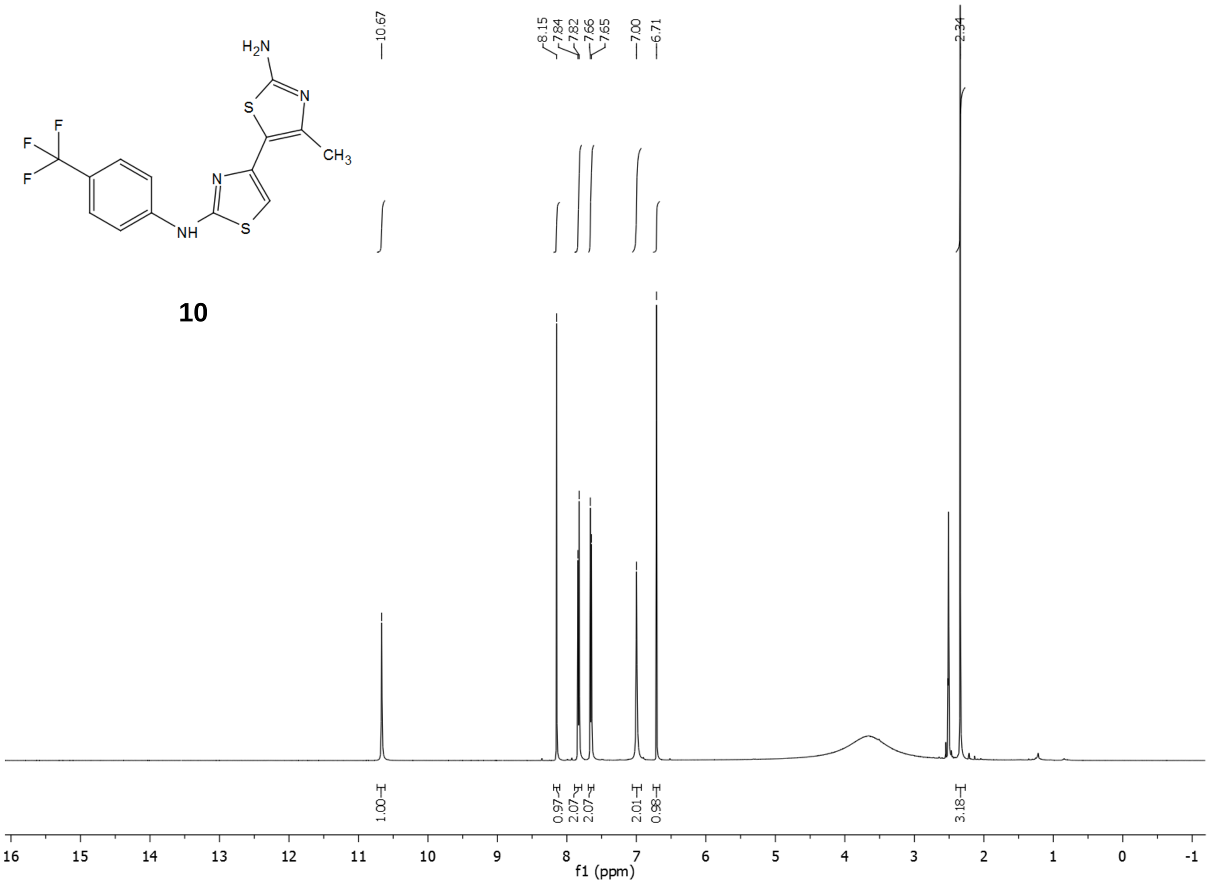

Spectrum 29: ^{1}H NMR of aminothiazole **10**.

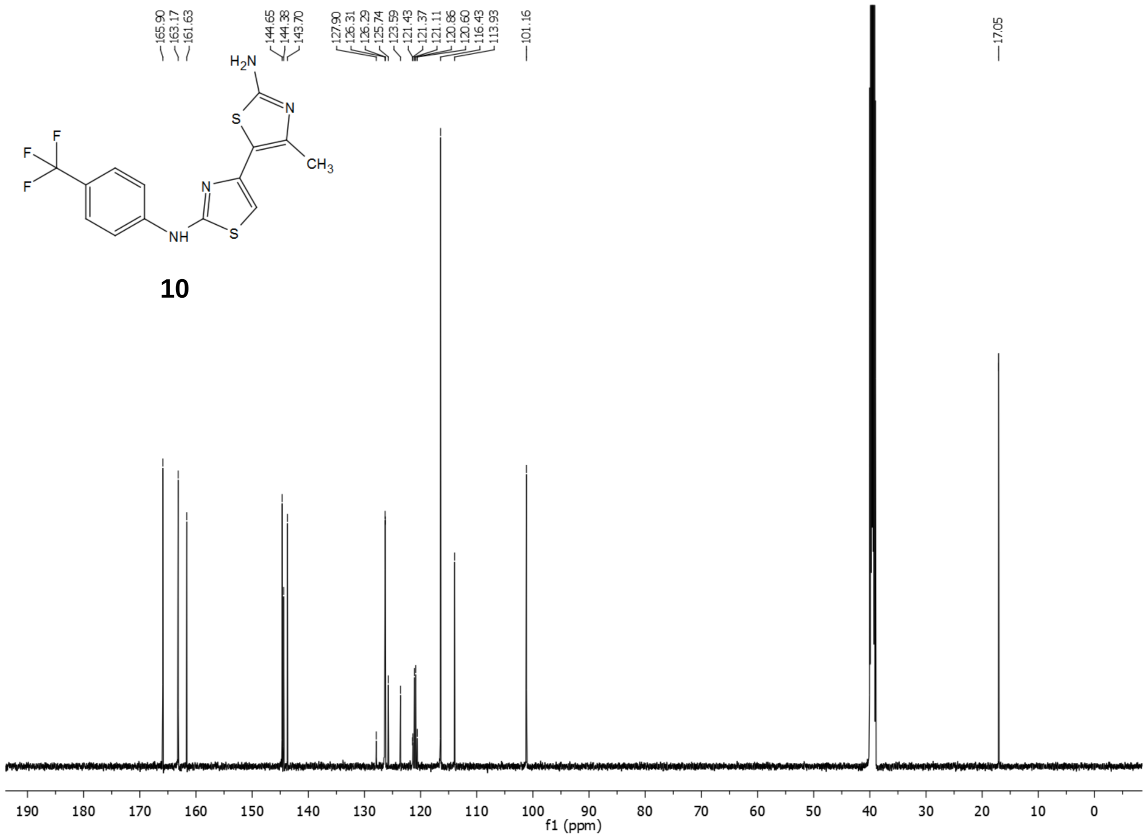

Spectrum 30: ^{13}C NMR of aminothiazole **10**.

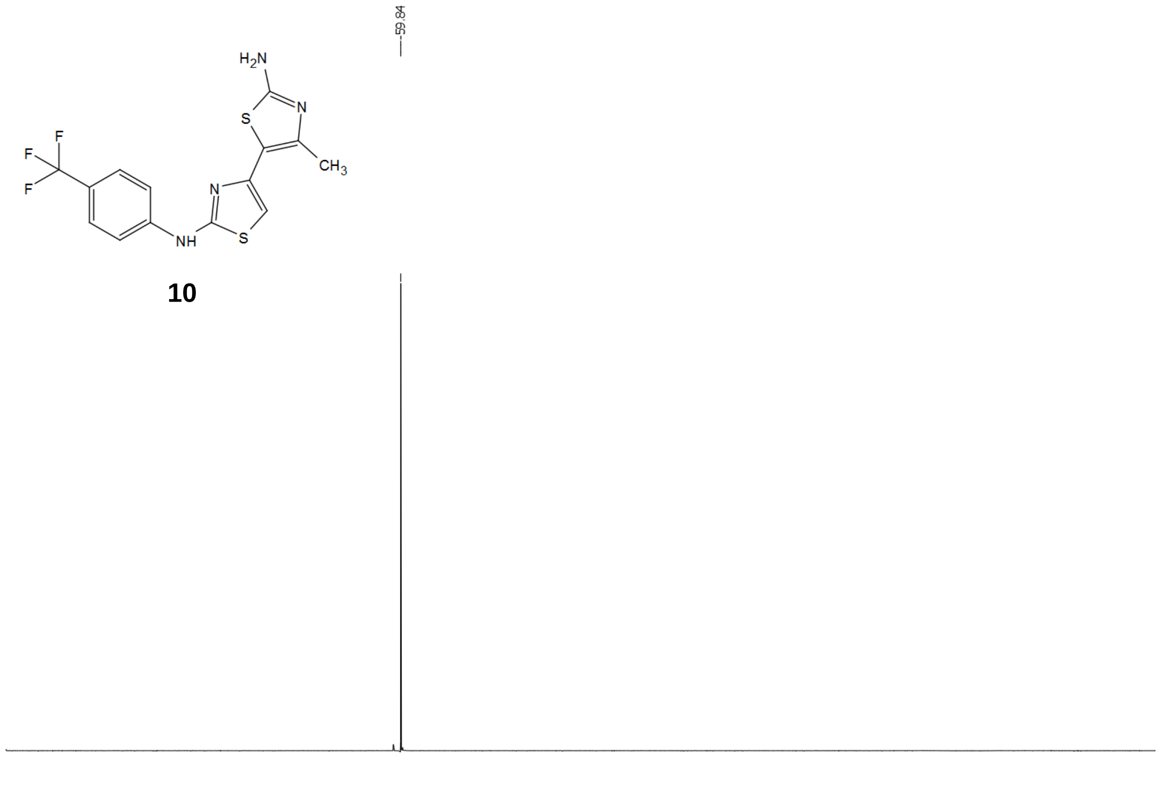

Spectrum 31: ^{19}F NMR of aminothiazole **10**.

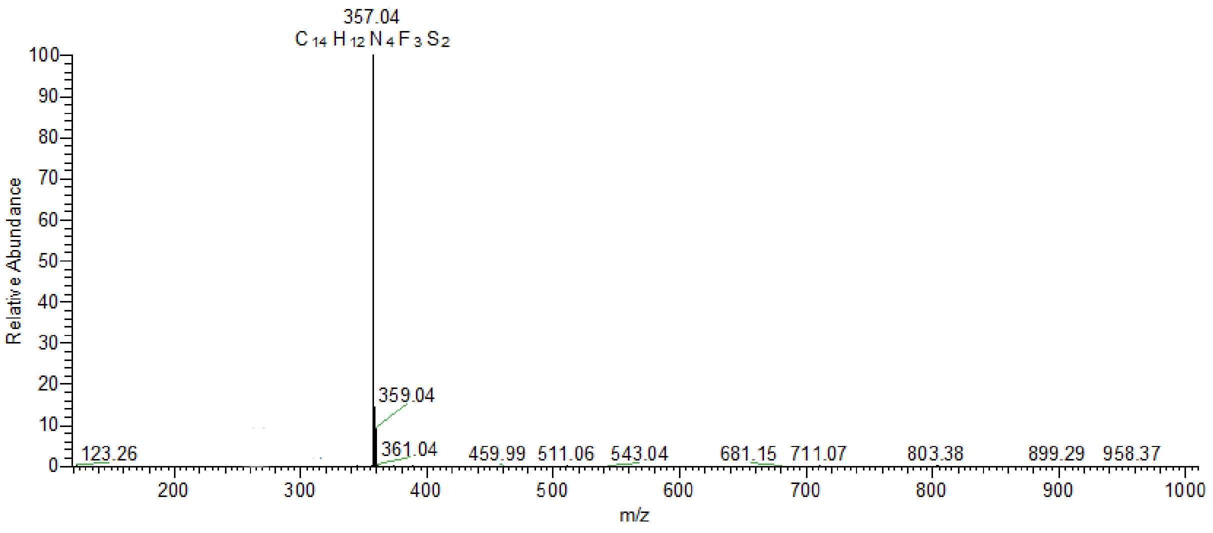

Spectrum 32: HR-ESI-MS spectrum of aminothiazole **10**.

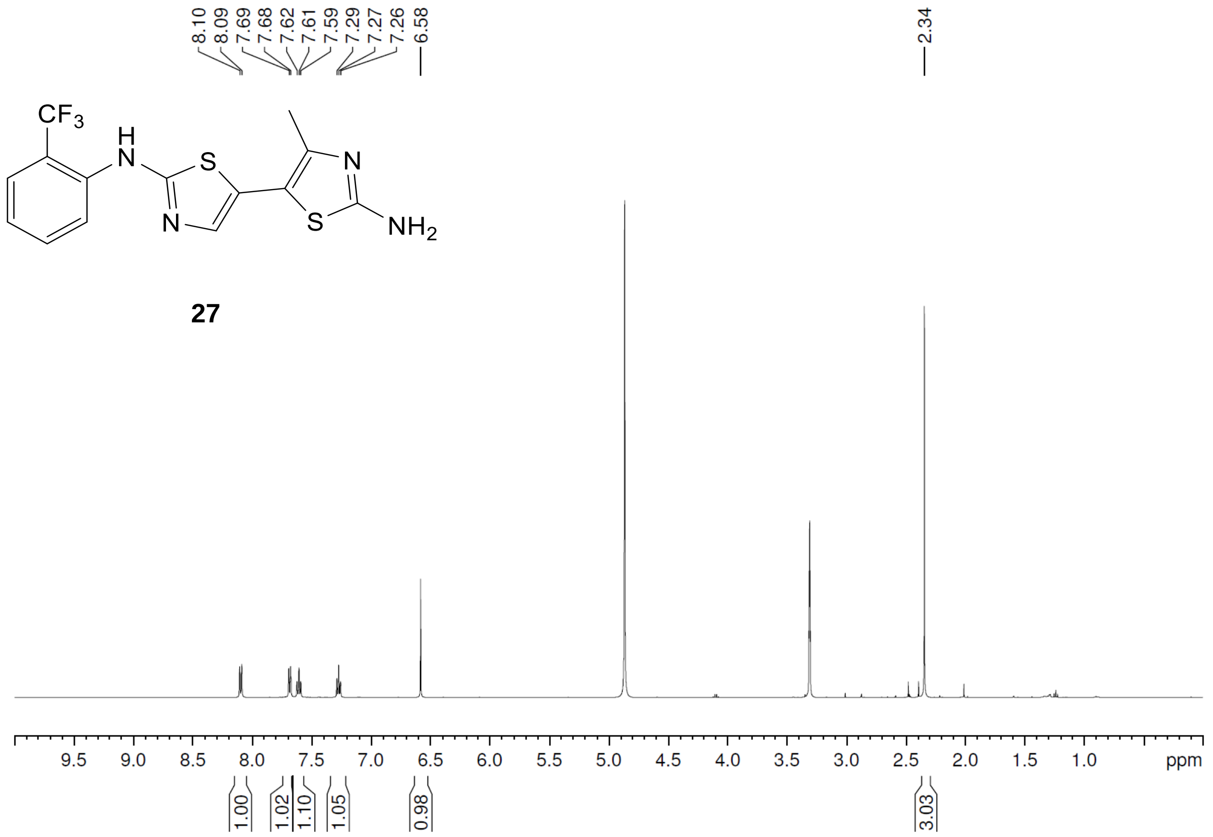

Spectrum 33: ^{1}H NMR of aminothiazole **27**.

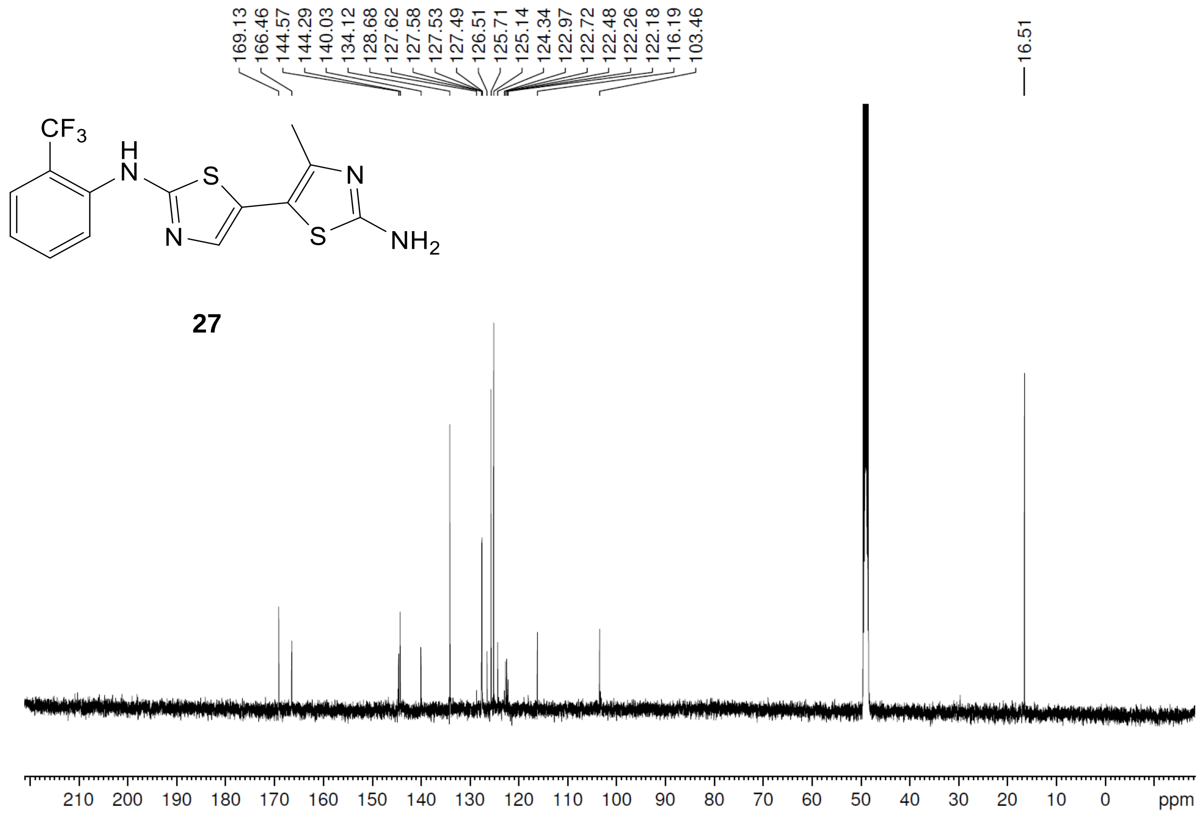

Spectrum 34: ^{13}C NMR of aminothiazole **27**.

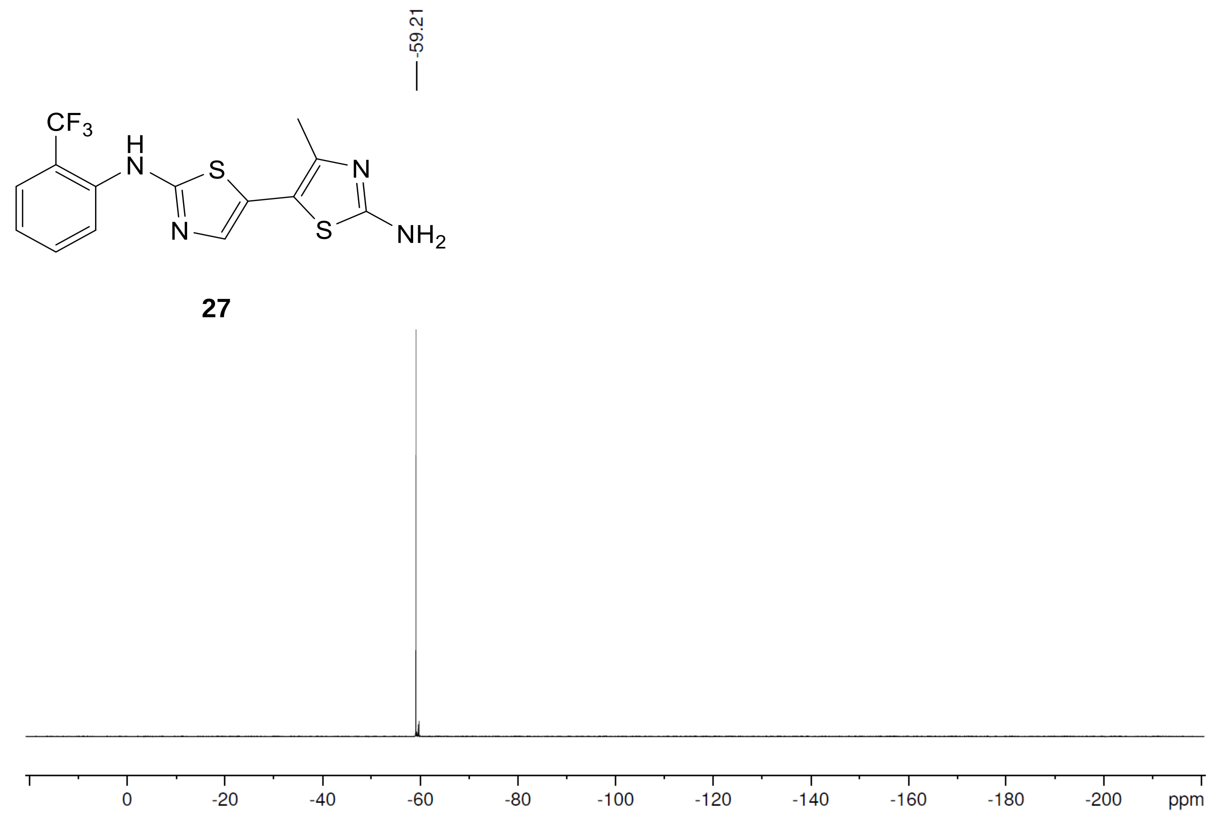

Spectrum 35: ^{19}F NMR of aminothiazole **27**.

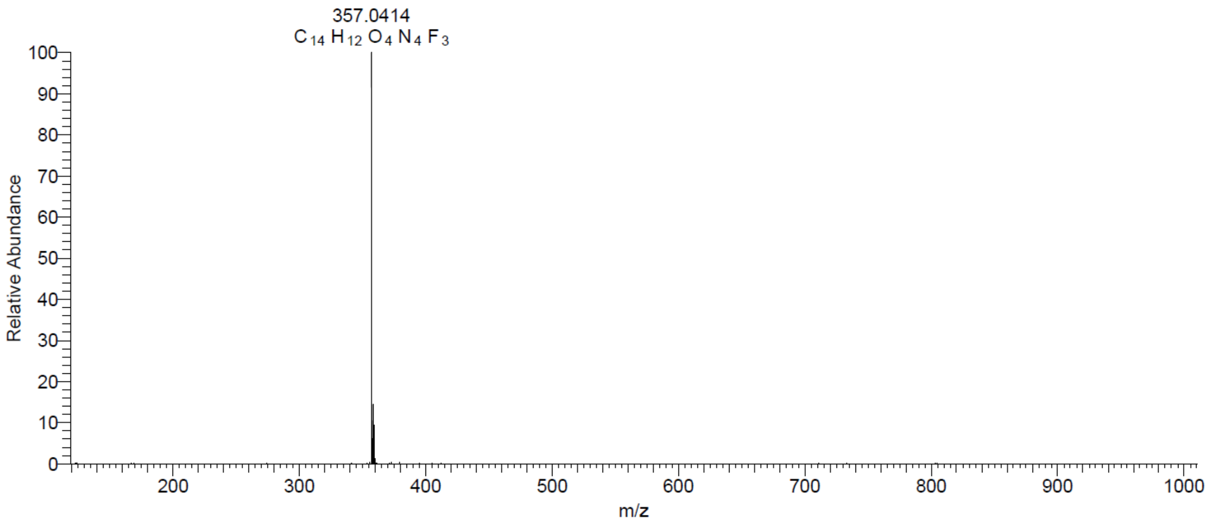

Spectrum 36: HR-ESI-MS spectrum of aminothiazole **27**.

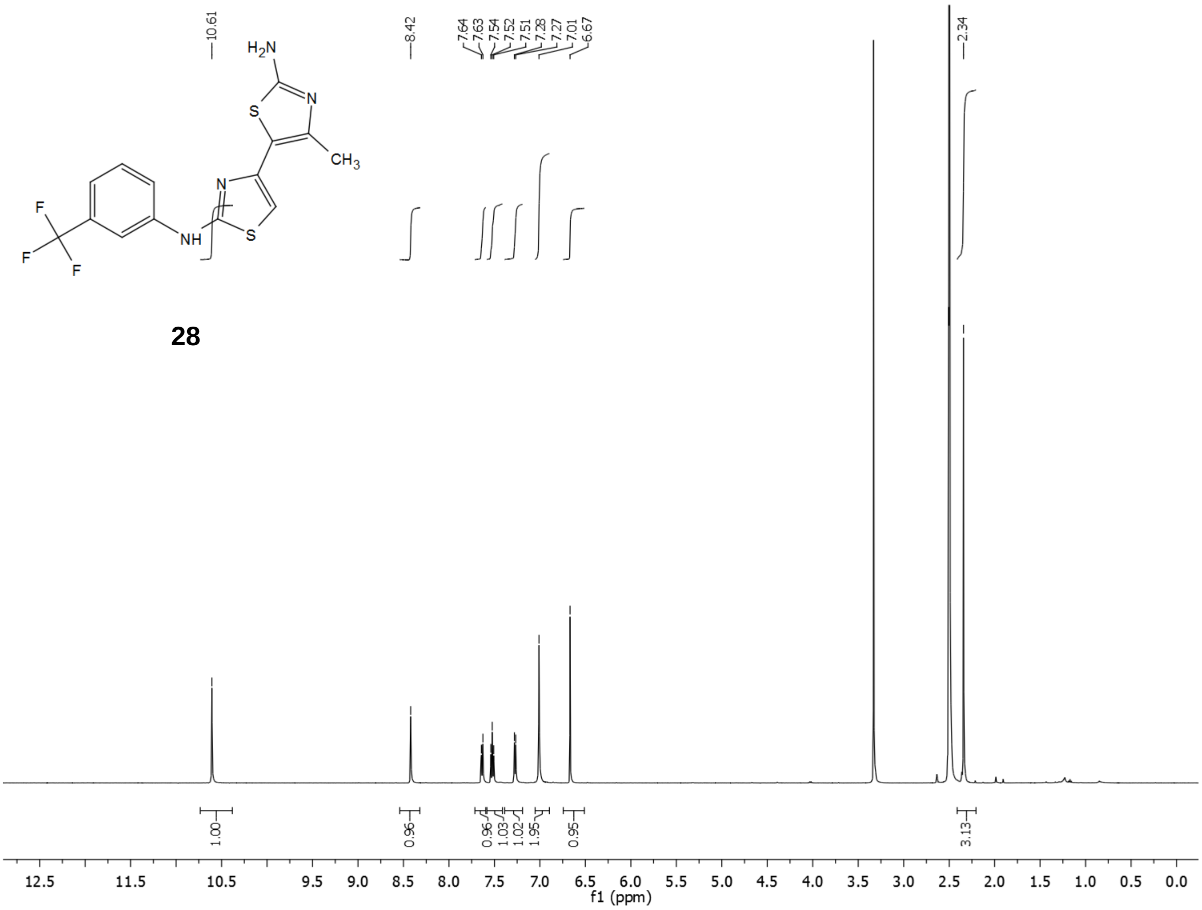

Spectrum 37: ^{1}H NMR of aminothiazole **28**.

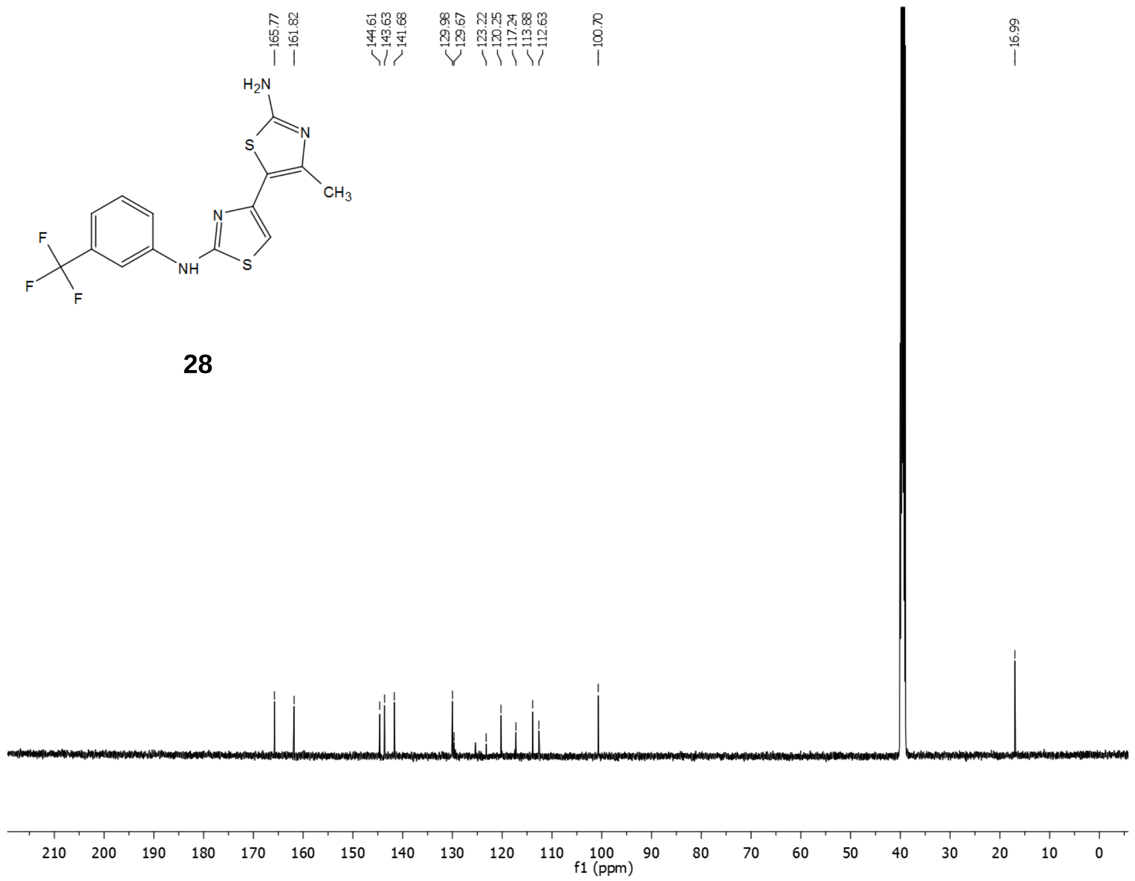

Spectrum 38: ^{13}C NMR of aminothiazole **28**.

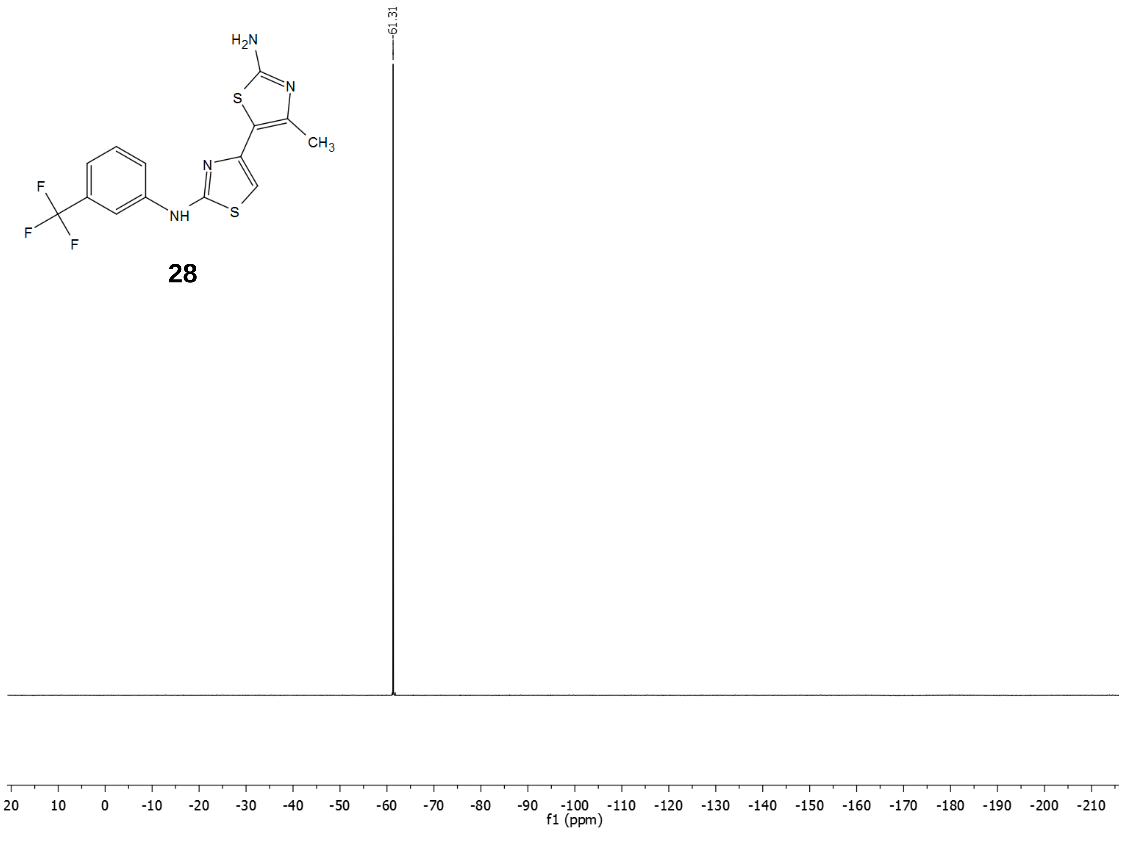

Spectrum 39: ^{19}F NMR of aminothiazole **28**.

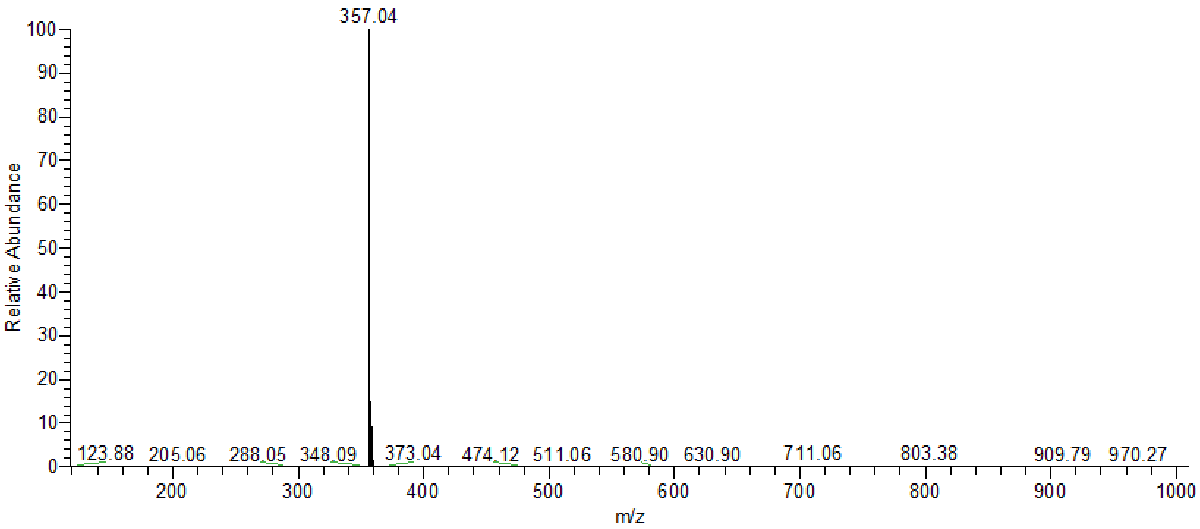

Spectrum 40: HR-ESI-MS spectrum of aminothiazole **28**.

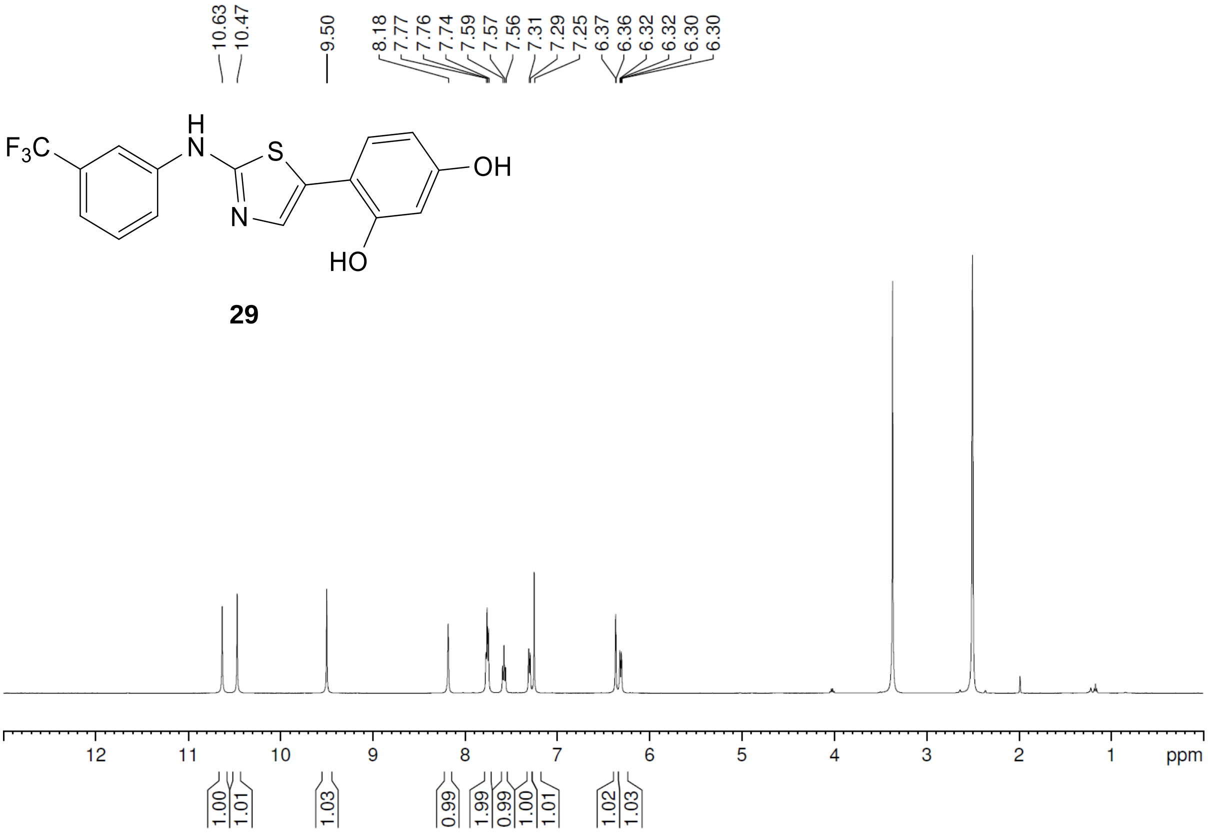

Spectrum 41: ^{1}H NMR of aminothiazole **29**.

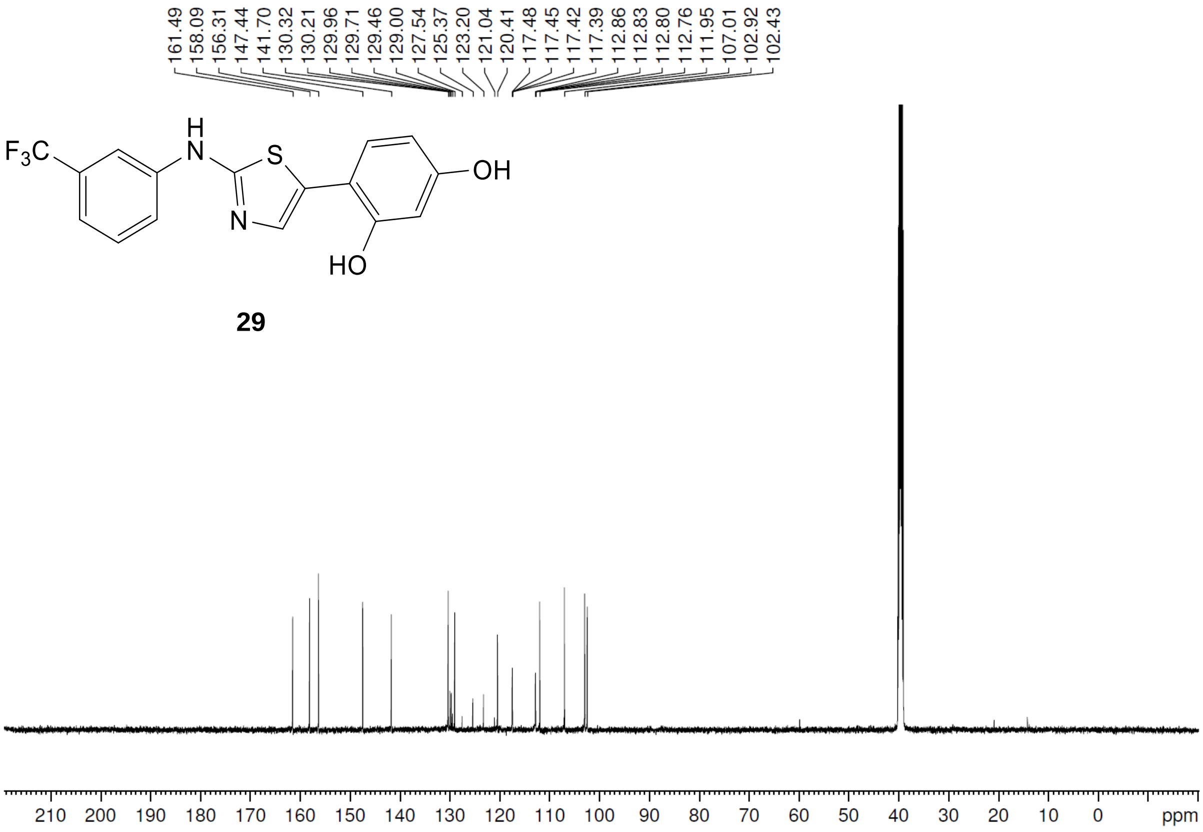

Spectrum 42: ^{13}C NMR of aminothiazole **29**.

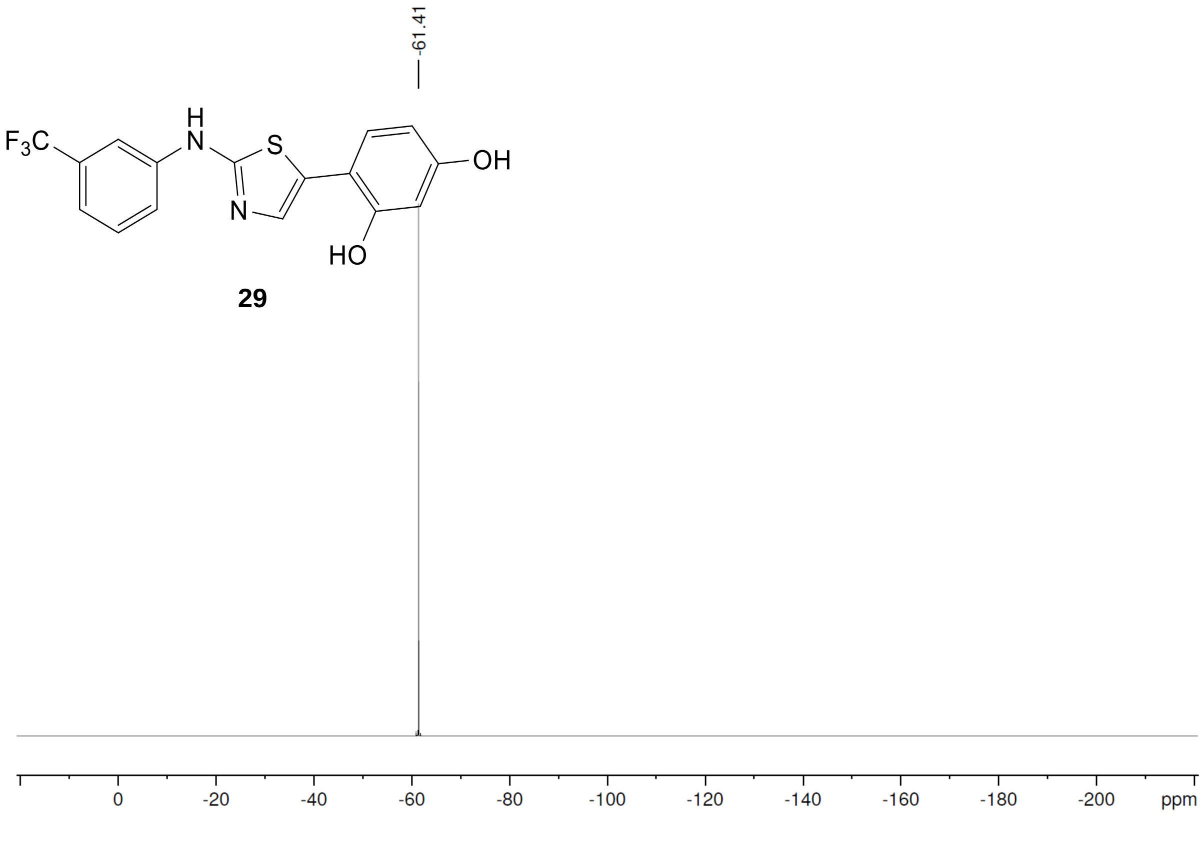

Spectrum 43: ^{19}F NMR of aminothiazole **29**.

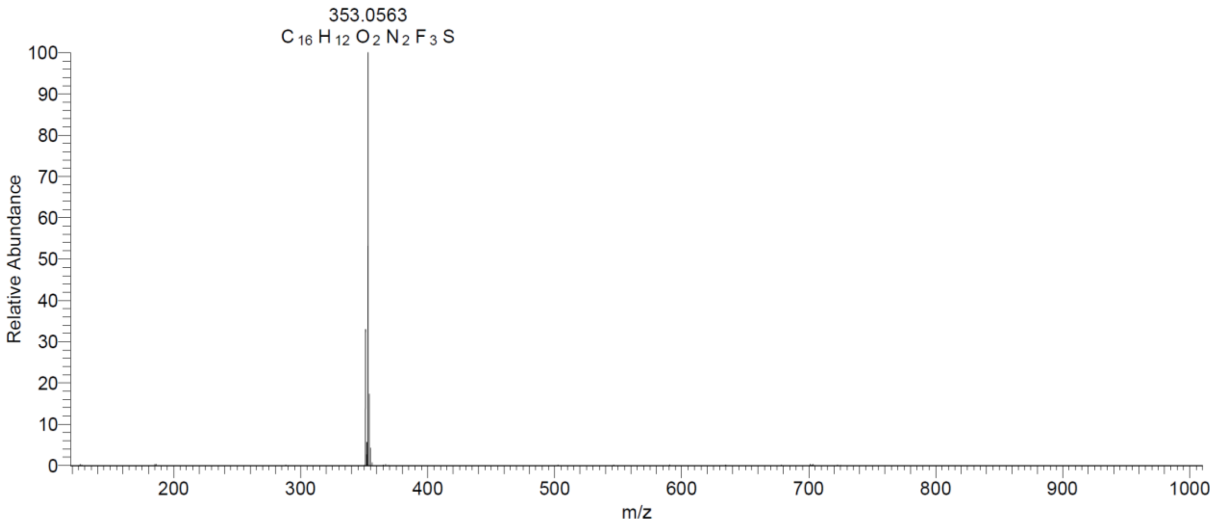

Spectrum 44: HR-ESI-MS spectrum of aminothiazole **29.**

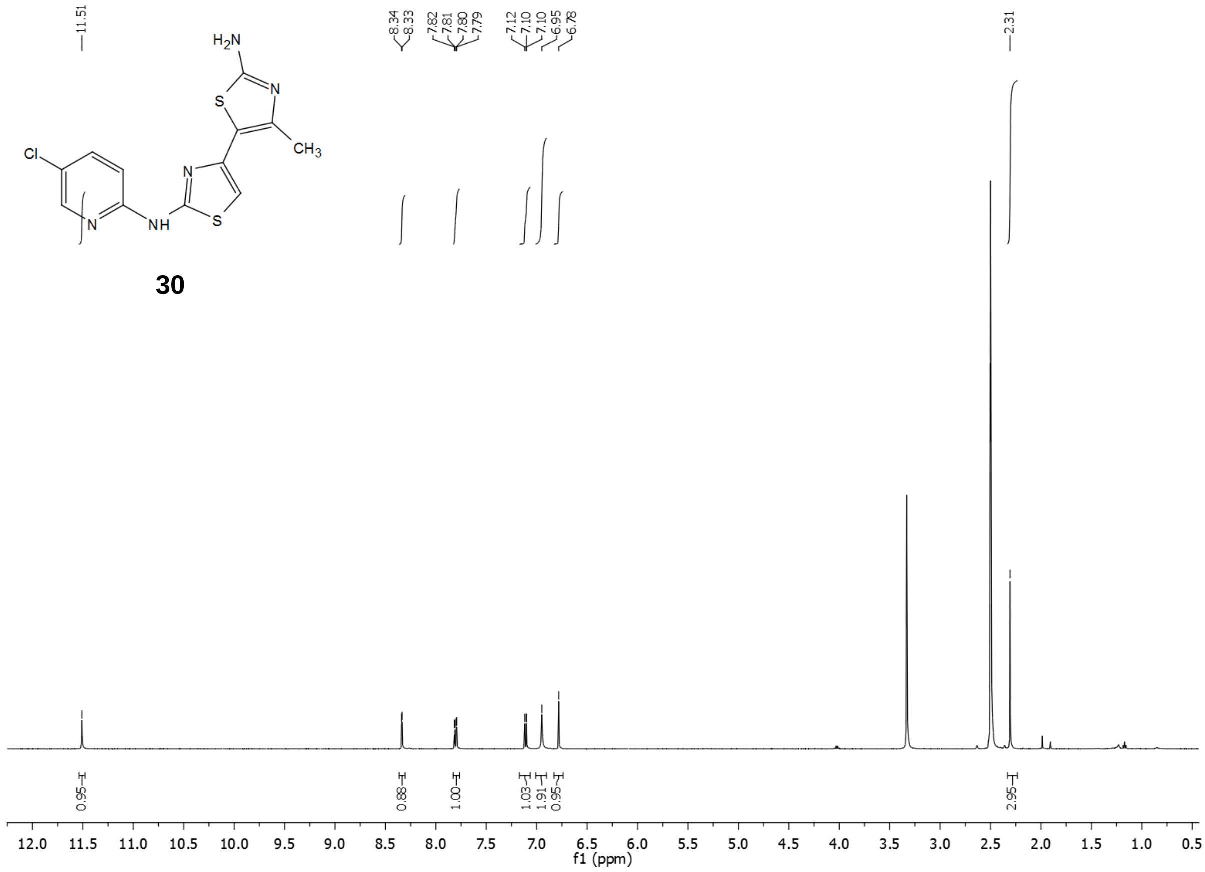

Spectrum 45: ^{1}H NMR of aminothiazole **30**.

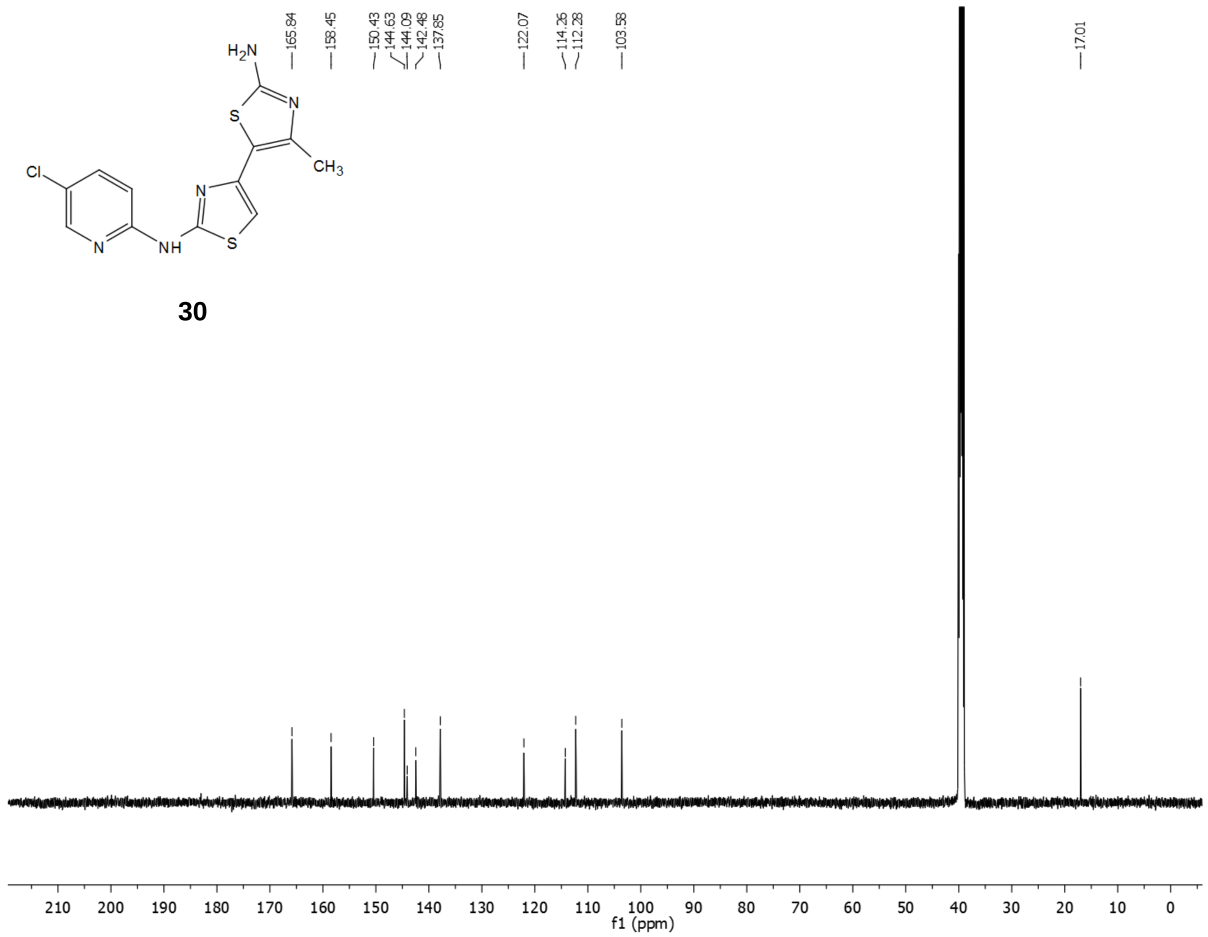

Spectrum 46: ^{13}C NMR of aminothiazole **30**.

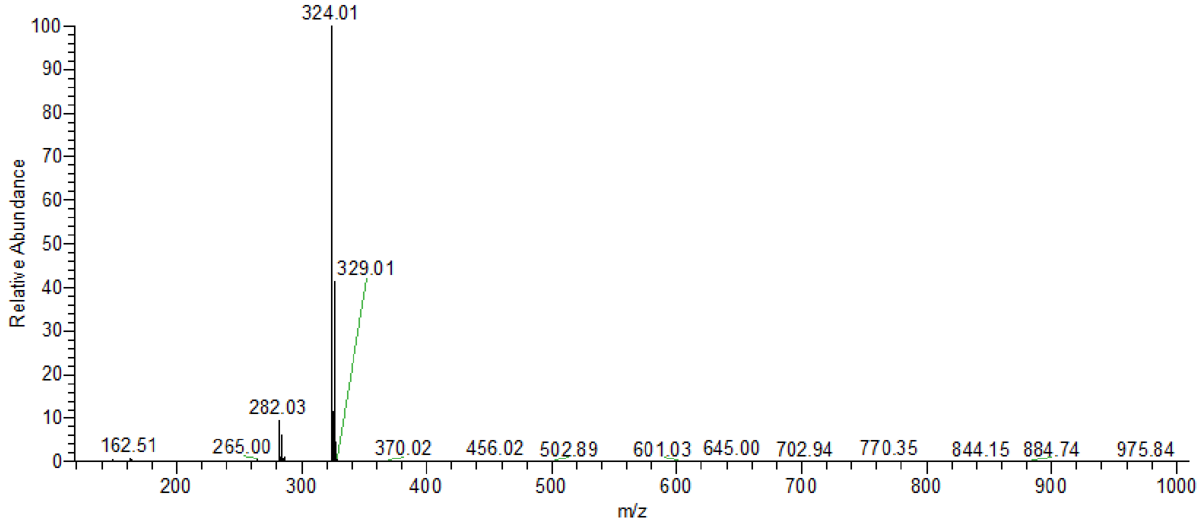

Spectrum 47: HR-ESI-MS spectrum of aminothiazole **30**.
