# Supplementary material for: Discovery of novel drug-like antitubercular hits targeting the MEP pathway enzyme DXPS by strategic application of ligand-based virtual screening
Source: Chem Sci. 2022 Aug 8;13(36):10686–98. doi: 10.1039/d2sc02371g (PMC9491098; doi:10.1039/d2sc02371g)
Supplement: SC-013-D2SC02371G-s004 [file SC-013-D2SC02371G-s004.pdf]

Electronic Supplementary Material (ESI) for Chemical Science.
This journal is © The Royal Society of Chemistry 2022

Supplementary Tables

Table S1: Structures, SMILES codes and supplier ID of hits from the first round of LBVS.

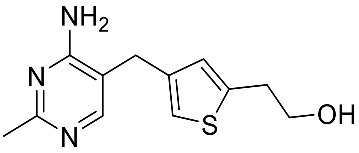

DZT

Cmpd. No.	Structure, SMILES and supplier ID	MW	cLogP	IC_{50} (μM)
**11**	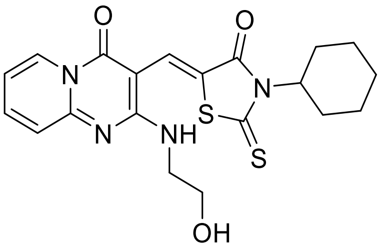<br>OCCNC1=C(\C=C2/SC(=S)N(C3CCCCC 3)C2=O)C(=O)N2C=CC=CC2=N1<br>OSSK_026083	430.54	2.37	60 ± 5
**12**	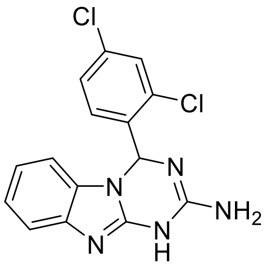<br>NC1=NC(C2=CC=C(C=C2Cl)Cl)N3C(N1) =NC4=CC=CC=C34<br>OSSK_676817	332.19	4.45	145 ± 17
**32**	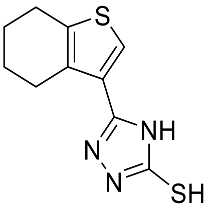<br>SC1=NN=C(C2=CSC3=C2CCCC3)N1<br>OSSK_626533	237.34	3.06	528 ± 118

**33**	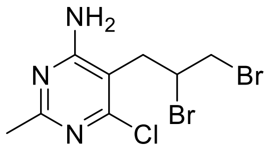 CC1=NC(N)=C(C(Cl)=N1)CC(CBr)Br OSSL_119803	343.45	3.09	748 ± 131
**34**	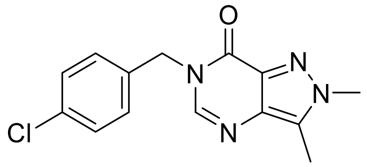 CN1N=C2C(N=CN(C2=O)CC3=CC=C(C=C3)Cl)=C1C OSSL_160939	288.74	2.29	~ 1000
**35**	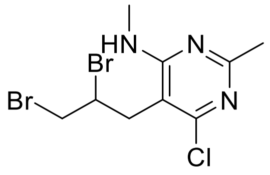 CNC1=C(C(Cl)=NC(C)=N1)CC(CBr)Br OSSK_356137	357.47	3.43	~ 40% inhibition at 1000 μM
**36**	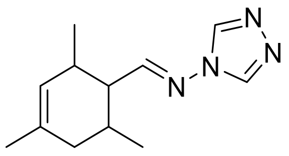 CC1CC(C)=CC(C1/C=N/N2C=NN=C2)C OSSK_356851	218.30	0.87	~ 30 % inhibition at 1000 μM
**37**	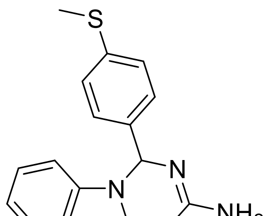 CSC1=CC=C(C2N=C(NC3=NC4=C(N23)C=CC=C4)N)C=C1	309.39	3.87	>60

	OSSK_676806			
**38**	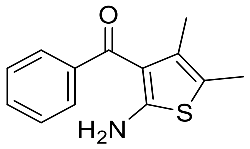<br>CC1=C(C(C(C2=CC=CC=C2)=O)=C(S1)N)C<br><br>OSSK_998770	231.31	4.36	> 120
**39**	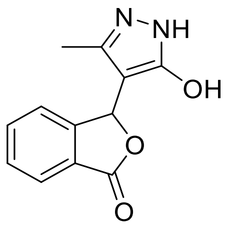<br>CC1=NNC(O)=C1C2OC(C3=CC=CC=C23)=O<br><br>OSSK_734675	230.22	1.53	> 1000
**40**	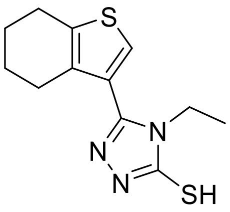<br>CCN1C(S)=NN=C1C2=CSC3=C2CCCC3<br><br>OSSK_040776	265.39	3.64	> 240
**41**	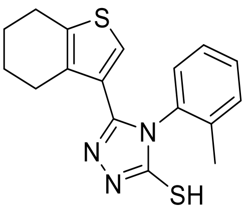<br>CC1=CC=CC=C1N2C(S)=NN=C2C3=CSC4=C3CCCC4<br><br>OSSK_041451	327.46	5.46	> 8

**42**	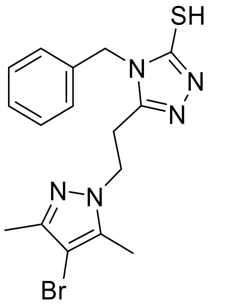<br>CC1=NN(C(C)=C1Br)CCC2=NN=C(N2CC3=CC=CC=C3)S<br>OSSK_041558	392.32	3.18	> 30
**43**	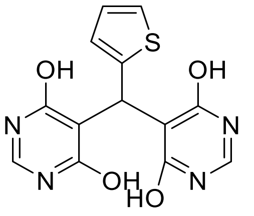<br>OC1=NC=NC(O)=C1C(C2=C(N=CN=C2O)O)C3=CC=CS3<br>OSSK_373400	318.31	2.72	> 500
**44**	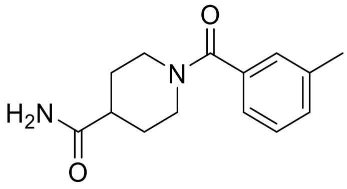<br>CC1=CC=CC(C(N2CCC(C(N)=O)CC2)=O)=C1<br>OSSK_389140	246.31	1.05	> 1000
**45**	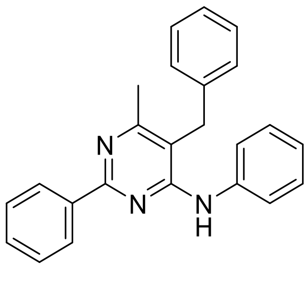<br>CC1=C(C(NC2=CC=CC=C2)=NC(C3=CC=CC=C3)=N1)CC4=CC=CC=C4<br>OSSK_468268	351.45	6.60	> 15

**46**	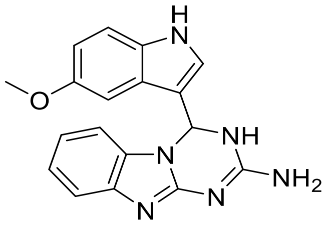<br>COC1=CC=C2NC=C(C2=C1)C3NC(N)=N<br>C4=NC5=C(N34)C=CC=C5<br><br>OSSK_531783	332.37	2.74	> 500
**47**	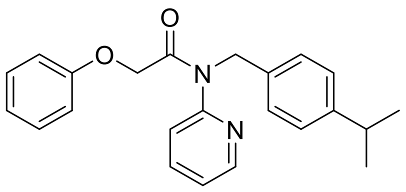<br>CC(C1=CC=C(C=C1)CN(C2=CC=CC=N2<br>)C(COC3=CC=CC=C3)=O)C<br><br>OSSK_771297	360.46	4.94	>30
**48**	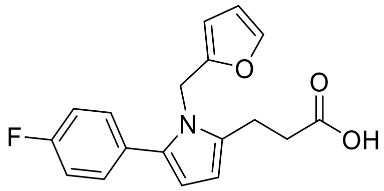<br>OC(CCC1=CC=C(C2=CC=C(C=C2)F)N1<br>CC3=CC=CO3)=O<br><br>OSSK_995312	313.33	3.62	> 500
**49**	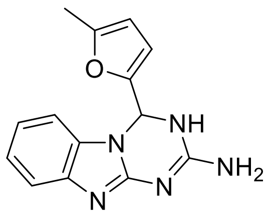<br>CC1=CC=C(C2NC(N)=NC3=NC4=C(N23)<br>C=CC=C4)O1<br><br>OSSL_009157	267.29	2.06	> 1000

**50**	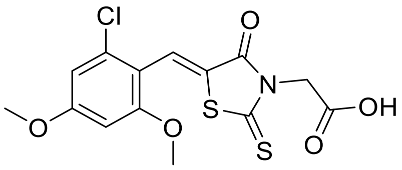 COC1=CC(OC)=C(C(Cl)=C1)/C=C2SC(N(C\2=O)CC(O)=O)=S OSSL_122234	373.82	2.73	> 240
**51**	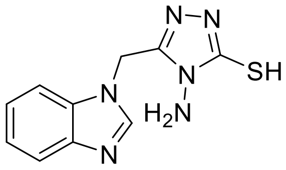 NN1C(S)=NN=C1CN2C=NC3=CC=CC=C23 OSSL_259003	246.29	1.22	> 1000
**52**	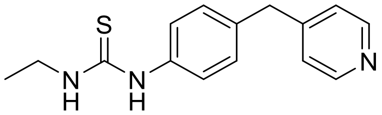 CCNC(NC1=CC=C(C=C1)CC2=CC=NC=C2)=S OSSL_301953	271.38	3.22	> 240
	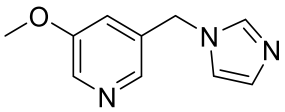 **1**			
**Cmpd No.**	**Structure, SMILES and supplier ID**	**MW**	**cLogP**	**IC_{50} (μM)**
**3**	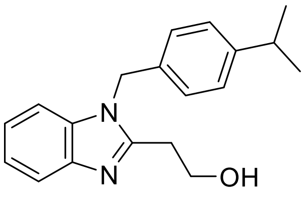 CC(C1=CC=C(C=C1)CN2C(CCO)=NC3=C2C=CC=C3)C OSSK_927261	294.40	4.00	317 ± 29

**4**	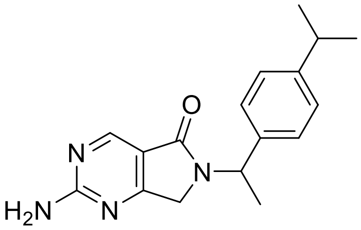 CC(C1=CC=C(C(N2CC3=C(C2=O)C=NC(N)=N3)C)C=C1)C OSSL_332078	296.37	2.42	935 ± 117
**53**	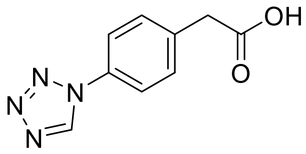 OC(CC1=CC=C(N2C=NN=N2)C=C1)=O OSSL_014662	204.19	0.65	~ 25 % inhibition at 1000 μM
**54**	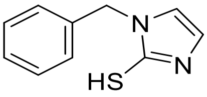 SC1=NC=CN1CC2=CC=CC=C2 OSSL_312724	190.26	2.47	> 500
**55**	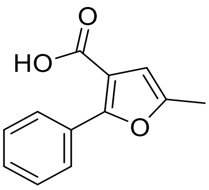 CC1=CC(C(O)=O)=C(C2=CC=CC=C2)O1 OSSL_295114	202.21	2.54	> 1000
**56**	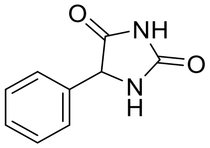 O=C1NC(C2=CC=CC=C2)C(N1)=O OSSL_299665	176.18	0.49	> 1000

**57**	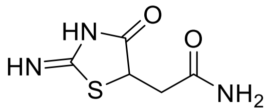<br>NC(CC1SC(NC1=O)=N)=O<br>OSSK_303386	173.19	-1.15	> 1000
**58**	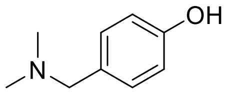<br>CN(CC1=CC=C(C=C1)O)C<br>OSSK_366315	151.21	1.61	> 1000
**59**	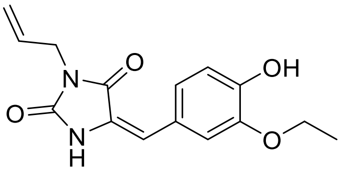<br>CCOC1=CC(/C=C2NC(N(C/2=O)CC=C)=O)=CC=C1O<br>OSSK_441886	288.30	1.53	> 30
**60**	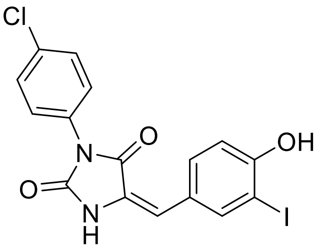<br>OC1=CC=C(C=C1I)/C=C2NC(N(C3=CC=C(C=C3)Cl)C/2=O)=O<br>OSSK_441901	440.62	3.79	> 120
**61**	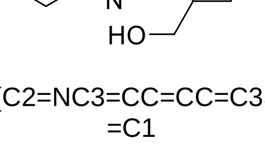<br>OCC1=C(C2=NC3=CC=CC=C3S2)N=CC=C1<br>OSSK_470489	242.30	2.54	> 60

**62**	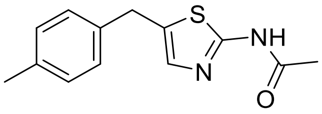<br>CC(NC1=NC=C(S1)CC2=CC=C(C=C2)C)=O<br>OSSK_475789	246.33	3.29	> 30
**63**	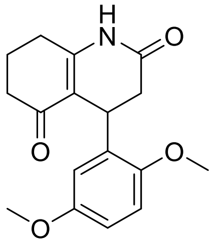<br>COC1=CC=C(C(C2CC(NC3=C2C(CCC3)=O)=O)=C1)OC<br>OSSK_614781	301.34	1.13	> 500
**64**	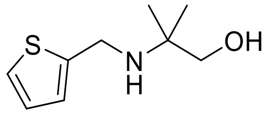<br>CC(CO)(NCC1=CC=CS1)C<br>OSSK_616553	185.29	1.45	> 1000
**65**	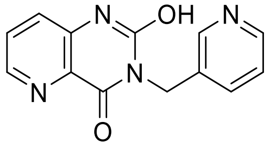<br>OC1=NC2=C(C(N1CC3=CN=CC=C3)=O)N=CC=C2<br>OSSK_840158	254.25	1.30	>240
**66**	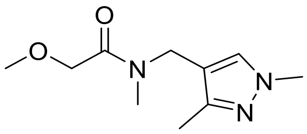<br>COCC(N(CC1=CN(N=C1C)C)C)=O<br>OSSK_891366	211.27	-0.47	>1000

**67**	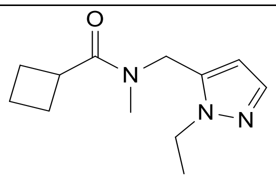<br>CCN1N=CC=C1CN(C(C2CCC2)=O)C<br>OSSK_891525	221.30	1.07	>1000
**68**	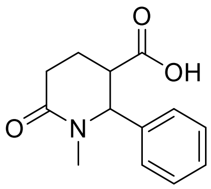<br>CN1C(C2=CC=CC=C2)C(C(O)=O)CCC1=O<br>OSSL_107834	233.27	1.25	> 1000
**69**	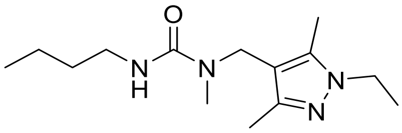<br>CCCCNC(N(CC1=C(N(N=C1C)CC)C)C)=O<br>OSSL_161624	266.39	1.47	> 1000
**70**	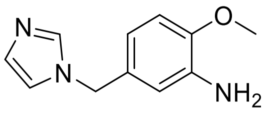<br>COC1=CC=C(C=C1N)CN2C=CN=C2<br>OSSL_303243	203.25	0.82	> 1000
**71**	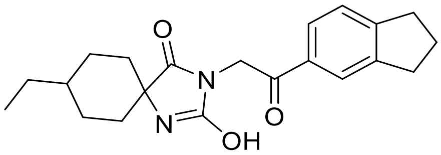<br>CCC1CCC2(N=C(N(C2=O)CC(C3=CC=C4CCCC4=C3)=O)O)CC1<br>OSSL_309452	354.45	3.77	> 60

**72**	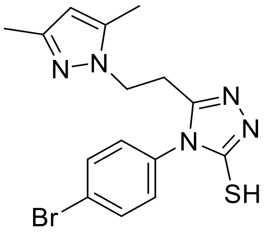<br>CC1=CC(C)=NN1CCC2=NN=C(N2C3=CC=C(C=C3)Br)S<br>OSSL_311660	378.29	3.65	> 120
**73**	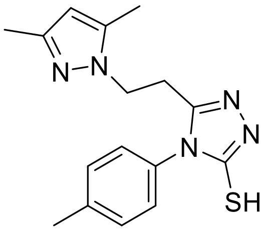<br>CC1=CC(C)=NN1CCC2=NN=C(N2C3=CC=C(C=C3)C)S<br>OSSL_311662	313.42	3.40	> 500
**74**	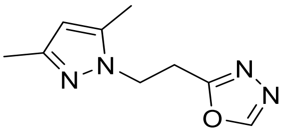<br>CC1=CC(C)=NN1CCC2=NN=CO2<br>OSSL_311751	192.22	-0.25	> 1000
**75**	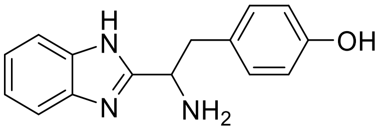<br>NC(C1=NC2=CC=CC=C2N1)CC3=CC=C(C=C3)O<br>OSSL_317110	253.31	2.38	> 1000
	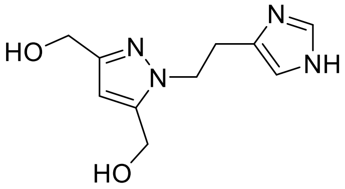<br>**2**			

Cmpd. No.	Structure, SMILES and supplier ID	MW	cLogP	IC$_{50}$ (μM)
**5**	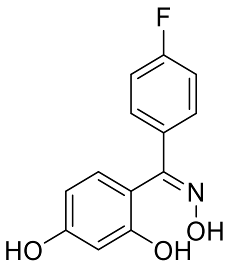<br>O/N=C(C1=CC=C(C=C1O)O)/C2=CC=C(C=C2)F<br>OSSL_151911	247.23	2.98	528 ± 43
**6**	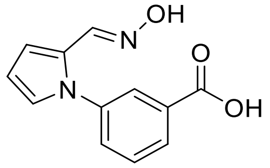<br>O/N=C/C1=CC=CN1C2=CC=CC(C(O)=O)=C2<br>OSSK_600760	230.22	2.23	455 ± 81
**76**	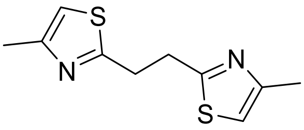<br>CC1=CSC(CCC2=NC(C)=CS2)=N1<br>OSSK_269157	224.34	1.82	~ 25 % inhibition at 1000 μM
**77**	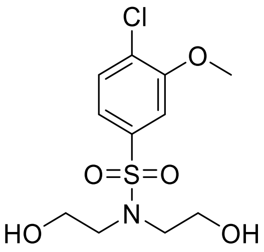<br>COC1=CC(S(=O)(N(CCO)CCO)=O)=CC=C1Cl<br>OSSL_292991	309.76	0.09	~ 25 % inhibition at 2000 μM
**78**	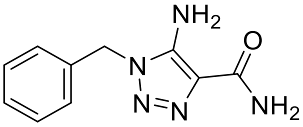	217.23	0.89	> 240

	NC(C1=C(N(N=N1)CC2=CC=CC=C2)N)=<br>O<br><br>OSSL_296278			
**79**	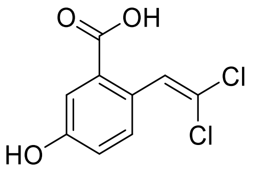<br>OC(C1=C(C=CC(O)=C1)/C=C(Cl)\Cl)=O<br><br>OSSL_304940	233.04	2.82	> 1000
**80**	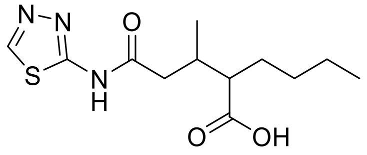<br>CCCCC(C(O)=O)C(CC(NC1=NN=CS1)=<br>O)C<br><br>OSSK_308957	285.36	2.10	> 1000
**81**	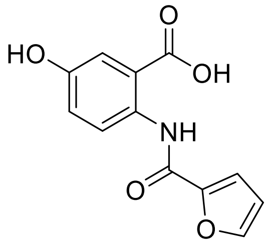<br>OC(C1=CC(O)=CC=C1NC(C2=CC=CO2)<br>=O)=O<br><br>OSSK_403603	247.21	2.13	>60
**82**	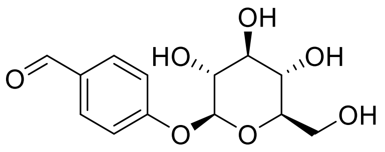<br>OC[C@H]1O[C@H]([C@@H]([C@H]([C<br>@@H]1O)O)O)OC2=CC=C(C=C2)C=O<br><br>OSSK_404842	284.26	-0.89	> 1000

**83**	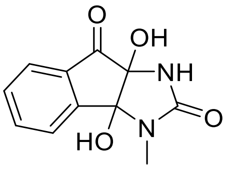<br>CN1C(NC2(C(C3=CC=CC=C3C12O)=O)O)=O<br>OSSK_451422	234.21	0.16	> 1000
**84**	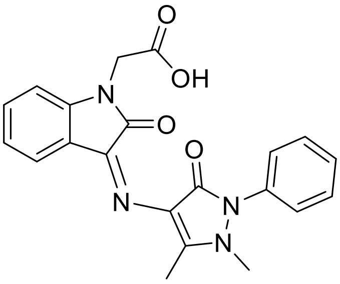<br>CN1N(C2=CC=CC=C2)C(C(/N=C3C(N(C4=CC=CC=C\34)CC(O)=O)=O)=C1C)=O<br>OSSK_488329	390.40	1.49	> 120
**85**	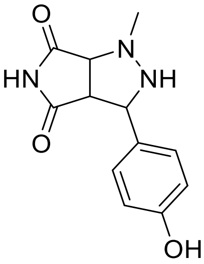<br>CN1NC(C2=CC=C(C=C2)O)C3C1C(NC3=O)=O<br>OSSK_645367	247.25	-0.57	> 120
**86**	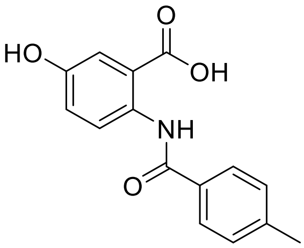<br>CC1=CC=C(C(NC2=CC=C(C=C2C(O)=O)O)=O)C=C1<br>OSSK_706039	271.27	3.58	> 120

**87**	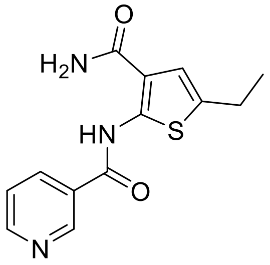<br>CCC1=CC(C(N)=O)=C(S1)NC(C2=CC=C N=C2)=O<br>OSSK_807467	275.33	2.38	> 60
**88**	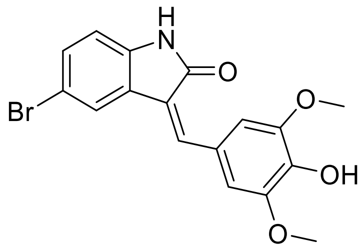<br>COC1=CC(/C=C2C(NC3=CC=C(C=C\23) Br)=O)=CC(OC)=C1O<br>OSSL_267571	376.21	3.42	> 120
**89**	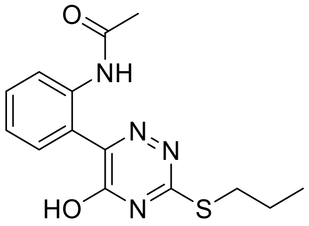<br>CCCSC1=NC(O)=C(C2=CC=CC=C2NC(C)=O)N=N1<br>OSSL_268532	304.37	2.13	> 120
**90**	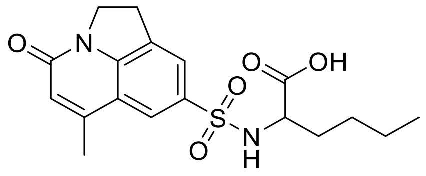<br>CCCCC(C(O)=O)NS(=O)(C1=CC2=C3N(C(C=C(C3=C1)C)=O)CC2)=O<br>OSSL_304616	378.44	2.09	> 60

**91**	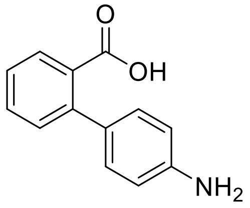<br>NC1=CC=C(C2=CC=CC=C2C(O)=O)C=C<br>1<br><br>OSSL_312689	213.34	2.45	> 1000
**92**	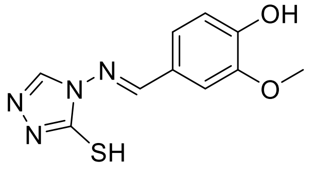<br>COC1=CC(/C=N/N2C=NN=C2S)=CC=C1<br>O<br><br>OSSK_001945	250.28	1.96	> 60

Table S2: Structures, SMILES codes and supplier ID of hits from the second round of LBVS.

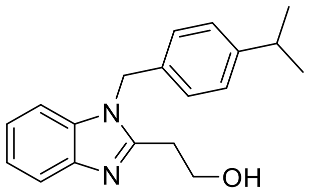

3

Cmpd. No.	Structure, SMILES and supplier ID	MW	cLogP	IC_{50} (μM)
**7**	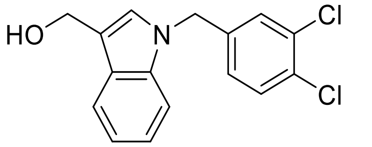 OCC1=CN(CC2=CC=C(Cl)C(Cl)=C2)C3=C1C=CC=C3<br>OSSL_652383	306.19	4.46	34 ± 4
**93**	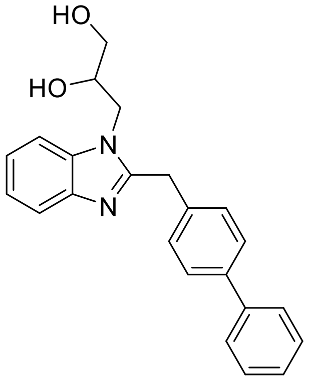 OC(CO)CN1C(CC2=CC=C(C3=CC=CC=C3)C=C2)=NC4=CC=CC=C41<br>OSSK_769093	358.44	3.77	381 ± 122
**94**	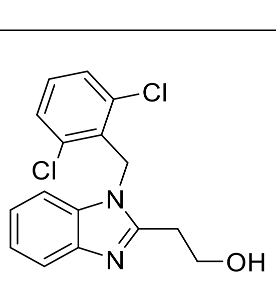 OCCC1=NC2=CC=CC=C2N1CC3=C(Cl)C=CC=C3Cl<br>OSSK_932460	321.20	3.96	465 ± 167

**95**	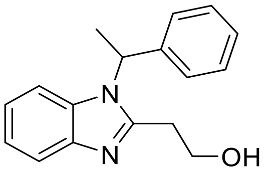 OCCC1=NC2=CC=CC=C2N1C(C)C3=CC=CC=C3 OSSK_286476	266.34	3.17	720 ± 252
**96**	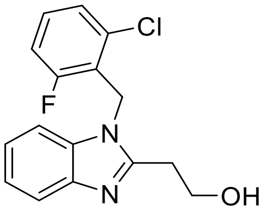 OCCC1=NC2=CC=CC=C2N1CC3=C(F)C=CC=C3Cl OSSK_932461	304.75	3.50	900 ± 390
**97**	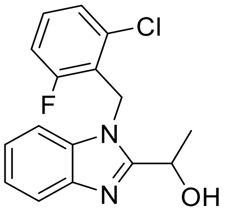 OC(C)C1=NC2=CC=CC=C2N1CC3=C(F)C=CC=C3Cl OSSK_464452	304.75	3.83	1100 ± 300
**98**	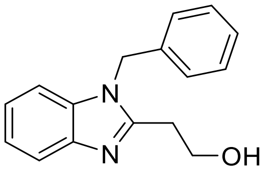 OCCC1=NC2=CC=CC=C2N1CC3=CC=CC=C3 OSSK_932458	252.32	2.75	2900 ± 600
**99**	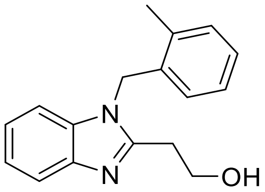	266.34	3.26	> 500

	OCCC1=NC2=CC=CC=C2N1CC3=CC=C C=C3C<br><br>OSSK_927258			
**100**	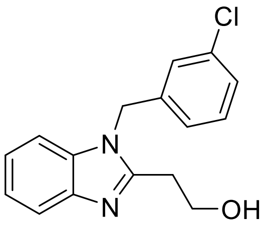<br>OCCC1=NC2=CC=CC=C2N1CC3=CC=C C(Cl)=C3<br><br>OSSK_927259	286.76	3.35	> 236
**101**	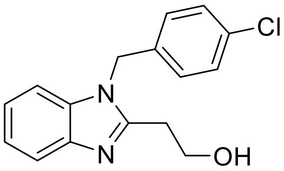<br>OCCC1=NC2=CC=CC=C2N1CC3=CC=C (Cl)C=C3<br><br>OSSK_932459	286.76	3.35	> 440
**102**	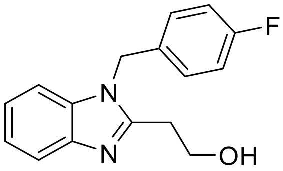<br>OCCC1=NC2=CC=CC=C2N1CC3=CC=C (F)C=C3<br><br>OSSK_932462	270.31	2.89	> 540
**103**	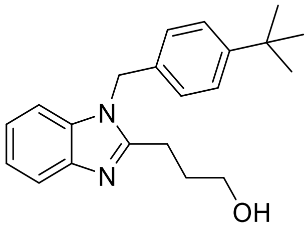<br>OCCCC1=NC2=CC=CC=C2N1CC3=CC= C(C(C)(C)C)C=C3<br><br>OSSK_286458	322.45	4.58	> 90

**104**	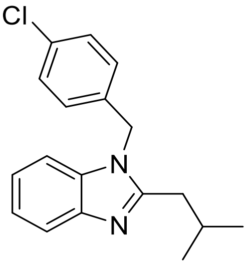<br>CC(C)CC1=NC2=CC=CC=C2N1CC3=CC<br>=C(Cl)C=C3<br><br>OSSK_286117	298.81	5.37	> 63
**105**	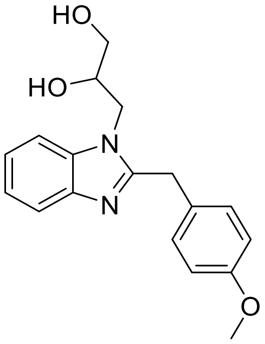<br>COC(C=C1)=CC=C1CC2=NC3=CC=CC=<br>C3N2CC(O)CO<br><br>OSSK_356610	312.37	1.96	> 2000

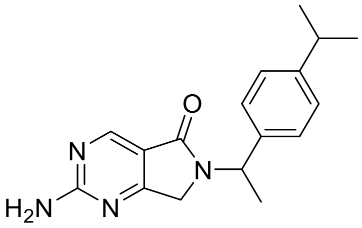

4

**Cmpd. No.**	**Structure, SMILES and supplier ID**	**MW**	**cLogP**	**IC$_{50}$ (μM)**
**8**	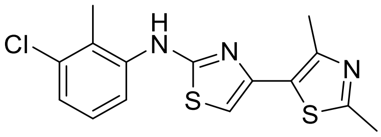<br>CC1=C(NC2=NC(C3=C(C)N=C(C)S3)=C<br>S2)C=CC=C1Cl<br><br>OSSK_523229	335.87	4.82	92 ± 17

**106**	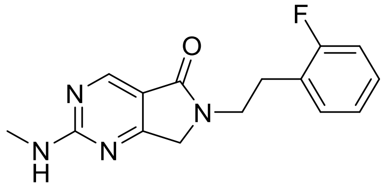 O=C1N(CCC2=CC=CC=C2F)CC3=NC(NC)=NC=C31 OSSL_317963	286.31	1.49	> 340
**107**	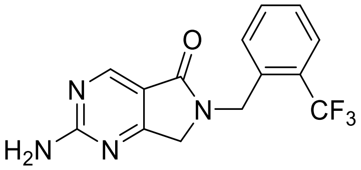 NC1=NC=C2C(CN(CC3=C(C(F)(F)F)C=CC=C3)C2=O)=N1 OSSL_317988	308.26	1.63	> 380
**108**	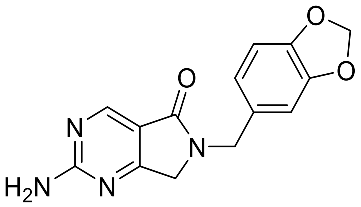 NC1=NC=C2C(CN(CC3=CC(OCO4)=C4C=C3)C2=O)=N1 OSSL_318222	284.28	0.38	> 260
	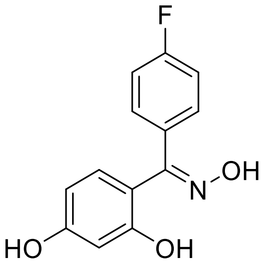 **5**			

Cmpd. No.	Structure, SMILES and supplier ID	MW	cLogP	IC_{50} (μM)
**9**	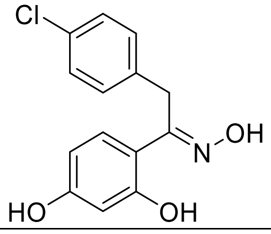	277.70	3.37	44 ± 8

	OC1=CC=C(/C(CC2=CC=C(Cl)C=C2)=N/<br>O)C(O)=C1<br><br>OSSK_862931			
**109**	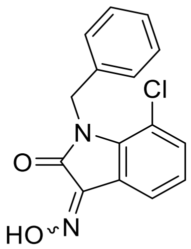<br>O=C(/C1=N\O)N(CC2=CC=CC=C2)C3=C<br>1C=CC=C3Cl<br><br>OSSL_327325	286.72	3.15	165 ± 21
**110**	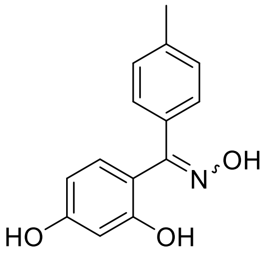<br>O/N=C(C1=CC=C(C)C=C1)/C2=CC=C(O)<br>C=C2O<br><br>OSSL_151910	243.26	3.35	> 1000
**111**	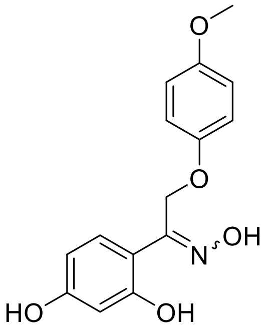<br>O/N=C(COC1=CC=C(OC)C=C1)/C2=CC<br>=C(O)C=C2O<br><br>OSSL_151915	289.29	2.29	> 300

**112**	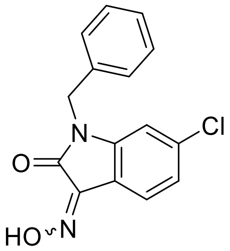<br>ClC1=CC(N(CC2=CC=CC=C2)C(/C3=N\O)=O)=C3C=C1<br>OSSL_327230	286.72	3.15	> 900
**113**	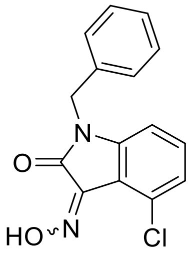<br>O=C(/C1=N\O)N(CC2=CC=CC=C2)C3=C1C(Cl)=CC=C3<br>OSSL_327325	286.72	3.15	> 30
**114**	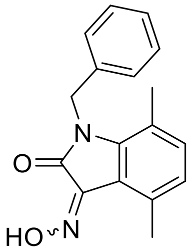<br>O=C(/C1=N\O)N(CC2=CC=CC=C2)C3=C1C(C)=CC=C3C<br>OSSL_328145	280.33	3.58	> 60
**115**	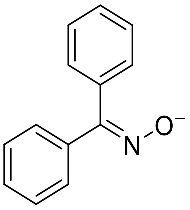<br>[O-]/N=C(C1=CC=CC=C1)/C2=CC=CC=C2<br>OSSL_379701	196.23	3.44	> 500

**116**	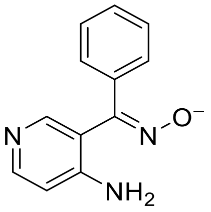 [O-]/N=C(C1=CC=CC=C1)/C2=CN=CC=C 2N OSSL_302816	212.23	1.40	> 2000
**117**	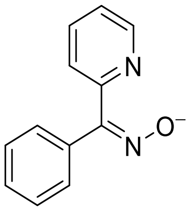 [O-]/N=C(C1=NC=CC=C1)/C2=CC=CC=C 2 OSSL_750176	197.22	2.61	> 500

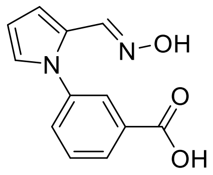

6

Cmpd. No.	Structure, SMILES and supplier ID	MW	cLogP	IC_{50} (μM)
**118**	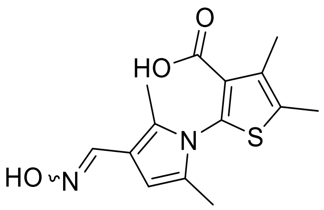 CC1=C(/C=N/O)C=C(C)N1C2=C(C(O)=O)C(C)=C(C)S2 OSSK_632565	292.35	3.82	> 800
**119**	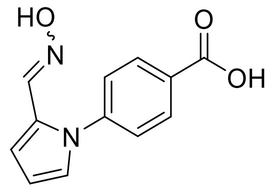	230.22	2.23	> 500

	OC(C1=CC=C(N2C(/C=N/O)=CC=C2)C=<br>C1)=O<br><br>OSSK_632073			
**120**	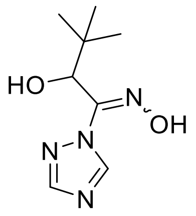<br><br>OC(C(C)(C)C)/C(N1C=NC=N1)=N/O<br><br>OSSL_128671	198.23	0.82	> 200
**121**	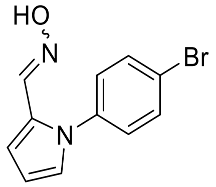<br><br>BrC1=CC=C(N2C(/C=N/O)=CC=C2)C=C<br>1<br><br>OSSK_777326	265.11	3.35	> 200
	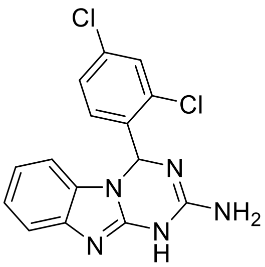<br>12			
**Cmpd. No.**	**Structure, SMILES and supplier ID**	**MW**	**cLogP**	**IC_{50} (μM)**
**122**	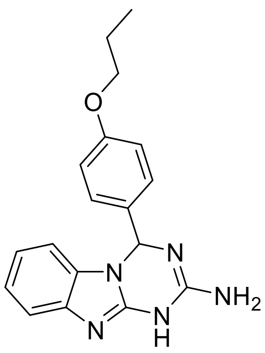<br><br>NC1=NC(C2=CC=C(OCCC)C=C2)N3C(N<br>1)=NC4=CC=CC=C34	321.38	3.97	> 50

	OSSK_784042			
**123**	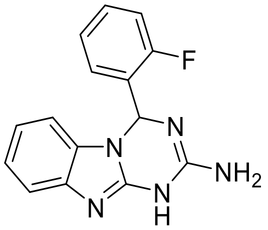<br>NC1=NC(C2=CC=CC=C2F)N3C(N1)=NC4=CC=CC=C34<br>OSSK_676133	281.29	2.94	> 120
**124**	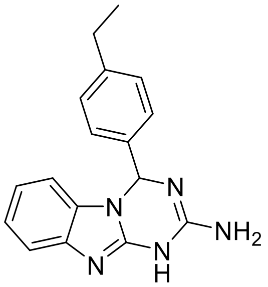<br>NC1=NC(C2=CC=C(CC)C=C2)N3C(N1)=NC4=CC=CC=C34<br>OSSK_676805	291.36	4.20	> 30
**125**	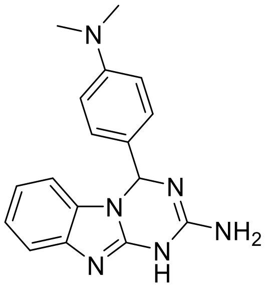<br>NC1=NC(C2=CC=C(N(C)C)C=C2)N3C(N1)=NC4=CC=CC=C34<br>OSSK_676809	306.37	2.90	> 200
**126**	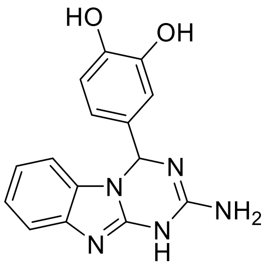<br>NC1=NC(C2=CC=C(O)C(O)=C2)N3C(N1)=NC4=CC=CC=C34	295.30	2.19	> 160

OSSL_009162

Table S3: Structures, SMILES codes and supplier ID of hits from the third round of LBVS.

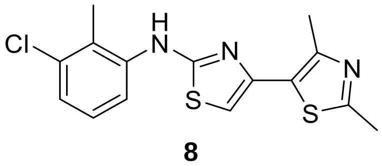

8

Cmpd. No.	Structure, SMILES and supplier ID	MW	cLogP	IC_{50} (µM)
**10**	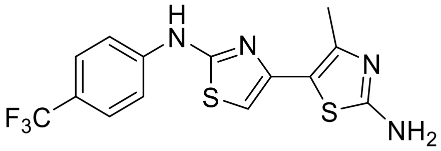 CC1=C(SC(N)=N1)C2=CSC(NC3=CC=C(C(F)(F)F)C=C3)=N2 OSSL_650294	356.39	4.31	32 ± 3
**26**	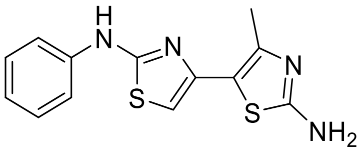 CC1=C(SC(N)=N1)C2=CSC(NC3=CC=CC=C3)=N2 OSSK_672441	288.39	3.43	> 120
**27**	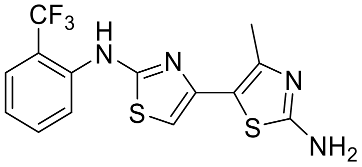 CC1=C(SC(N)=N1)C2=CSC(NC3=C(C(F)(F)F)C=CC=C3)=N2 OSSL_648433	356.39	4.31	45 ± 4
**28**	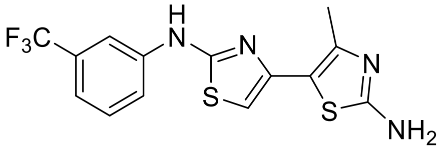	356.39	4.31	56 ± 9

	CC1=C(SC(N)=N1)C2=CSC(NC3=CC(C(F)(F)F)=CC=C3)=N2<br><br>OSSK_523240			
**29**	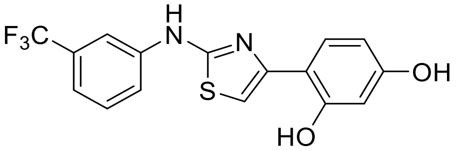<br>OC(C=C(O)C=C1)=C1C2=CSC(NC3=CC(C(F)(F)F)=CC=C3)=N2<br><br>OSSK_137545	352.33	5.06	48 ± 9
**30**	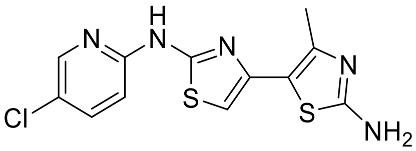<br>CC1=C(SC(N)=N1)C2=CSC(NC3=NC=C(Cl)C=C3)=N2<br><br>OSSL_648436	323.82	3.41	54 ± 7
**31**	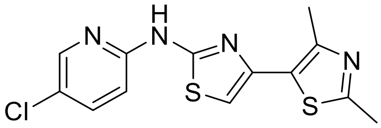<br>CC1=C(SC(C)=N1)C2=CSC(NC3=NC=C(Cl)C=C3)=N2<br><br>OSSL_631964	322.83	3.68	> 500
**127**	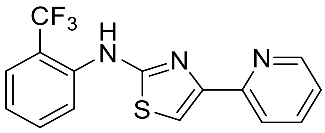<br>FC(C1=C(NC2=NC(C3=CC=CC=N3)=CS2)C=CC=C1)(F)F<br><br>OSSL_648411	321.32	4.84	86 ± 5
**128**	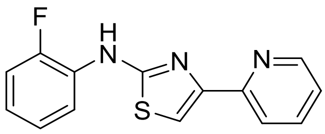<br>FC1=C(NC2=NC(C3=CC=CC=N3)=CS2)C=CC=C1	271.31	4.10	100 ± 27

	OSSL_648413			
**129**	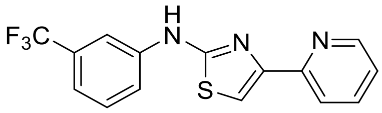<br>FC(C1=CC=CC(NC2=NC(C3=CC=CC=N3)=CS2)=C1)(F)F<br>OSSL_648412	321.32	4.84	168 ± 18
**130**	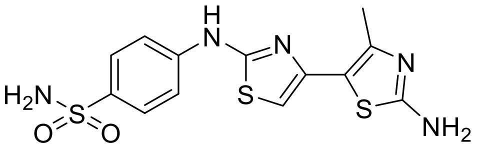<br>CC1=C(SC(N)=N1)C2=CSC(NC3=CC=C(S(N)(=O)=O)C=C3)=N2<br>OSSK_320567	367.46	2.04	315 ± 47
**131**	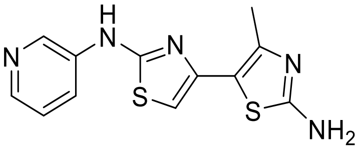<br>CC1=C(SC(N)=N1)C2=CSC(NC3=CN=CC=C3)=N2<br>OSSL_648437	289.38	2.21	377 ± 102
**132**	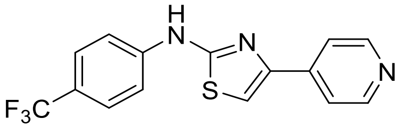<br>FC(C1=CC=C(NC2=NC(C3=CC=NC=C3)=CS2)C=C1)(F)F<br>OSSL_648422	321.32	4.45	> 60
**133**	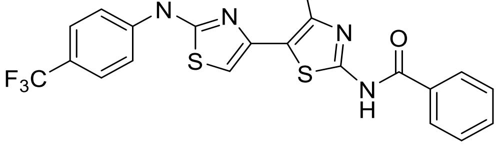<br>CC1=C(SC(NC(C2=CC=CC=C2)=O)=N1)C3=CSC(NC4=CC=C(C(F)(F)F)C=C4)=N3<br>OSSK_630272	460.49	6.23	> 120

**134**	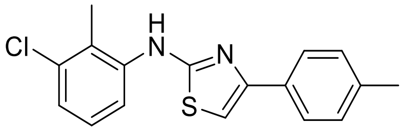<br>CC1=C(NC2=NC(C3=CC=C(C)C=C3)=CS2)C=CC=C1Cl<br>OSSL_960616	314.83	6.42	> 16
**135**	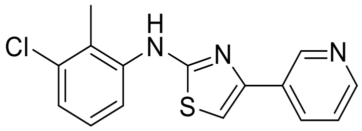<br>CC1=C(NC2=NC(C3=CC=CN=C3)=CS2)C=CC=C1Cl<br>OSSK_523203	301.79	4.69	> 200

Table S4: Toxicity of **9** and **10** on zebrafish larvae.

Cmpd. No.	Conc. [µM]	24 h (2 dpf)	48 h (3 dpf)	72 h (4 dpf)	96 h (5 dpf)
**9**	100	5	2	0	0
	30	10	10	10	10
	10	10	10	10	10
	2	10	10	10	10
**10**	100	0	0	0	0
	30	2	0	0	0
	10	10	10	10	10
	2	10	10	10	10
DMSO	1 %	10	10	10	10
Danieau's	-	10	10	10	10

Fish line: TLF. Incubation with compounds started at 1 dpf. Visual screening (microscopy) daily until 5 dpf. 10 larvae per condition. The number of living zebrafish larvae is given. Dpf, days post fertilization.

Table S5: MIC values for final hits against *E. coli* TolC strain.

Cmpd. No.	7	9	10
**MIC [µM]**	22.0 ± 1.0	21.9 ± 1.3	22.2 ± 3.1

Table S6: Frequency-of-resistance development of **10** and selected antibiotics (rifampicin, chloramphenicol and ciprofloxacin) at 4x and 2x MIC.

Cmpd.	4x MIC	2x MIC
**10**	$< 8.7 * 10^{-10}$	$3.3 * 10^{-9}$
**Rif**	$1.7 * 10^{-9}$	$3.8 * 10^{-9}$
**Cam**	$< 8.7 * 10^{-10}$	$2.9 * 10^{-10}$
**Cip**	$1.7 * 10^{-9}$	$7.5 * 10^{-9}$

Rif, Rifampicin; Cam, Chloramphenicol; Cip, Ciprofloxacin.
